# *In-Silico* Detection of Oral Prokaryotic Species With Highly Similar 16S rRNA Sequence Segments Using Different Primer Pairs

**DOI:** 10.3389/fcimb.2021.770668

**Published:** 2022-02-09

**Authors:** Alba Regueira-Iglesias, Lara Vázquez-González, Carlos Balsa-Castro, Triana Blanco-Pintos, Benjamín Martín-Biedma, Víctor M. Arce, Maria J. Carreira, Inmaculada Tomás

**Affiliations:** ^1^ Oral Sciences Research Group, Department of Surgery and Medical-Surgical Specialties, School of Medicine and Dentistry, Universidade de Santiago de Compostela, Health Research Institute Foundation of Santiago (FIDIS), Santiago de Compostela, Spain; ^2^ Centro Singular de Investigación en Tecnoloxías Intelixentes and Departamento de Electrónica e Computación, Universidade de Santiago de Compostela, Health Research Institute Foundation of Santiago (FIDIS), Santiago de Compostela, Spain; ^3^ Department of Physiology and Center for Disease in Molecular Medicine and Chronic Diseases, Universidade de Santiago de Compostela, Santiago de Compostela, Spain

**Keywords:** computational biology, DNA primers, genes, high-throughput nucleotide sequencing, mouth, microbiota, 16S rRNA

## Abstract

Although clustering by operational taxonomic units (OTUs) is widely used in the oral microbial literature, no research has specifically evaluated the extent of the limitations of this sequence clustering-based method in the oral microbiome. Consequently, our objectives were to: 1) evaluate *in-silico* the coverage of a set of previously selected primer pairs to detect oral species having 16S rRNA sequence segments with ≥97% similarity; 2) describe oral species with highly similar sequence segments and determine whether they belong to distinct genera or other higher taxonomic ranks. Thirty-nine primer pairs were employed to obtain the *in-silico* amplicons from the complete genomes of 186 bacterial and 135 archaeal species. Each fasta file for the same primer pair was inserted as subject and query in BLASTN for obtaining the similarity percentage between amplicons belonging to different oral species. Amplicons with 100% alignment coverage of the query sequences and with an amplicon similarity value ≥97% (ASI97) were selected. For each primer, the species coverage with no ASI97 (SC-NASI97) was calculated. Based on the SC-NASI97 parameter, the best primer pairs were OP_F053-KP_R020 for bacteria (region V1-V3; primer pair position for *Escherichia coli* J01859.1: 9-356); KP_F018-KP_R002 for archaea (V4; undefined-532); and OP_F114-KP_R031 for both (V3-V5; 340-801). Around 80% of the oral-bacteria and oral-archaea species analyzed had an ASI97 with at least one other species. These very similar species play different roles in the oral microbiota and belong to bacterial genera such as *Campylobacter*, *Rothia*, *Streptococcus* and *Tannerella*, and archaeal genera such as *Halovivax*, *Methanosarcina* and *Methanosalsum*. Moreover, ~20% and ~30% of these two-by-two similarity relationships were established between species from different bacterial and archaeal genera, respectively. Even taxa from distinct families, orders, and classes could be grouped in the same possible OTU. Consequently, regardless of the primer pair used, sequence clustering with a 97% similarity provides an inaccurate description of oral-bacterial and oral-archaeal species, which can greatly affect microbial diversity parameters. As a result, OTU clustering conditions the credibility of associations between some oral species and certain health and disease conditions. This significantly limits the comparability of the microbial diversity findings reported in oral microbiome literature.

## Introduction

Since the introduction of the Sanger method in 1977, the sequencing technologies have undergone substantial improvements as the automatization and parallelization, which have allowed the characterization of the microbiomes to unprecedented depths rapidly and cost-effectively (Midha et al., 2019). At present, the targeted amplicon sequencing of the phylogenetic marker 16S ribosomal RNA (rRNA) gene is, by far, one of the most commonly used techniques to determine the structure and composition of the prokaryote communities (Davidson and Epperson, 2018). 

Studies published during the last decade have assessed the mouth’s microbiome using high throughput 16S rRNA gene sequencing (Zaura et al., 2021). To facilitate the analysis of complex microbial communities like the oral environment, amplicons derived from this technology are typically clustered into operational taxonomic units (OTUs) that are intended to correspond to taxonomic clades or monophyletic groups (Edgar, 2013). Specifically, sequences are clustered based on a given similarity threshold, usually set at 97%, which has been conventionally regarded as the species-level correspondent (Stackebrandt and Goebel, 1994; Zaura et al., 2021).

Numerous OTU clustering algorithms have been integrated into the popular sequence-analysis pipelines, such as QIIME2 (Bolyen et al., 2019), mothur (Schloss et al., 2009), and USEARCH (Edgar, 2010). Overall, existing methods for grouping 16S rRNA gene amplicons into OTUs can be categorized in three ways: *de novo*, closed-reference, and open-reference (Wei et al., 2021). While the first approach groups amplicons based on pairwise sequence distances, the second approach groups sequences that match a reference sequence from a database in the same OTU. The open-reference represents a combination of the two others, where sequences that do not adequately match the reference are grouped using a *de novo* method (Wei et al., 2021). However, none of these approaches produce the same results in terms of obtaining OTUs, even when using the same dataset (He et al., 2015; Westcott and Schloss, 2015). Moreover, even the same method can yield distinct results after only a minor parameter change (Wei et al., 2021).

In addition, it has been reported that different species can have very highly similar 16S rRNA gene sequences (Větrovský and Baldrian, 2013; Schloss, 2021), which may lead to the grouping of distinct taxa in the same OTU. In fact, around 25% of OTUs constructed using the widely adopted ≥97% similarity threshold have been found to contain gene sequences from multiple species (Větrovský and Baldrian, 2013; Schloss, 2021). These estimates were slightly different depending on the gene region studied but reached up to 35% for variable regions V4-V5 (Schloss, 2021). Consequently, the construction of an OTU table can be affected, as can, by extension, taxonomic assignments, and microbial diversity results.

Due to the limitations of OTU clustering, other analyses based on establishing 100% sequence identity or single-nucleotide resolution have been proposed, such as zero-radius operational taxonomic units (Edgar, 2016), oligotypes, or minimum entropy decomposition nodes (Eren et al., 2015), amplicon sequence variants (Callahan et al., 2016) or suboperational taxonomic units (Amir et al., 2017). The most widely known pipelines based on the single-nucleotide resolution are DADA2 (Callahan et al., 2016), Deblur (Amir et al., 2017), and UNOISE (Edgar, 2016). These attempt to model the error of the sequencer and to cluster reads in a way that their distribution within clusters is consistent with such error (Caruso et al., 2019); however, they differ in how this correction is done (Nearing et al., 2018).

Several investigations have compared the two clustering approaches (OTUs *versus* single-nucleotide resolution) to discern which performs better (Nearing et al., 2018; Caruso et al., 2019; Prodan et al., 2020; Abellan-Schneyder et al., 2021; García-López et al., 2021; Schloss, 2021). In general, the pipelines based on the single-nucleotide resolution have demonstrated superior sensitivity, specificity, and precision, and lower spurious sequence rates when compared to OTU algorithms (Caruso et al., 2019; Prodan et al., 2020). They allow for easier inter-study integration of biological features as amplicon sequence variants have intrinsic meaning independent of the reference database used, contrary to the study-specific nature of OTUs (Callahan et al., 2017; Prodan et al., 2020). However, single-nucleotide resolution algorithms, when analyzing 16S rRNA gene data, can split a single genome into separate clusters (Schloss, 2021). Furthermore, there is no consensus regarding the influence of the method chosen on the diversity results obtained. Meanwhile, some authors obtained minor differences between pipelines using the two clustering methods, with comparable alpha- and beta-diversity profiles (Abellan-Schneyder et al., 2021; García-López et al., 2021); others evidenced distinct results even among those from the same approach (Prodan et al., 2020).

Currently, more than 80% of recent studies on the oral microbiome performed their analyses based on OTU clustering. However, to our knowledge, no research has specifically evaluated the extent of the limitations of these sequence clustering-based methods by a similarity threshold in the oral microbiome. Consequently, the objectives of the present *in-silico* investigation were to: 1) evaluate the coverage of a set of previously selected primer pairs to detect oral species having 16S rRNA sequence segments with ≥97% similarity; 2) describe oral species with highly similar sequence segments and determine whether they belong to distinct genera or other higher taxonomic ranks.

## Materials and Methods

**Figure 1:**
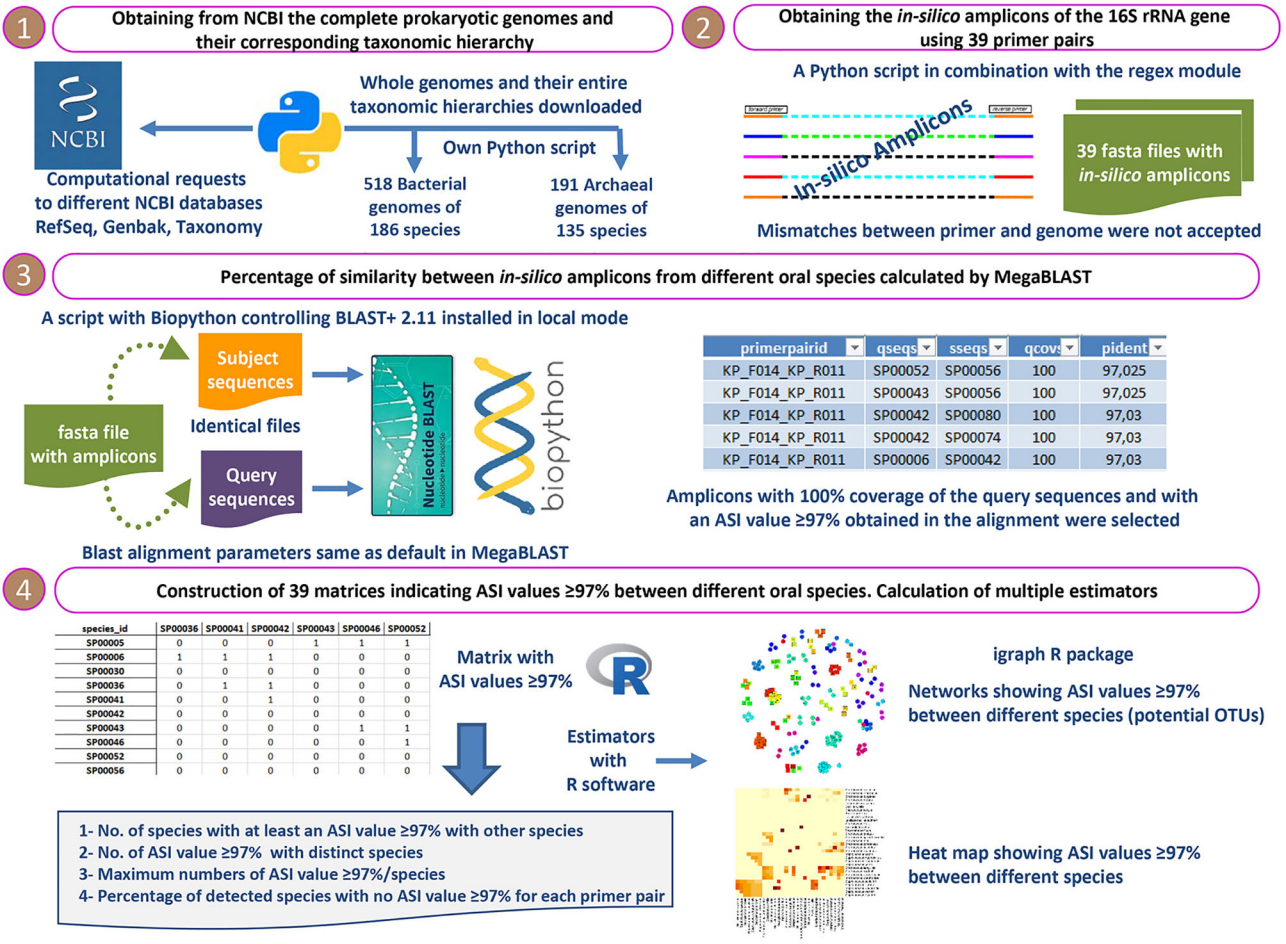
The complete analysis protocol applied in the present *in-silico* study.

The complete analysis protocol applied in the present study is detailed in **Figure 1**.

### Obtaining Complete Genomes of Oral Bacteria and Oral Archaea

The information on the bacterial taxa present in the oral cavity was obtained from the eHOMD website (Escapa et al., 2018). Of the 2074 genomes available on the site, we only selected the 518 that had a complete sequencing status for use in the research. These complete genomes have one or more GenBank identifiers (Clark et al., 2016), which were employed to access the complete sequences stored in the NCBI database (NCBI Resource Coordinators, 2016). Additionally, an initial list of 177 different oral archaea and their corresponding GenBank identifiers (Clark et al., 2016), obtained as part of previous research conducted by our group (Regueira-Iglesias et al., 2021a), enabled us to access their complete sequences and annotations in the NCBI database. Integrating the “Entrez Programming Utilities (E-utilities)” tool (National Center for Biotechnology Information, 2010) in our Python script (Python Software Foundation, 2020) allowed us to acquire the URLs needed to retrieve the information of interest from various NCBI databases, including Taxonomy (Schoch et al., 2020), RefSeq (O'Leary et al., 2016), and GenBank (Clark et al., 2016).

Therefore, a total of 709 complete prokaryotic genomes from a total of 321 oral species were downloaded (more than one complete genome was analyzed for several species). Finally, the complete taxonomic hierarchy (from superkingdom to strain) of all downloaded complete genomes was designated by the taxonomic identifier included in the annotated information in the NCBI database, all computationally performed with our script above.

### Selecting the Primer Pairs and Obtaining the *In-Silico* Amplicons of the 16S rRNA Gene

Thirty-three primer pairs with the best *in-silico* coverage, as identified in earlier research by our group, were selected, along with the six primer pairs used the most in the oral-microbiome literature (Regueira-Iglesias et al., 2021a). These primer pairs were classified according to the mean length of their amplicons: short primer pairs (S, 100-300 base pairs), medium primer pairs (M, 301-600 bps), and long primer pairs (L, >600 bps); and the domain targeted (bacteria, archaea, or both) (**Table 1**).

**Table 1:** Selected primer pairs with high *in-silico* coverage percentages targeting oral bacteria and/or archaea and those most used in the sequencing-based studies of the oral microbiome.

Bacterial-specific primer pair	ALC	F identifier	F Sequence 5-3	F First post	F Last Post	R identifier	R Sequence 5-3	R First post	R Last Post	Length (bps)	Region
	**S**	KP_F048	TACGGRAGGCAGCAG	342	356	OP_R043	CCGCGRCTGCTGGCAC	514	529	187	V3-V4
		OP_F098	CCAGCAGCYGCGGTAAN	517	533	OP_R119	GGACTACCRGGGTATCTAA	787	805	288	V4-V5
		OP_F066	GGMTTAGATACCC	784	796	KP_R040	CCGTCAATTCMTTTGAGTTT	906	925	141	V5-V6
		OP_F009	GGATTAGATACCCBRGTAGTC	784	804	OP_R030	TCACRRCACGAGCTGWCGAC	1060	1079	295	V5-V7
		KP_F061	ACTCAAAKGAATWGACGG	908	925	KP_R074	GGGTYKCGCTCGTTR	1099	1113	205	V6-V7
		OP_F101	GAATTGRCGGGGRCC	916	930	OP_R030	TCACRRCACGAGCTGWCGAC	1060	1079	163	V6-V7
	**M**	OP_F053	GRGTTYGATYMTGGCTCAG	9	27	KP_R020	CTGCTGCCTYCCGTA	342	356	347	V1-V3
		KP_F048	TACGGRAGGCAGCAG	342	356	KP_R031	TACHVGGGTATCTAAKCC	784	801	459	V3-V5
		KP_F048	TACGGRAGGCAGCAG	342	356	OP_R073	CRTACTHCHCAGGYG	879	893	551	V3-V6
		KP_F051	GTGCCAGCMGCNGCGG	514	529	KP_R041	CGTCAATTCMTTTGAGTT	907	924	410	V4-V6
		KP_F051	GTGCCAGCMGCNGCGG	514	529	OP_R030	TCACRRCACGAGCTGWCGAC	1060	1079	565	V4-V7
		OP_F116	YAACGAGCGCAACCC	1099	1113	KP_R060	GACGGGCGGTGWGTRCA	1390	1406	307	V7-V9
	**L**	KP_F048	TACGGRAGGCAGCAG	342	356	OP_R030	TCACRRCACGAGCTGWCGAC	1060	1079	737	V3-V7
		KP_F048	TACGGRAGGCAGCAG	342	356	KP_R060	GACGGGCGGTGWGTRCA	1390	1406	1064	V3-V9
		KP_F056	AYTGGGYDTAAAGNG	572	576	KP_R077	GACGGGCGGTGTGTACAA	1389	1406	834	V4-V9
**Archaeal-specific primer pair**	**ALC**	**F identifier**	**F Sequence 5-3**	**F First post**	**F Last Post**	**R identifier**	**R Sequence 5-3**	**R First post**	**R Last Post**	**Length (bps)**	**Region**
	**S**	KP_F018	GYGCASCAGKCGMGAAW	U	U	KP_R002	TTACCGCGGCKGCTG	518	532	–	- V4
		OP_F066	GGMTTAGATACCC	784	796	KP_R013	GGCCATGCACCWCCTCTC	U	U	–	V5-V6
	**M**	KP_F018	GYGCASCAGKCGMGAAW	U	U	KP_R032	TACNVGGGTATCTAATCC	784	801	–	V3-V5
		KP_F018	GYGCASCAGKCGMGAAW	U	U	OP_R073	CRTACTHCHCAGGYG	879	893	–	V3-V5
		KP_F020	CAGCMGCCGCGGTAA	518	532	KP_R013	GGCCATGCACCWCCTCTC	U	U	–	V3-V6
		KP_F022	AGGAATTGGCGGGGGAGCA	U	U	KP_R063	TACCTTGTTACGACTT	1491	1506	–	V5-V9
	**L**	OP_F114	CCTAYGGGRBGCASCAG	340	356	KP_R013	GGCCATGCACCWCCTCTC	U	U	–	V3-V6
		KP_F018	GYGCASCAGKCGMGAAW	U	U	KP_R063	TACCTTGTTACGACTT	1491	1506	–	V3-V9
		OP_F066	GGMTTAGATACCC	784	796	OP_R016	CGGTGTGTGCAAGGAG	U	U	–	V5-V9
**Bacterial and archaeal primer pair**	**ALC**	**F identifier**	**F Sequence 5-3**	**F First post**	**F Last Post**	**R identifier**	**R Sequence 5-3**	**R First post**	**R Last Post**	**Length (bps)**	**Region**
	S	OP_F114	CCTAYGGGRBGCASCAG	340	356	KP_R002	TTACCGCGGCKGCTG	518	532	192	V3-V4
		KP_F020	CAGCMGCCGCGGTAA	518	532	KP_R032	TACNVGGGTATCTAATCC	784	801	283	V4-V5
		OP_F066	GGMTTAGATACCC	784	796	OP_R073	CRTACTHCHCAGGYG	879	893	109	V5-V6
	M	OP_F114	CCTAYGGGRBGCASCAG	340	356	KP_R031	TACHVGGGTATCTAAKCC	784	801	461	V3-V5
		OP_F114	CCTAYGGGRBGCASCAG	340	356	OP_R073	CRTACTHCHCAGGYG	879	893	553	V3-V6
		KP_F020	CAGCMGCCGCGGTAA	518	532	OP_R073	CRTACTHCHCAGGYG	879	893	375	V4-V6
	L	OP_F114	CCTAYGGGRBGCASCAG	340	356	OP_R121	ACGGGCGGTGWGTRC	1391	1405	1065	V3-V9
		KP_F020	CAGCMGCCGCGGTAA	518	532	OP_R121	ACGGGCGGTGWGTRC	1391	1405	887	V4-V9
		OP_F066	GGMTTAGATACCC	784	796	OP_R121	ACGGGCGGTGWGTRC	1391	1405	621	V5-V9
**Most used primer pair**	**ALC**	**F identifier**	**F Sequence 5-3**	**F First post**	**F Last Post**	**R identifier**	**R Sequence 5-3**	**R First post**	**R Last Post**	**Length (bps)**	**Region**
	S	KP_F078	GTGCCAGCMGCCGCGGTAA	514	532	OP_R010	GGACTACHVGGGTWTCTAAT	786	805	291	V4-V5
	M	KP_F031	AGAGTTTGATCCTGGCTCAG	8	27	KP_R021	TTACCGCGGCTGCTGGCAC	515	532	524	V1-V4
		KP_F047	CCTACGGGNGGCWGCAG	340	356	KP_R035	GACTACHVGGGTATCTAATCC	784	804	464	V3-V5
		OP_F009	GGATTAGATACCCBRGTAGTC	784	868	OP_R029	ACGTCRTCCCCDCCTTCCTC	1174	1193	409	V5-V8
	L	KP_F014	TCCAGGCCCTACGGG	U	U	KP_R011	YCCGGCGTTGAMTCCAATT	U	U	–	V3-V6
		KP_F034	AGAGTTTGATCMTGGCTCAG	8	27	KP_R065	TACGGYTACCTTGTTACGACTT	1491	1512	1504	V1-V9

Primer pairs were selected based on the species coverage values (number of species detected/total species evaluated) in a previous investigation (Regueira-Iglesias et al., 2021a). They were individually evaluated through regular expressions against Escherichia coli J01859 to define their positions. The U values represent a mismatch on the assessment and, therefore, the position cannot be confirmed with a guarantee. Gene regions were delimited as described by Baker et al. (Baker et al. 2003). ALC, amplicon length category; bps, base pairs; F, forward; KP, Klindworth primer; L, long mean amplicon length category, >600 base pairs; M, medium mean amplicon length category, 301-600 base pairs; OP, oral primer; Post, position; R, reverse; S, short mean amplicon length category, 100-300 base pairs; U, unidentified.

Applying our script in combination with Python’s regex module (Barnett, 2020), the direct and reverse sequences of each primer pair were used to obtain *in-silico* sequence segments of the whole genomes analyzed (hereafter referred to as *in-silico* amplicons). An *in-silico* amplicon was considered for subsequent analysis when the following conditions were present: 1) there is a zero mismatch of both primers (forward and reverse) of each pair; 2) the distance between the starting position of the forward primer and the ending position of the reverse primer is less than 2300 and higher than 100 nucleotides; 3) the *in-silico* amplicon does not repeat within the same species.

All *in-silico* amplicons from the same species, even if from different strains, were considered for analysis. For each primer pair, a fasta file was created where all *in-silico* amplicons found were stored. The stored sequences were identified with the same taxonomic hierarchy as the genomes from which they were detected. As many *in silico*-amplicons were detected within the same species (differing by at least 1 nucleotide), the sequence variants were identified with correlative numbering at a new hierarchical level below the species name.

In the fasta files, all sequences included a species identifier (SPn) and a variant identifier (Vn) in their header, and then the header of each sequence also included the taxonomic hierarchy up to the variant level within each species. Finally, the *in-silico* amplicons were obtained from 186 oral-bacterial and 135 oral-archaeal species.

### Determination of the Percentage of Similarity Between *In-Silico* Amplicons of Different Oral Species by MegaBLAST

A script with the NcbiblastnCommandline wrapper from Biopython (Cock et al., 2009) was developed to manage BLAST+ 2.11 (Camacho et al., 2009) in the local mode from Biopython. This enabled the data obtained in the alignments to be easily transferred for later analysis on Python. The alignment parameters were configured to be the same as the default settings in MegaBLAST (Altschul et al., 1990) since these settings were appropriate for the alignment between sequences with a similarity ≥95%.

All sequences belonging to the same fasta file for the same primer pair were aligned against themselves; in order to do this, each fasta file was inserted as subject and query in BLASTN (Chen et al., 2015) for obtaining the percentage of similarity between *in-silico* amplicons belonging to different oral species.

From the results obtained, *in-silico* amplicons with 100% alignment coverage of the query sequences and with a similarity value ≥97% were selected. That is, alignments with the following BLAST+ estimates (Camacho et al., 2009): qcovs= 100%, qcovhsp= 100%, qcovus= 100%, and pident ≥97% were selected. Of the above alignments obtained, the following were discarded as they were not of interest: 1) *in-silico* amplicons with the same unique identifier (SPn + Vn); 2) *in-silico* amplicons with the same species identifier; and 3) duplicate alignments.

If two different species had more than one *in-silico* amplicon similarity value ≥97% (ASI97) among them, one of the alignments was chosen at random. The results of the highly similar species pairs, including taxonomic hierarchy data for both species, were then stored using the pandas (McKinney, 2010) and xlsxwriter (McNamara, 2013) Python modules.

### Construction of a Matrix With Oral Species Showing *In-Silico* Amplicon Similarity Values ≥97% and Calculation of Descriptive Statistical Estimators

A similarity matrix was created for each primer pair, where rows and columns had the species identifiers, and cells indicated with a number 1 the presence of an ASI97 between two different species. We then developed a script in R (R Core Team, 2020) through which we calculated the following estimates for each analyzed primer pair: 1) the number of species with at least one ASI97 with other species; 2) the total number of ASI97 between different species; 3) the mean and maximum numbers of ASI97 per species. In addition, we estimated the percentage of detected species (species coverage, SC) and the percentage of detected species without ASI97 for each primer pair (species coverage no ASI97, SC-NASI97). This last parameter was then used as a criterion for selecting the primers associated with a smaller number of oral species that may be erroneously clustered. The SC-NASI97 parameter will be influenced not only by the number of species with ASI, but also by the coverage percentages of each primer pair.

Finally, the bacterial and archaeal species pairs that showed an ASI97 were described and assessed whether they belonged to different genera or higher taxonomic ranks.

## Results

### Evaluation of the Primer Pairs for Detecting Oral Species With *In-Silico* Amplicon Similarity Values ≥97%

The primer pairs that targeted bacteria had a mean of 91.88 (49.40%) bacterial species with an ASI97 and an average of 153.46 ASI97 containing distinct species. For those targeting archaea, these numbers were 65.60 (48.59%) and 162.26, respectively. If the primers used most in the oral microbiome literature were excluded, those with short amplicon lengths (unlike the SC percentages) had the lowest SC-NASI97 values for both bacteria (S= 39.54%) and archaea (S= 40.44%) compared to the medium length and long primers (M= 45.82% and 46.35%, respectively; L= 48.39% and 44.32%, respectively).

**Figure 2:**
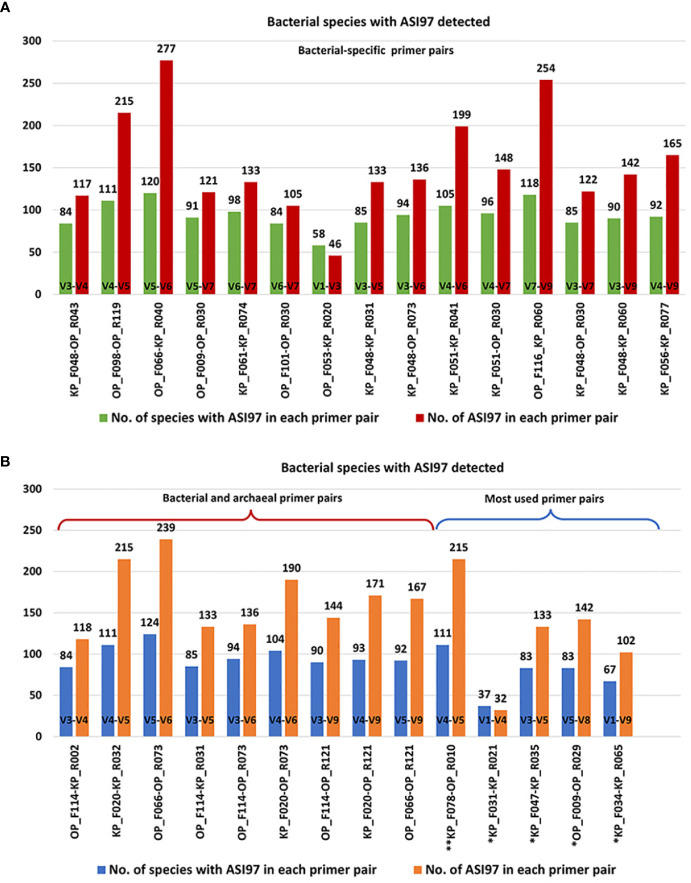
Number of bacterial species with *in-silico* amplicon similarity values ≥97% and number of *in-silico* amplicon similarity values ≥97% with the primer pairs evaluated against the oral bacteria genomes. **(A)** Estimates were obtained by the selected bacterial-specific primer pairs. **(B)** Estimates were obtained by the selected bacterial and archaeal primer pairs and the primer pairs used the most in the oral microbiome literature. Among the most commonly used primer pairs in the literature, those marked with an * are bacterial-specific and those with ** target both bacterial and archaea. ASI97, *in-silico* amplicon similarity values ≥97%; F, forward; KP, Klindworth primer; No., number; OP, oral primer; R, reverse.

**Figure 3:**
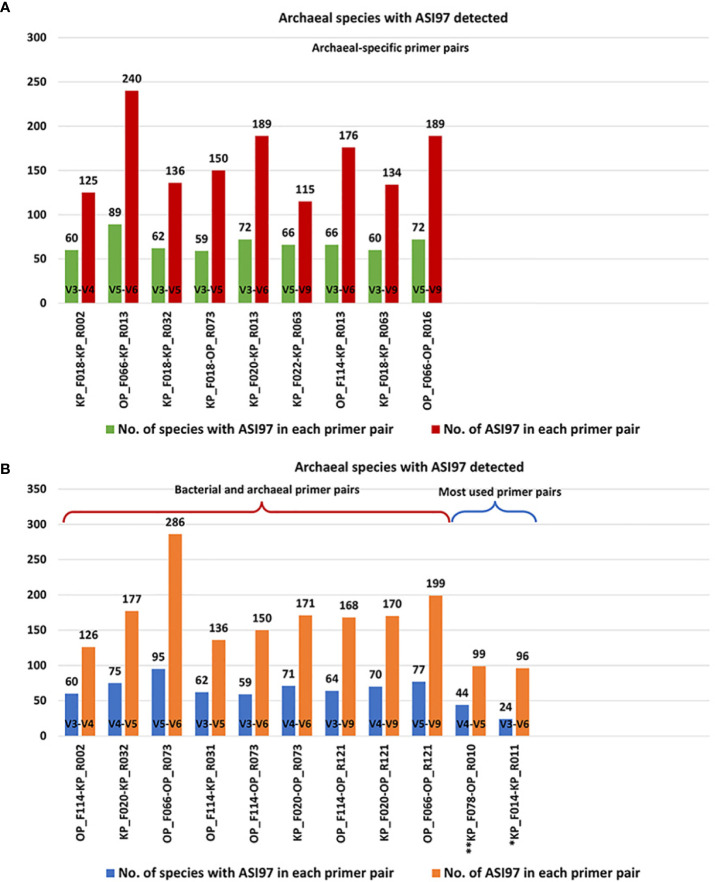
Number of archaeal species with *in-silico* amplicon similarity values ≥97% and number of *in-silico* amplicon similarity values ≥97% with the primer pairs evaluated against the oral archaea genomes. **(A)** Estimates were obtained by the selected archaeal-specific primer pairs. **(B)** Estimates were obtained by the selected bacterial and archaeal primer pairs and the primer pairs used the most in the oral microbiome literature. Among the most commonly used primer pairs in the literature, those marked with an * are archaeal-specific and those with ** target both bacterial and archaea. ASI97, *in-silico* amplicon similarity values ≥97%; F, forward; KP, Klindworth primer; No., number; OP, oral primer; R, reverse.

**Figure 4:**
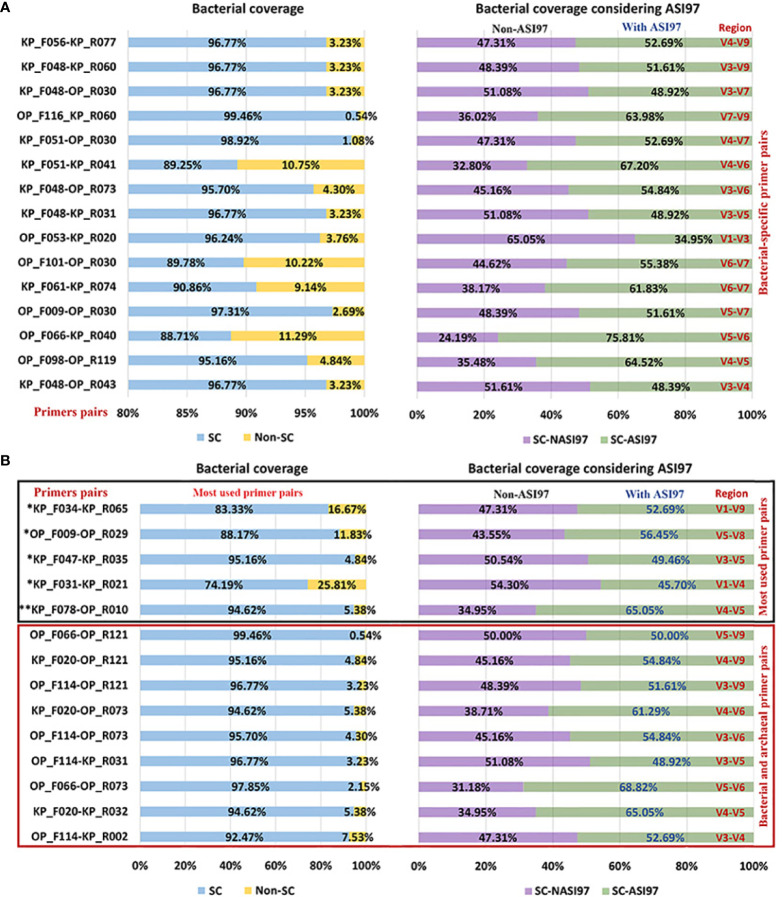
Percentages of coverage and coverage considering the species with *in-silico* amplicon similarity values ≥97% of the primer pairs evaluated against the oral bacteria genomes. **(A)** Percentages were obtained by the selected bacterial-specific primer pairs. **(B)** Percentages were obtained by the selected bacterial and archaeal primer pairs and the primer pairs used the most in the oral microbiome literature. Among the most commonly used primer pairs in the literature, those marked with an * are bacterial-specific and those with ** target both bacterial and archaea. ASI97, *in-silico* amplicon similarity values ≥97%; F, forward; KP, Klindworth primer; Non-SC, non-coverage of species; OP, oral primer; R, reverse; SC, species coverage; SC-ASI97, species coverage with *in-silico* amplicon similarity values ≥97%; SC-NASI97, species coverage with no *in-silico* amplicon similarity values ≥97%.

**Figure 5:**
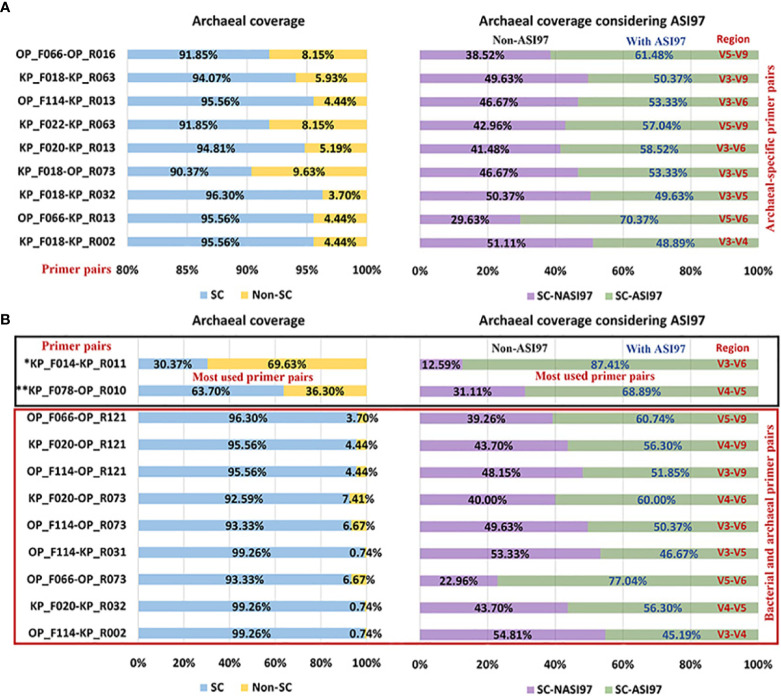
Percentages of coverage and coverage considering the species with *in-silico* amplicon similarity values ≥97% of the primer pairs evaluated against the oral archaea genomes. **(A)** Percentages obtained by the selected archaeal-specific primer pairs. **(B)** Percentages obtained by the selected bacterial and archaeal primer pairs and the primer pairs used the most in the oral-microbiome literature. Among the most commonly used primer pairs in the literature, those marked with an * are archaeal-specific and those with ** target both bacterial and archaea. ASI97, *in-silico* amplicon similarity values ≥97%; F, forward; KP, Klindworth primer; Non-SC, non-coverage of species; OP, oral primer; R, reverse; SC, species coverage; SC-ASI97, species coverage with *in-silico* amplicon similarity values ≥97%; SC-NASI97, species coverage with no *in-silico* amplicon similarity values ≥97%.

**Figures 2**, **3** show the number of species with ASI97 and the number of ASI97 with each primer pair evaluated against bacteria and archaea, respectively; while **Figures 4**, **5** detail the percentages of coverage and coverage considering the presence or absence of ASI97 for both domains. Concerning the bacteria-specific primer pairs, the number of bacterial species with an ASI97 and the total number of ASI97 ranged from 37 and 32 with the most widely used primer, KP_F031-KP_R021 (M; SC-NASI97 = 54.30%), to 120 and 277 with OP_F066-KP_R040 (S; SC-NASI97 = 24.19%), respectively. This latter primer also had the lowest SC-NASI97 value, while OP_F053-KP_R020 detected the highest number of species with no ASI97 (M; SC-NASI97 = 65.05%). In addition, except for OP_F053-KP_R020, all the bacteria-specific primers had a maximum number of ASI97/species above five (range= 4-15 ASI97/species).

Concerning the archaea-specific primer pairs, the number of archaeal species with an ASI97 and the total number of ASI97 ranged from 24 and 96 with the widely used KP_F014-KP_R011 (L; SC-NASI97 = 12.59%) to 89 and 240 with OP_F066-KP_R013 (S; SC-NASI97 = 29.63%), respectively. The former primer detected the lowest number of species without an ASI97, and KP_F018-KP_R002 the highest (S; SC-NASI97 = 51.11%). Moreover, all the archaea-specific primers had a maximum number of ASI97/species ≥10 (range= 10-13 ASI97/species).

Finally, using both the bacterial and archaeal primer pairs, the number of bacterial and archaeal species with an ASI97 and the total number of ASI97 ranged from 84 and 60 and 118 and 126, respectively, with OP_F114-KP_R002 (S; SC-NASI≥97 = 47.31% for bacteria and 54.81% for archaea) to 124 and 95 and 239 and 286, respectively, with OP_F066-OP_R073 (S; SC-NASI≥97 = 31.18% for bacteria and 22.96% for archaea). The latter primer also detected the lowest number of species without an ASI97 and OP_F114-KP_R031 the highest (M; SC-NASI97 = 51.08% for bacteria and 53.33% for archaea) (**Figures 2B–5B**). Most bacterial and archaeal primer combinations had maximum numbers of ASI97/species ≥10 (range= 9-14 ASI/species and 11-14 ASI/species for both domains, respectively).

**Figure 6:**
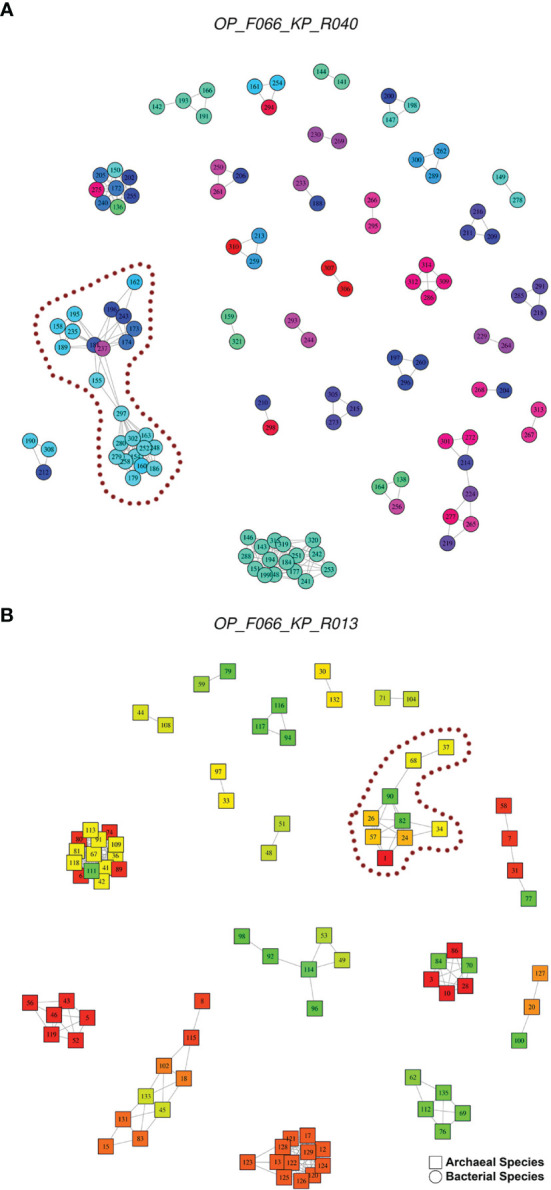
Networks showing the potential OTUs with a ≥97% similarity threshold obtained with the primer pairs. **(A)** OP_F066-KP_R040 for bacteria (120 species with ASI97, 277 ASI97). **(B)** OP_F066-KP_R013 for archaea (89 species with ASI97, 240 ASI97). In the graphs, each node represents an oral species, the color indicates the genus and the number refers to the species identifier, whose assigned species are detailed in the **Supplementary Tables 1**, **2**. Each edge represents the presence of a ≥97% similarity between different species. resulting in clusters of possible OTUs. The graphs were made using the igraph package (version 1.2.6) (Csardi and Nepusz, 2006).

**Figure 7:**
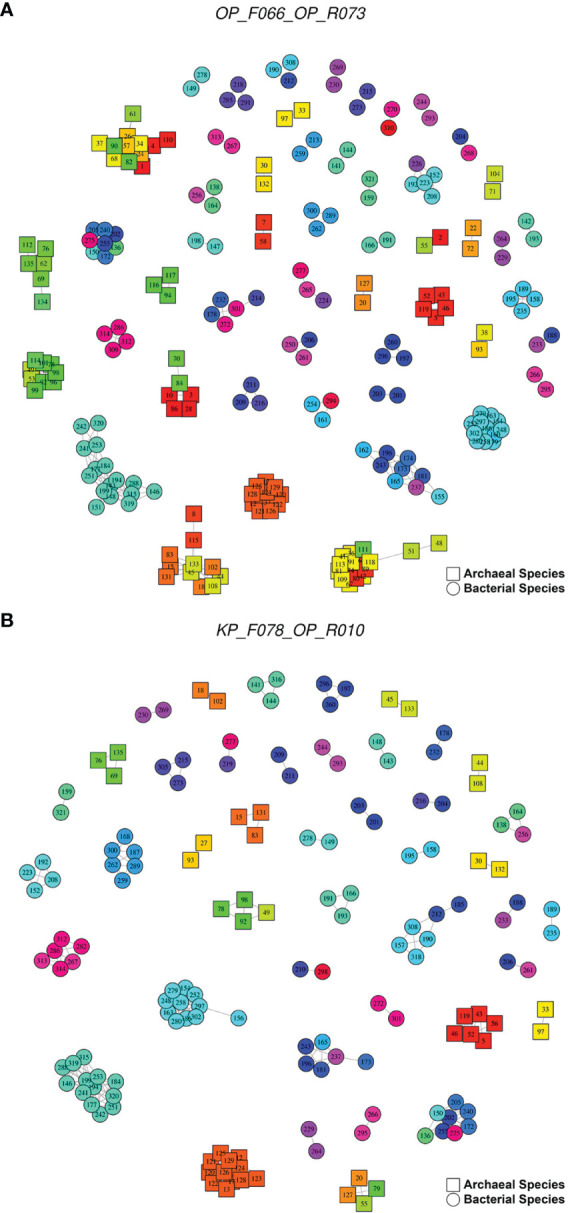
Networks showing the potential OTUs with a ≥97% similarity threshold obtained with the primer pairs for bacteria and archaea. **(A)** OP_F066-OP_R073 (219 species with ASI97, 525 ASI97). **(B)** KP_F078-OP_R010 (primer pair widely used in the oral microbiome literature; 155 species with ASI97, 314 ASI97). In the graphs, each node represents an oral species, the color indicates the genus and the number refers to the species identifier, whose assigned species are detailed in the **Supplementary Tables 1**, **2**. Each edge represents the presence of a ≥97% similarity between different species. resulting in clusters of possible OTUs. The graphs were made using the igraph package (version 1.2.6) (Csardi and Nepusz, 2006).

**Figures 6**, **7** are networks showing the potential clusters (hereinafter referred to as potential OTUs) with a ≥97% similarity threshold obtained with the primer pairs that presented the lowest SC-NASI97 values (F066-KP_R040 for bacteria, OP_F066-KP_R013 for archaea, and OP_F066-OP_R073 for bacteria and archaea), as well as one of the most used primer pairs in the oral microbiome literature, KP_F078-OP_R010. Thus, for example, for the primer pair F066-KP_R040, focusing on the one indicated by a dashed dotted line, 24 bacteria formed a potential OTU, in which 10 genera, five families, and two orders were involved. As can be seen, there were species such as *Ligilactobacillus salivarius* (spp. 162) that presented high similarity only with two others, *Lacticaseibacillus paracasei* (spp. 196) and *Lacticaseibacillus rhamnosus* (spp. 243); while *Staphylococcus cohnii* (spp. 297) presented high similarity with 11 species (among which, *Enterococcus faecalis*, spp. 155; *Staphylococcus aureus*, spp. 163; *Levilactobacillus brevis*, spp. 181; *Lentilactobacillus buchneri*, spp. 237) belonging to four genera, three families and two orders. For the primer pair OP_F066-KP_R013, the potential OTU indicated was formed by nine archaea, involving seven genera and two families. Thus, *Desulfurococcus amylolyticus* (spp. 37) showed high similarity only with *Desulfurococcus mucosus* (spp. 68), while *Thermogladius caldera* had high similarity with five species (*Hyperthermus butylicus*, spp. 24; *Staphylothermus marinus*, spp. 26; *Staphylothermus hellenicus*, spp. 57; *Desulfurococcus mucosus*, spp. 68; *Pyrolobus fumarii*; sp. 82) belonging to four genera and two families.

### Description of the Distinct Pairs of Oral-Bacteria Species and Oral-Archaea Species With *In-Silico* Amplicon Similarity Values ≥97%

**Figure 8:**
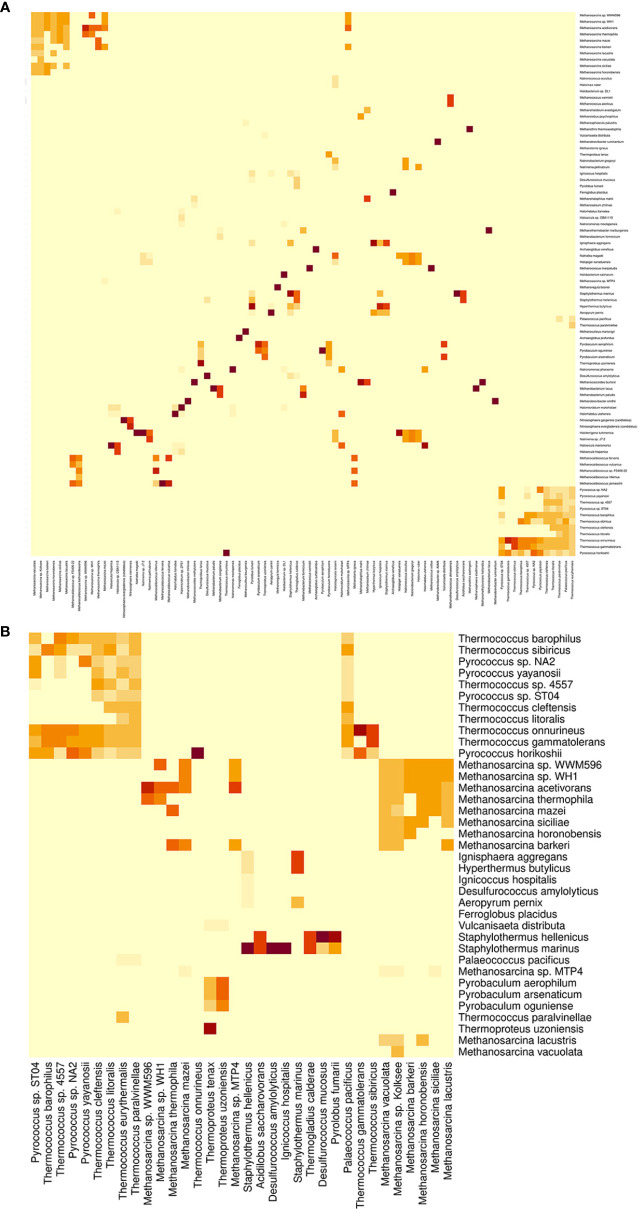
Heat map showing the presence of *in-silico* amplicon similarity values ≥97% between pairs of different bacterial species. **(A)** Global perspective. **(B)** Partial perspective, involving species belonging to genera *Staphylococcus*, *Streptococcus*, *Tannerella*, and *Treponema*. The intensity of the color indicates the frequency, i.e. the number of times a species pair had an *in-silico* amplicon similarity value ≥97% in the different primer pairs evaluated.

**Figure 9:**
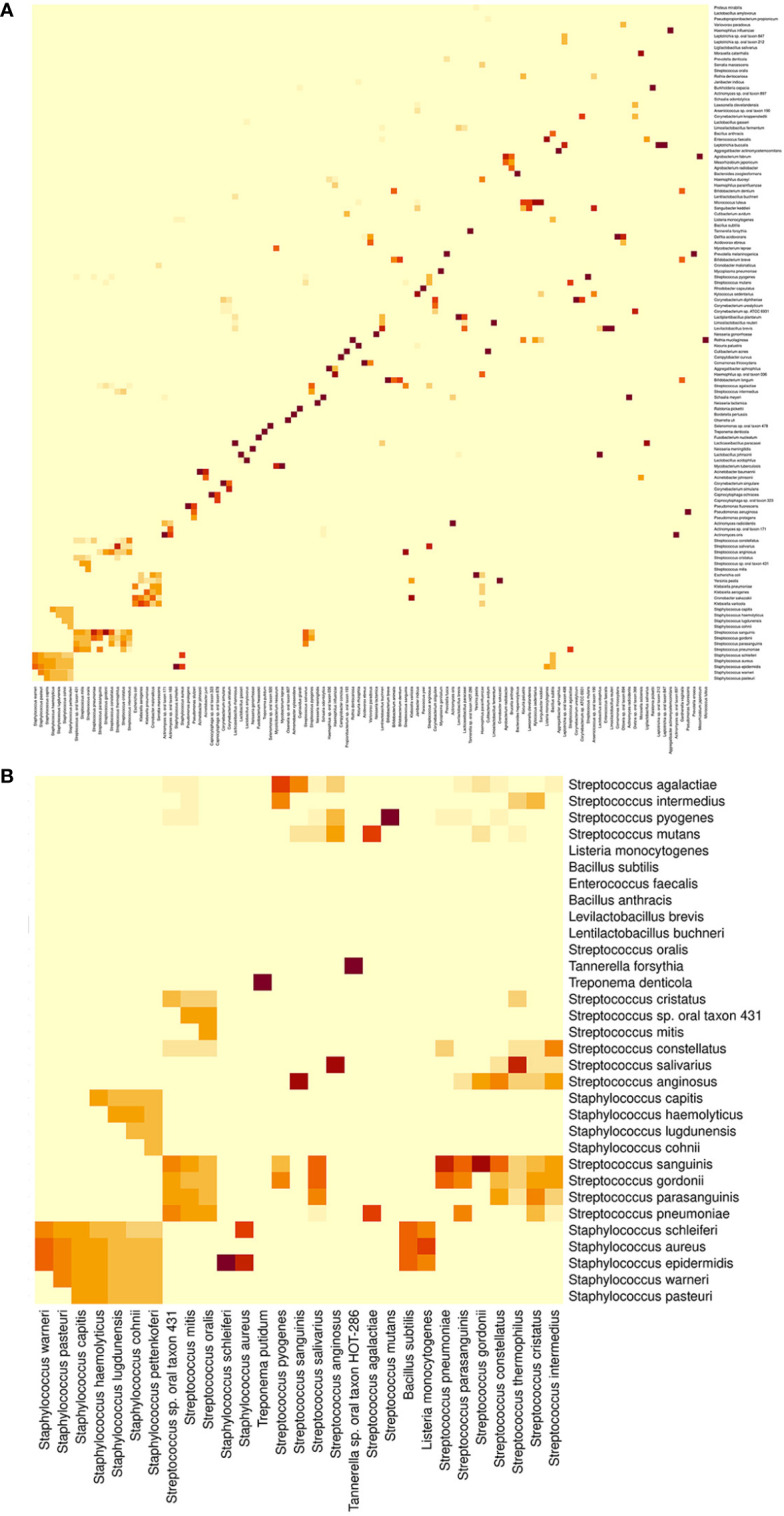
Heat map showing the presence of *in-silico* amplicon similarity values ≥97% between pairs of different archaeal species. **(A)** Global perspective. **(B)** Partial perspective, involving species belonging to genera *Methanosarcina*, *Pyrococcus*, *Staphylothermus*, *Thermococcus*, and *Thermoproteus*. The intensity of the color indicates the frequency, i.e. the number of times a species pair had an *in-silico* amplicon similarity value ≥97% in the different primer pairs evaluated.

One-hundred and forty-nine (80.11%) of the oral-bacteria species and 108 (80.00%) of the oral-archaea species analyzed had an ASI97 with at least one distinct species (**Figures 8**, **9** and **Supplementary Tables 1, 2**). Among them, it is worth mentioning because of their importance in both oral health and disease, *Aggregatibacter actinomycetemcomitans, Campylobacter concisus, Campylobacter curvus, Fusobacterium nucleatum, Rothia dentocariosa, Streptococcus mitis, Streptococcus mutans, Streptococcus oralis, Tannerella forsythia*, and *Treponema denticola*; regarding archaea, *Candidatus Nitrososphaera evergladensis, Halovivax ruber, Methanobrevibacter smithii, Methanococcus maripaludis, Methanosalsum zhiliniae, Methanosarcina barkeri, Methanosarcina mazei, Methanosarcina vacuolata, Methanosphaera stadtmanae* and *Natronococcus occultus.* There were 30 distinct bacterial and 27 distinct archaeal species that could be clustered with a maximum of ≥10 different species when all the analyzed primer pairs were used. Most of these bacterial species belonged to genera *Streptococcus* and *Staphylococcus*; as for archaeal species, to genera *Methanosarcina*, *Thermocococcus*, and *Pyrococcus* (**Supplementary Tables 1, 2**).

Conversely, 37 (19.89%) bacterial species and 27 (20.00%) archaeal species, including *Filifactor alocis*, *Porphyromonas gingivalis*, *Prevotella intermedia*, *Treponema pallidum*, *Veillonella parvula* and *Sulfolobus acidocaldarius* did not have ASI≥97% with other taxa.

All the primers targeting bacteria enabled us to detect 4450 two-on-two relationships between 408 distinct pairs of oral-bacteria species with an ASI97. Eighteen of these different taxa pairs were obtained with the 29 primer pairs analyzed (frequency= 29, parameter defined as the number of times that a pair of species had an ASI97 in the different primer pairs evaluated), which belonged to the genera *Actinomyces*, *Lactobacillus*, *Neisseria*, *Staphylococcus*, and *Streptococcus*. Conversely, 50 species pairs with an ASI97 were detected once by only one primer pair (frequency= 1) (**Supplementary Table 3**). Although the two-on-two relationships mostly involved species from the same genera (3641; 81.82%), 809 relationships (18.18%) were constituted by taxa from different genera. Thus, the combination of species from *Klebsiella* with others from *Cronobacter* occurred most frequently (frequency= 99) followed by *Klebsiella-Serratia*, *Escherichia-Klebsiella*, *Cronobacter-Escherichia* and *Aggregatibacter-Haemophilus* (frequencies= 67-28). For higher taxonomic ranks, 293 (6.58%) two-on-two relationships were between species pairs with an ASI97 belonged to distinct families, with *Enterobacteriaceae* and *Yersiniaceae* being the most frequently detected (frequency= 153); even, there were 26 (0.58%) relationships between species pairs with an ASI97 from different orders, like *Bacillales* and *Lactobacillales* or *Enterobacterales* and *Pasteurellales* (frequencies= 10 and 10, respectively) (**Supplementary Table 4**).

The primers targeting archaea enabled us to detect 3232 two-on-two relationships between 340 different pairs of archaeal species with an ASI97. All primer pairs analyzed identified seven pairs of species (frequency= 20), which belonged to the genera *Methanobrevibacter* and *Methanocaldococcus*. There were 66 species pairs detected only once by only one primer pair (frequency= 1) (**Supplementary Table 5**). Again, most of the two-on-two relationships were between archaeal species from the same genera (2359, 72.99%), but 873 (27.01%) relationships involved taxa pairs with an ASI97 from distinct genera. The combination of species from *Pyrococcus* and *Thermococcus* occurred the most, by far, (frequency= 428), followed by *Palaeococcus* and *Thermococcus* (frequency= 109). For higher taxonomic ranks, 35 (1.08%) relationships were species pairs with an ASI97 from distinct families, such as *Desulfurococcaceae* and *Pyrodictiaceae* (frequency= 27), also belonged to distinct orders (3 relationships; 0.09%), or even classes (1; 0.03%) (**Supplementary Table 6**).

## Discussion

The high degree of similarity between full-length 16S rRNA sequences from distinct species, or even genera, has been reported in the literature (Větrovský and Baldrian, 2013; Schloss, 2021), leading to questions about the reliability of diversity estimates derived from sequence clustering methods based on a given similarity threshold. Using full-length genes and a ≥97% similarity threshold, some authors have detected that around a quarter of constructed OTUs contain sequences from multiple species (Větrovský and Baldrian, 2013; Schloss, 2021) and about a tenth from distinct genera (Větrovský and Baldrian, 2013). These estimates were obviously higher when gene regions were assessed instead of full sequences. Schloss (2021) found that, with a ≥97% similarity threshold and applying the OptiClust algorithm (Westcott and Schloss, 2017), 31.7%, 34.3% and 34.8% of the OTUs assessed had 16S rRNA amplicons from distinct species in the variable regions V3-V4, V4, and V4-V5, respectively (Schloss, 2021). However, these investigations did not focus on taxa inhabiting a specific environment, despite the importance of conducting 16S rRNA gene-based research using habitat-specific databases (Escapa et al., 2020). Consequently, we used primer pairs targeting several variable regions of the 16S rRNA gene (Regueira-Iglesias et al., 2021a) to determine the number of different oral-bacterial and oral-archaeal species with *in-silico* amplicon similarity values ≥97% (ASI97), as well as the potential OTUs that might contain distinct species. Moreover, for the first time in this kind of analysis, we described the specific taxa of the oral ecosystem with highly similar sequence segments, specifying if they belong to different genera or other higher taxonomic ranks.

In the present study, the primer pairs that targeted bacteria had a mean of 91.88 (49.40%) bacterial species with an ASI97 and an average of 153.46 potential OTUs containing distinct species. For those targeting archaea, these numbers were 65.60 (48.59%) and 162.26, respectively. Using the percentage species coverage with no *in-silico* amplicons similarity ≥97% (SC-NASI97) as a selection criterion, the optimum primer pair for detecting oral bacteria was OP_F053-KP_R020. Although the primer used most in the oral microbiome studies, KP_F031-KP_R021 identified slightly fewer species with an ASI97 (37 vs. 58) and number of ASI97 (32 vs. 46); its SC-NASI97 was also lower than that of OP_F053-KP_R020 (54.30% vs. 65.05%). The primer pair producing the best estimates for detecting oral archaea was KP_F018-KP_R002. Again, the widely used primer KP_F014-KP_R011, although it only detected a few species with an ASI97 (24 vs. 60) and number of ASI97 (96 vs. 125), however, also had a considerably lower SC-NASI97 than that of KP_F018-KP_R002 (12.59% vs. 51.11%). Lastly, we recommend the primer OP_F114-KP_R031 for detecting oral bacteria and archaea simultaneously. OP_F114-KP_R002, meanwhile, identified slightly fewer taxa with an ASI≥97% (for bacteria= 84 and for archaea= 60 vs. 85 and 62) and number of ASI97 (118 and 126 vs. 133 and 136) but had a lower SC-NASI≥97% (47.31% and 54.81% vs. 51.08% and 53.33%). In addition, as previously observed (Regueira-Iglesias et al., 2021a; Regueira-Iglesias et al., 2021b), none of the primer combinations that are most commonly employed in sequencing-based studies of the oral microbiome were among the best. Specifically, the species coverage of KP_F078-OP_R010, a primer described by Caporaso (Caporaso et al., 2011), fell from 94.62% for bacteria and 63.70% for archaea (Regueira-Iglesias et al., 2021b) to 34.95% and 31.11% when considering the species with an ASI97, possibly generating as many as 215 and 99 potential bacterial and archaeal OTUs, respectively; that contain different species.

Around 80% of the oral-bacteria and oral-archaea species analyzed had an ASI97 with at least another species. The widely-known bacterial periodontopathogens *Fusobacterium nucleatum* and *Treponema denticola* (Socransky et al., 1998; Teles et al., 2013; Na et al., 2020) had similar *in-silico* amplicons to *Fusobacterium hwasookii* and *Treponema putidum*, respectively, which have also been detected in periodontal lesions (Wyss et al., 2004; Cho et al., 2015). Interestingly, other bacteria with high *in-silico* amplicon similarities had antagonistic roles in oral health and disease. Examples are: the health-associated *Campylobacter concisus* and the initially periodontitis-associated *Campylobacter curvus* (Henne et al., 2014); the health-related *Rothia mucilaginosa* (Zhang et al., 2018) and the decay-abundant *Rothia dentocariosa* (Jiang et al., 2016; Inquimbert et al., 2019); the commensal *Streptococcus mitis, oralis*, and *salivarius*; the caries-associated *Streptococcus mutans* (Teles et al., 2013; Abranches et al., 2018; Lemos et al., 2019); and the periodontal health-related *Tannerella* sp. *oral taxon HOT-286* (Vartoukian et al., 2016; Lenartova et al., 2021) and the periodontitis-related *Tannerella forsythia* (Socransky et al., 1998; Teles et al., 2013; Na et al., 2020). Furthermore, relevant oral-disease associated species, such as *Aggregatibacter actinomycetemcomitans* (Teles et al., 2013; Åberg et al., 2015) and *Rothia dentocariosa* (Jiang et al., 2016; Inquimbert et al., 2019), were among those that had an ASI97 with taxa from distinct genera. Regarding the archaea, we found that four Methanosarcina species found in healthy and periodontitis pockets, namely *barkeri*, *lacustris*, *mazeii*, and *vacuolata* (Deng et al., 2017), were highly similar. Moreover, *Halovivax ruber*, *Methanotorris igneus*, *Methanosalsum zhilinae*, and *Natronococcus occultus*, which are reported to be among the 10 most abundant species in both healthy and periodontitis subjects (Deng et al., 2017), had an ASI97 with several taxa from distinct genera.

Schloss (2021) has recently stated that the risks of artificially splitting a genome into multiple amplicon sequence variants (ASVs) are greater than those of clustering ASVs from different species into the same OTU when using broad distance thresholds. However, considering the results obtained in the present study, our opinion is that the latter approach should be avoided in the analysis of the oral microbiome if the aim is to associate species with specific clinical conditions. *In-silico* amplicons from species traditionally associated with contrary health conditions, like those described above, can be grouped with a ≥97% similarity threshold. This would result in both an overabundance of the single species representing the OTU and an underestimation of the diversity of the community, with other species within the OTU overlooked. Consequently, it would be better to use the lowest possible level of resolution, i.e., the variant level (Callahan et al., 2017), and databases specifically designed for taxonomic identifications of taxa at this level (Escapa et al., 2020).

It has been demonstrated that distinct OTU clustering approaches, or even the same method, can yield uneven results for the same dataset (He et al., 2015; Westcott and Schloss, 2015; Wei et al., 2021). Therefore, we decided to analyze the 97% similarity relationships between oral species, without considering the influence of any clustering algorithm. Consequently, the results presented here are an approximation of the different oral species that could be grouped in potential OTUs.

The main limitation of our study is that we have only considered one, randomly selected, of all possible *in-silico* amplicons with ASI97 between two different species to establish the existence of a close relationship between the two. Another consideration is that we were only able to evaluate 25% of the oral microorganism genomes listed on the eHOMD website, as the remainder were not fully sequenced. This absence of complete genomes reduced the number of species investigated to 35% of those set out on the site. Although the analysis could have been performed on annotations of the 16S rRNA gene sequences from oral microbes, we preferred to use complete genomes, thereby ensuring the high quality of the sequences reviewed. The reasons why we adopted this approach were: 1) Edgar (2018) estimated that the taxonomy annotation error rate of the Ribosomal Database Project (RDP) (Cole et al., 2014) database is ∼10%; on the other hand, he found 249,490 identical sequences with conflicting annotations in SILVA v128 (Quast et al., 2013) and Greengenes v13.5 (DeSantis et al., 2006) at ranks up to phylum (7,804 conflicts), indicating that the annotation error rate in these databases is ∼17%; 2) we have verified in previous research that a very high percentage of 16S rRNA gene annotations present a loss of information of up to 60-70 nucleotides in regions 1 and 9 of the sequences, which invalidates their use (Regueira-Iglesias et al., 2021a); 3) most of the complete genomes evaluated here are isolates that were sequenced with Sanger technology or with second-generation technology (shorter sequences than Sanger). In both cases, contig scaffolding algorithms were used to construct the complete genomes from the sequences with a minimum coverage of 8x for Sanger sequences and 30x in the case of second-generation technologies (Schatz et al., 2010). In these types of assemblies, positions within the genome that did not have high coverage included non-specific nucleotides. In the present study, we discarded genomes that included more than 20 consecutive unspecific positions; 4) in addition, many genomes were downloaded from the NCBI RefSef database (O'Leary et al., 2016), where the annotations of the complete genomes were manually curated or re-annotated concerning the information provided by the original author, including their taxonomic hierarchy. Thus, our results highlight only part of a much more extensive problem.

In conclusion, the tested primer pairs targeting bacteria and/or archaea detected an average of more than 150 potential OTUs that might contain different species, when ≥97% similarity threshold was used. According to the SC-NASI97 parameter, the best primer pairs were: OP_F053-KP_R020 for bacteria (variable region V1-V3; primer pair position for *Escherichia coli* J01859.1: 9-356); KP_F018-KP_R002 for archaea (V4; undefined-532); and OP_F114-KP_R031 for both (V3-V5; 340-801). Around 80% of the oral-bacteria and oral-archaea species analyzed had an ASI97 with at least one other species. These very similar species play different roles in the oral microbiota and belong to bacterial genera such as *Campylobacter*, *Rothia*, *Streptococcus* and *Tannerella*, and archaeal genera such as *Halovivax*, *Methanosarcina* and *Methanosalsum*. Moreover, ~20% and ~30% of these two-by-two similarity relationships were established between species from different bacterial and archaeal genera, respectively. Even taxa from distinct families, orders, and classes could be grouped in the same potential OTU. Consequently, regardless of the primer pair used, sequence-clustering with ≥97% similarity provides an inaccurate description of oral-bacterial and oral-archaeal species, which can greatly affect microbial diversity parameters. As a result, OTU clustering conditions the credibility of associations between some oral species and certain health and disease conditions. This significantly limits the comparability of the microbial diversity findings reported in oral microbiome literature.

## Data Availability Statement

The datasets presented in this study can be found in online repositories. The names of the repository/repositories can be found in the article. The scripts developed to carry out the present study were deposited in the public repository of the Centro Singular de Investigación en Tecnoloxías Intelixentes and Departamento de Electrónica e Computación, Universidade de Santiago de Compostela, Spain (https://gitlab.citius.usc.es/lara.vazquez/oral16sampliconsimilarity).

## Author Contributions

CB-C, BM-B, MC, and IT contributed to the conception and design of the study, and critically revised manuscript. AR-I, LV-G, TB-P, and VA contributed to acquisition, analysis, and interpretation, and drafted the manuscript. All the authors gave final approval and agree to be accountable for all aspects of the work in ensuring that questions relating to the accuracy or integrity of any part of the work are appropriately investigated and resolved.

## Funding

This investigation was supported by the Instituto de Salud Carlos III (General Division of Evaluation and Research Promotion, Madrid, Spain) and co-financed by the FEDER (European Regional Development Fund, ERDF) (“A way of making Europe”) under grant ISCIII/PI17/01722; the Consellería de Cultura, Educación e Ordenación Universitaria de la Xunta de Galicia (accreditation 2019-2022 ED431G-2019/04, group with growth potential ED431B 2020-2022 GPC2020/27; A. Regueira-Iglesias support ED481A-2017/233) and the ERDF, which acknowledges the CiTIUS-Research Center in Intelligent Technologies of the Santiago de Compostela University as a Research Center of the Galician University System. The funders had no role in study design, data collection and analysis, decision to publish, or preparation of the manuscript.

## Conflict of Interest

The authors declare that the research was conducted in the absence of any commercial or financial relationships that could be construed as a potential conflict of interest.

## Publisher’s Note

All claims expressed in this article are solely those of the authors and do not necessarily represent those of their affiliated organizations, or those of the publisher, the editors and the reviewers. Any product that may be evaluated in this article, or claim that may be made by its manufacturer, is not guaranteed or endorsed by the publisher.
