# Supplementary material for: In-Silico Detection of Oral Prokaryotic Species With Highly Similar 16S rRNA Sequence Segments Using Different Primer Pairs
Source: Front Cell Infect Microbiol. 2022 Feb 9;11:770668. doi: 10.3389/fcimb.2021.770668 (PMC8863748; doi:10.3389/fcimb.2021.770668)
Supplement: Supplementary file 1 [file Table_1.docx]

**Supplementary Material**

Supplementary Table 1. Taxonomy of oral-bacteria species with *in-silico* amplicon similarity values ≥97% with at least one different taxon, their mean number of intragenomic 16S rRNA genes, and taxa with a maximum number of *in-silico* amplicon similarity values ≥97%/species ≥10.

Supplementary Table 2. Taxonomy of oral-archaea species with *in-silico* amplicon similarity values ≥97% with at least one different taxon, their mean number of intragenomic 16S rRNA genes, and taxa with a maximum number of *in-silico* amplicon similarity values ≥97%/species ≥10.

Supplementary Table 3. Pairs of bacterial species with *in-silico* amplicon similarity values ≥97% using the analyzed primer pairs.

Supplementary Table 4. Pairs of different bacterial genera, families, and orders with *in-silico* amplicon similarity values ≥97%.

Supplementary Table 5. Pairs of archaeal species with *in-silico* amplicon similarity values ≥97% using the analyzed primer pairs.

Supplementary Table 6. Pairs of different archaeal genera, families, orders, and classes with *in-silico* amplicon similarity values ≥97%.

Supplementary Table 1. Taxonomy of oral-bacteria species with *in-silico* amplicon similarity values ≥97% with at least one different taxon, their mean number of intragenomic 16S rRNA genes, and taxa with a maximum number of *in-silico* amplicon similarity values ≥97%/species ≥10.

| **ID** | **No. genes**  **(mean)** | **Phylum** | **Class** | **Order** | **Family** | **Genus** | **Species** | **Max. No. ASI97/sp. ≥10** |
| --- | --- | --- | --- | --- | --- | --- | --- | --- |
| SP00136 | 7.00 | Proteobacteria | Gammaproteobacteria | Enterobacterales | Enterobacteriaceae | Escherichia | coli | No |
| SP00138 | 1.00 | Actinobacteria | Actinomycetia | Corynebacteriales | Mycobacteriaceae | Mycobacterium | tuberculosis | No |
| SP00141 | 4.00 | Proteobacteria | Betaproteobacteria | Neisseriales | Neisseriaceae | Neisseria | meningitidis | No |
| SP00142 | 4.00 | Proteobacteria | Gammaproteobacteria | Pseudomonadales | Pseudomonadaceae | Pseudomonas | aeruginosa | No |
| SP00143 | 6.00 | Firmicutes | Bacilli | Lactobacillales | Streptococcaceae | Streptococcus | pyogenes | Yes |
| SP00144 | 4.00 | Proteobacteria | Betaproteobacteria | Neisseriales | Neisseriaceae | Neisseria | gonorrhoeae | No |
| SP00146 | 4.00 | Firmicutes | Bacilli | Lactobacillales | Streptococcaceae | Streptococcus | pneumoniae | Yes |
| SP00147 | 4.00 | Proteobacteria | Alphaproteobacteria | Hyphomicrobiales | Rhizobiaceae | Agrobacterium | fabrum | No |
| SP00148 | 7.00 | Firmicutes | Bacilli | Lactobacillales | Streptococcaceae | Streptococcus | agalactiae | Yes |
| SP00149 | 4.00 | Fusobacteria | Fusobacteriia | Fusobacteriales | Fusobacteriaceae | Fusobacterium | nucleatum | No |
| SP00150 | 7.00 | Proteobacteria | Gammaproteobacteria | Enterobacterales | Yersiniaceae | Yersinia | pestis | No |
| SP00151 | 5.00 | Firmicutes | Bacilli | Lactobacillales | Streptococcaceae | Streptococcus | mutans | Yes |
| SP00152 | 4.00 | Actinobacteria | Actinomycetia | Bifidobacteriales | Bifidobacteriaceae | Bifidobacterium | longum | No |
| SP00154 | 6.00 | Firmicutes | Bacilli | Bacillales | Staphylococcaceae | Staphylococcus | epidermidis | Yes |
| SP00155 | 4.00 | Firmicutes | Bacilli | Lactobacillales | Enterococcaceae | Enterococcus | faecalis | No |
| SP00156 | 11.00 | Firmicutes | Bacilli | Bacillales | Bacillaceae | Bacillus | anthracis | No |
| SP00157 | 6.00 | Proteobacteria | Gammaproteobacteria | Pasteurellales | Pasteurellaceae | Haemophilus | ducreyi | No |
| SP00158 | 5.00 | Firmicutes | Bacilli | Lactobacillales | Lactobacillaceae | Lactobacillus | johnsonii | No |
| SP00159 | 2.00 | Spirochaetes | Spirochaetia | Spirochaetales | Spirochaetaceae | Treponema | denticola | No |
| SP00160 | 6.00 | Firmicutes | Bacilli | Bacillales | Listeriaceae | Listeria | monocytogenes | Yes |
| SP00161 | 3.00 | Actinobacteria | Actinomycetia | Propionibacteriales | Propionibacteriaceae | Cutibacterium | acnes | No |
| SP00162 | 7.00 | Firmicutes | Bacilli | Lactobacillales | Lactobacillaceae | Ligilactobacillus | salivarius | No |
| SP00163 | 5.00 | Firmicutes | Bacilli | Bacillales | Staphylococcaceae | Staphylococcus | aureus | Yes |
| SP00164 | 1.00 | Actinobacteria | Actinomycetia | Corynebacteriales | Mycobacteriaceae | Mycobacterium | leprae | No |
| SP00165 | 5.00 | Firmicutes | Bacilli | Lactobacillales | Lactobacillaceae | Lactiplantibacillus | plantarum | No |
| SP00166 | 6.00 | Proteobacteria | Gammaproteobacteria | Pseudomonadales | Pseudomonadaceae | Pseudomonas | fluorescens | No |
| SP00168 | 3.00 | Actinobacteria | Actinomycetia | Corynebacteriales | Corynebacteriaceae | Corynebacterium | urealyticum | No |
| SP00169 | 7.00 | Proteobacteria | Gammaproteobacteria | Enterobacterales | Morganellaceae | Proteus | mirabilis | No |
| SP00170 | 2.00 | Proteobacteria | Alphaproteobacteria | Hyphomicrobiales | Phyllobacteriaceae | Mesorhizobium | japonicum | No |
| SP00172 | 8.00 | Proteobacteria | Gammaproteobacteria | Enterobacterales | Enterobacteriaceae | Klebsiella | pneumoniae | No |
| SP00173 | 6.00 | Firmicutes | Bacilli | Lactobacillales | Lactobacillaceae | Limosilactobacillus | reuteri | No |
| SP00174 | 5.00 | Firmicutes | Bacilli | Lactobacillales | Lactobacillaceae | Limosilactobacillus | fermentum | No |
| SP00177 | 4.00 | Firmicutes | Bacilli | Lactobacillales | Streptococcaceae | Streptococcus | intermedius | Yes |
| SP00178 | 3.00 | Actinobacteria | Actinomycetia | Micrococcales | Micrococcaceae | Rothia | mucilaginosa | No |
| SP00179 | 9.00 | Firmicutes | Bacilli | Bacillales | Bacillaceae | Bacillus | subtilis | Yes |
| SP00181 | 5.00 | Firmicutes | Bacilli | Lactobacillales | Lactobacillaceae | Levilactobacillus | brevis | Yes |
| SP00182 | 1.00 | Tenericutes | Mollicutes | Mycoplasmatales | Mycoplasmataceae | Mycoplasma | pneumoniae | No |
| SP00184 | 4.00 | Firmicutes | Bacilli | Lactobacillales | Streptococcaceae | Streptococcus | anginosus | Yes |
| SP00185 | 6.00 | Proteobacteria | Gammaproteobacteria | Pasteurellales | Pasteurellaceae | Aggregatibacter | actinomycetemcomitans | No |
| SP00186 | 5.00 | Firmicutes | Bacilli | Bacillales | Staphylococcaceae | Staphylococcus | schleiferi | Yes |
| SP00187 | 5.00 | Actinobacteria | Actinomycetia | Corynebacteriales | Corynebacteriaceae | Corynebacterium | diphtheriae | No |
| SP00188 | 3.00 | Proteobacteria | Betaproteobacteria | Burkholderiales | Alcaligenaceae | Bordetella | pertussis | No |
| SP00189 | 4.00 | Firmicutes | Bacilli | Lactobacillales | Lactobacillaceae | Lactobacillus | acidophilus | No |
| SP00190 | 6.00 | Proteobacteria | Gammaproteobacteria | Pasteurellales | Pasteurellaceae | Haemophilus | influenzae | No |
| SP00191 | 5.00 | Proteobacteria | Gammaproteobacteria | Pseudomonadales | Pseudomonadaceae | Pseudomonas | protegens | No |
| SP00192 | 3.00 | Actinobacteria | Actinomycetia | Bifidobacteriales | Bifidobacteriaceae | Bifidobacterium | breve | No |
| SP00193 | 4.00 | Proteobacteria | Gammaproteobacteria | Pseudomonadales | Pseudomonadaceae | Pseudomonas | stutzeri | No |
| SP00194 | 4.00 | Firmicutes | Bacilli | Lactobacillales | Streptococcaceae | Streptococcus | sanguinis | Yes |
| SP00195 | 6.00 | Firmicutes | Bacilli | Lactobacillales | Lactobacillaceae | Lactobacillus | gasseri | No |
| SP00196 | 5.00 | Firmicutes | Bacilli | Lactobacillales | Lactobacillaceae | Lacticaseibacillus | paracasei | No |
| SP00197 | 6.00 | Proteobacteria | Gammaproteobacteria | Pseudomonadales | Moraxellaceae | Acinetobacter | baumannii | No |
| SP00198 | 3.00 | Proteobacteria | Alphaproteobacteria | Hyphomicrobiales | Rhizobiaceae | Agrobacterium | radiobacter | No |
| SP00199 | 4.00 | Firmicutes | Bacilli | Lactobacillales | Streptococcaceae | Streptococcus | gordonii | Yes |
| SP00200 | 4.00 | Proteobacteria | Alphaproteobacteria | Hyphomicrobiales | Brucellaceae | Brucella | anthropi | No |
| SP00201 | 3.00 | Proteobacteria | Epsilonproteobacteria | Campylobacterales | Campylobacteraceae | Campylobacter | curvus | No |
| SP00202 | 7.00 | Proteobacteria | Gammaproteobacteria | Enterobacterales | Enterobacteriaceae | Cronobacter | sakazakii | No |
| SP00203 | 3.00 | Proteobacteria | Epsilonproteobacteria | Campylobacterales | Campylobacteraceae | Campylobacter | concisus | No |
| SP00204 | 5.00 | Proteobacteria | Betaproteobacteria | Burkholderiales | Comamonadaceae | Delftia | acidovorans | No |
| SP00205 | 8.00 | Proteobacteria | Gammaproteobacteria | Enterobacterales | Enterobacteriaceae | Klebsiella | variicola | No |
| SP00206 | 4.00 | Proteobacteria | Betaproteobacteria | Burkholderiales | Burkholderiaceae | Ralstonia | pickettii | No |
| SP00208 | 4.00 | Actinobacteria | Actinomycetia | Bifidobacteriales | Bifidobacteriaceae | Bifidobacterium | animalis | No |
| SP00209 | 3.00 | Proteobacteria | Betaproteobacteria | Burkholderiales | Comamonadaceae | Comamonas | thiooxydans | No |
| SP00210 | 4.00 | Proteobacteria | Alphaproteobacteria | Rhodobacterales | Rhodobacteraceae | Rhodobacter | capsulatus | No |
| SP00211 | 3.00 | Proteobacteria | Betaproteobacteria | Burkholderiales | Comamonadaceae | Acidovorax | ebreus | No |
| SP00212 | 6.00 | Proteobacteria | Gammaproteobacteria | Pasteurellales | Pasteurellaceae | Aggregatibacter | aphrophilus | No |
| SP00213 | 3.00 | Actinobacteria | Actinomycetia | Corynebacteriales | Corynebacteriaceae | Corynebacterium | kroppenstedtii | No |
| SP00214 | 1.00 | Actinobacteria | Actinomycetia | Micrococcales | Micrococcaceae | Micrococcus | luteus | No |
| SP00215 | 4.00 | Bacteroidetes | Flavobacteriia | Flavobacteriales | Flavobacteriaceae | Capnocytophaga | ochracea | No |
| SP00216 | 2.00 | Proteobacteria | Betaproteobacteria | Burkholderiales | Comamonadaceae | Variovorax | paradoxus | No |
| SP00218 | 5.00 | Fusobacteria | Fusobacteriia | Fusobacteriales | Leptotrichiaceae | Leptotrichia | buccalis | No |
| SP00219 | 2.00 | Actinobacteria | Actinomycetia | Micrococcales | Kytococcaceae | Kytococcus | sedentarius | No |
| SP00223 | 4.00 | Actinobacteria | Actinomycetia | Bifidobacteriales | Bifidobacteriaceae | Bifidobacterium | dentium | No |
| SP00224 | 4.00 | Actinobacteria | Actinomycetia | Micrococcales | Sanguibacteraceae | Sanguibacter | keddieii | No |
| SP00226 | 2.00 | Actinobacteria | Actinomycetia | Bifidobacteriales | Bifidobacteriaceae | Gardnerella | vaginalis | No |
| SP00227 | 4.00 | Proteobacteria | Gammaproteobacteria | Pseudomonadales | Moraxellaceae | Moraxella | catarrhalis | No |
| SP00229 | 1.00 | Actinobacteria | Coriobacteriia | Coriobacteriales | Atopobiaceae | Olsenella | uli | No |
| SP00230 | 4.00 | Bacteroidetes | Bacteroidia | Bacteroidales | Prevotellaceae | Prevotella | melaninogenica | No |
| SP00232 | 3.00 | Actinobacteria | Actinomycetia | Micrococcales | Micrococcaceae | Rothia | dentocariosa | No |
| SP00233 | 3.00 | Proteobacteria | Betaproteobacteria | Burkholderiales | Alcaligenaceae | Achromobacter | xylosoxidans | No |
| SP00235 | 4.00 | Firmicutes | Bacilli | Lactobacillales | Lactobacillaceae | Lactobacillus | amylovorus | No |
| SP00236 | 4.00 | Bacteroidetes | Bacteroidia | Bacteroidales | Prevotellaceae | Prevotella | denticola | No |
| SP00237 | 5.00 | Firmicutes | Bacilli | Lactobacillales | Lactobacillaceae | Lentilactobacillus | buchneri | Yes |
| SP00239 | 2.00 | Actinobacteria | Actinomycetia | Propionibacteriales | Propionibacteriaceae | Pseudopropionibacterium | propionicum | No |
| SP00240 | 8.00 | Proteobacteria | Gammaproteobacteria | Enterobacterales | Enterobacteriaceae | Klebsiella | aerogenes | No |
| SP00241 | 4.00 | Firmicutes | Bacilli | Lactobacillales | Streptococcaceae | Streptococcus | parasanguinis | Yes |
| SP00242 | 6.00 | Firmicutes | Bacilli | Lactobacillales | Streptococcaceae | Streptococcus | salivarius | Yes |
| SP00243 | 5.00 | Firmicutes | Bacilli | Lactobacillales | Lactobacillaceae | Lacticaseibacillus | rhamnosus | No |
| SP00244 | 2.00 | Bacteroidetes | Bacteroidia | Bacteroidales | Tannerellaceae | Tannerella | forsythia | No |
| SP00248 | 5.00 | Firmicutes | Bacilli | Bacillales | Staphylococcaceae | Staphylococcus | warneri | Yes |
| SP00249 | 1.00 | Tenericutes | Mollicutes | Mycoplasmatales | Mycoplasmataceae | Mycoplasma | genitalium | No |
| SP00250 | 2.00 | Proteobacteria | Betaproteobacteria | Burkholderiales | Burkholderiaceae | Burkholderia | cepacia | No |
| SP00251 | 4.00 | Firmicutes | Bacilli | Lactobacillales | Streptococcaceae | Streptococcus | constellatus | Yes |
| SP00252 | 5.00 | Firmicutes | Bacilli | Bacillales | Staphylococcaceae | Staphylococcus | pasteuri | Yes |
| SP00253 | 4.00 | Firmicutes | Bacilli | Lactobacillales | Streptococcaceae | Streptococcus | cristatus | Yes |
| SP00254 | 3.00 | Actinobacteria | Actinomycetia | Propionibacteriales | Propionibacteriaceae | Cutibacterium | avidum | No |
| SP00255 | 7.00 | Proteobacteria | Gammaproteobacteria | Enterobacterales | Enterobacteriaceae | Cronobacter | malonaticus | No |
| SP00256 | 2.00 | Actinobacteria | Actinomycetia | Corynebacteriales | Mycobacteriaceae | Mycolicibacterium | neoaurum | No |
| SP00258 | 6.00 | Firmicutes | Bacilli | Bacillales | Staphylococcaceae | Staphylococcus | capitis | Yes |
| SP00259 | 5.00 | Actinobacteria | Actinomycetia | Corynebacteriales | Corynebacteriaceae | Corynebacterium | sp. ATCC 6931 | No |
| SP00260 | 7.00 | Proteobacteria | Gammaproteobacteria | Pseudomonadales | Moraxellaceae | Acinetobacter | johnsonii | No |
| SP00261 | 4.00 | Proteobacteria | Betaproteobacteria | Burkholderiales | Burkholderiaceae | Cupriavidus | gilardii | No |
| SP00262 | 4.00 | Actinobacteria | Actinomycetia | Corynebacteriales | Corynebacteriaceae | Corynebacterium | singulare | No |
| SP00264 | 2.00 | Actinobacteria | Coriobacteriia | Coriobacteriales | Atopobiaceae | Olsenella | sp. oral taxon 807 | No |
| SP00265 | 2.00 | Actinobacteria | Actinomycetia | Micrococcales | Intrasporangiaceae | Arsenicicoccus | sp. oral taxon 190 | No |
| SP00266 | 4.00 | Firmicutes | Negativicutes | Selenomonadales | Selenomonadaceae | Selenomonas | sp. oral taxon 478 | No |
| SP00267 | 3.00 | Actinobacteria | Actinomycetia | Actinomycetales | Actinomycetaceae | Schaalia | meyeri | No |
| SP00268 | 3.00 | Proteobacteria | Betaproteobacteria | Burkholderiales | Comamonadaceae | Ottowia | sp. oral taxon 894 | No |
| SP00269 | 3.00 | Bacteroidetes | Bacteroidia | Bacteroidales | Prevotellaceae | Prevotella | fusca | No |
| SP00270 | 2.00 | Actinobacteria | Actinomycetia | Corynebacteriales | Lawsonellaceae | Lawsonella | clevelandensis | No |
| SP00271 | 4.00 | Fusobacteria | Fusobacteriia | Fusobacteriales | Leptotrichiaceae | Leptotrichia | sp. oral taxon 212 | No |
| SP00272 | 3.00 | Actinobacteria | Actinomycetia | Micrococcales | Micrococcaceae | Kocuria | palustris | No |
| SP00273 | 4.00 | Bacteroidetes | Flavobacteriia | Flavobacteriales | Flavobacteriaceae | Capnocytophaga | sp. oral taxon 323 | No |
| SP00275 | 7.00 | Proteobacteria | Gammaproteobacteria | Enterobacterales | Yersiniaceae | Serratia | marcescens | No |
| SP00276 | 3.00 | Bacteroidetes | Bacteroidia | Bacteroidales | Prevotellaceae | Prevotella | enoeca | No |
| SP00277 | 2.00 | Actinobacteria | Actinomycetia | Micrococcales | Intrasporangiaceae | Janibacter | indicus | No |
| SP00278 | 5.00 | Fusobacteria | Fusobacteriia | Fusobacteriales | Fusobacteriaceae | Fusobacterium | hwasookii | No |
| SP00279 | 6.00 | Firmicutes | Bacilli | Bacillales | Staphylococcaceae | Staphylococcus | haemolyticus | Yes |
| SP00280 | 6.00 | Firmicutes | Bacilli | Bacillales | Staphylococcaceae | Staphylococcus | lugdunensis | Yes |
| SP00282 | 3.00 | Actinobacteria | Actinomycetia | Actinomycetales | Actinomycetaceae | Actinomyces | radicidentis | No |
| SP00285 | 5.00 | Fusobacteria | Fusobacteriia | Fusobacteriales | Leptotrichiaceae | Leptotrichia | sp. oral taxon 847 | No |
| SP00286 | 3.00 | Actinobacteria | Actinomycetia | Actinomycetales | Actinomycetaceae | Actinomyces | oris | No |
| SP00287 | 4.00 | Proteobacteria | Gammaproteobacteria | Pseudomonadales | Moraxellaceae | Moraxella | osloensis | No |
| SP00288 | 4.00 | Firmicutes | Bacilli | Lactobacillales | Streptococcaceae | Streptococcus | sp. oral taxon 431 | Yes |
| SP00289 | 4.00 | Actinobacteria | Actinomycetia | Corynebacteriales | Corynebacteriaceae | Corynebacterium | simulans | No |
| SP00291 | 5.00 | Fusobacteria | Fusobacteriia | Fusobacteriales | Leptotrichiaceae | Leptotrichia | sp. oral taxon 498 | No |
| SP00293 | 2.00 | Bacteroidetes | Bacteroidia | Bacteroidales | Tannerellaceae | Tannerella | sp. oral taxon HOT-286 | No |
| SP00294 | 3.00 | Actinobacteria | Actinomycetia | Propionibacteriales | Propionibacteriaceae | Propionibacterium | sp. oral taxon 193 | No |
| SP00295 | 4.00 | Firmicutes | Negativicutes | Selenomonadales | Selenomonadaceae | Selenomonas | sp. oral taxon 920 | No |
| SP00296 | 6.00 | Proteobacteria | Gammaproteobacteria | Pseudomonadales | Moraxellaceae | Acinetobacter | junii | No |
| SP00297 | 6.00 | Firmicutes | Bacilli | Bacillales | Staphylococcaceae | Staphylococcus | cohnii | Yes |
| SP00298 | 3.00 | Proteobacteria | Alphaproteobacteria | Rhodobacterales | Rhodobacteraceae | Paracoccus | yeei | No |
| SP00300 | 4.00 | Actinobacteria | Actinomycetia | Corynebacteriales | Corynebacteriaceae | Corynebacterium | striatum | No |
| SP00301 | 3.00 | Actinobacteria | Actinomycetia | Micrococcales | Micrococcaceae | Kocuria | rhizophila | No |
| SP00302 | 6.00 | Firmicutes | Bacilli | Bacillales | Staphylococcaceae | Staphylococcus | pettenkoferi | Yes |
| SP00305 | 4.00 | Bacteroidetes | Flavobacteriia | Flavobacteriales | Flavobacteriaceae | Capnocytophaga | sp. oral taxon 878 | No |
| SP00306 | 4.00 | Bacteroidetes | Bacteroidia | Bacteroidales | Bacteroidaceae | Bacteroides | zoogleoformans | No |
| SP00307 | 4.00 | Bacteroidetes | Bacteroidia | Bacteroidales | Bacteroidaceae | Bacteroides | heparinolyticus | No |
| SP00308 | 6.00 | Proteobacteria | Gammaproteobacteria | Pasteurellales | Pasteurellaceae | Haemophilus | sp. oral taxon 036 | No |
| SP00309 | 3.00 | Actinobacteria | Actinomycetia | Actinomycetales | Actinomycetaceae | Actinomyces | sp. oral taxon 897 | No |
| SP00310 | 4.00 | Actinobacteria | Actinomycetia | Corynebacteriales | Dietziaceae | Dietzia | sp. oral taxon 368 | No |
| SP00312 | 3.00 | Actinobacteria | Actinomycetia | Actinomycetales | Actinomycetaceae | Actinomyces | sp. oral taxon 171 | No |
| SP00313 | 3.00 | Actinobacteria | Actinomycetia | Actinomycetales | Actinomycetaceae | Schaalia | odontolytica | No |
| SP00314 | 3.00 | Actinobacteria | Actinomycetia | Actinomycetales | Actinomycetaceae | Actinomyces | sp. oral taxon 169 | No |
| SP00315 | 4.00 | Firmicutes | Bacilli | Lactobacillales | Streptococcaceae | Streptococcus | mitis | Yes |
| SP00316 | 4.00 | Proteobacteria | Betaproteobacteria | Neisseriales | Neisseriaceae | Neisseria | lactamica | No |
| SP00318 | 6.00 | Proteobacteria | Gammaproteobacteria | Pasteurellales | Pasteurellaceae | Haemophilus | parainfluenzae | No |
| SP00319 | 4.00 | Firmicutes | Bacilli | Lactobacillales | Streptococcaceae | Streptococcus | oralis | Yes |
| SP00320 | 6.00 | Firmicutes | Bacilli | Lactobacillales | Streptococcaceae | Streptococcus | thermophilus | Yes |
| SP00321 | 2.00 | Spirochaetes | Spirochaetia | Spirochaetales | Spirochaetaceae | Treponema | putidum | No |

The Table illustrates the bacterial species which had *in-silico* amplicon similarity values ≥97% with at least one different taxon using the bacterial-specific and the bacterial and archaeal primer pairs analyzed in the present study, signalling those with a maximum number of *in-silico* amplicon similarity values ≥97%/species ≥10. The mean number of intragenomic 16S rRNA genes of these species was calculated in a previous investigation of our group (Regueira-Iglesias et al. 2021.b). ASI97= *in silico* amplicon similarity values ≥97%; ID= species identifier; Max.= maximum; No.= number; sp.= species.

Supplementary Table 2. Taxonomy of oral-archaea species with *in-silico* amplicon similarity values ≥97% with at least one different taxon, their mean number of intragenomic 16S rRNA genes, and taxa with a maximum number of *in-silico* amplicon similarity values ≥97%/species ≥10.

| **ID** | **No. genes**  **(mean)** | **Phylum** | **Class** | **Order** | **Family** | **Genus** | **Species** | **Max. No. ASI≥97/sp. ≥10** |
| --- | --- | --- | --- | --- | --- | --- | --- | --- |
| SP00001 | 1.00 | Crenarchaeota | Thermoprotei | Desulfurococcales | Desulfurococcaceae | Ignisphaera | aggregans | No |
| SP00002 | 3.00 | Euryarchaeota | Methanomicrobia | Methanosarcinales | Methanosarcinaceae | Methanolobus | psychrophilus | No |
| SP00003 | 1.00 | Crenarchaeota | Thermoprotei | Thermoproteales | Thermoproteaceae | Pyrobaculum | oguniense | No |
| SP00004 | 1.00 | Crenarchaeota | Thermoprotei | Desulfurococcales | Desulfurococcaceae | Aeropyrum | pernix | No |
| SP00005 | 2.00 | Euryarchaeota | Methanococci | Methanococcales | Methanocaldococcaceae | Methanocaldococcus | jannaschii | No |
| SP00006 | 1.00 | Euryarchaeota | Thermococci | Thermococcales | Thermococcaceae | Pyrococcus | horikoshii | Yes |
| SP00007 | 3.00 | Euryarchaeota | Methanococci | Methanococcales | Methanococcaceae | Methanococcus | maripaludis | No |
| SP00008 | 1.00 | Euryarchaeota | Halobacteria | Halobacteriales | Halobacteriaceae | Halobacterium | salinarum | No |
| SP00010 | 1.00 | Crenarchaeota | Thermoprotei | Thermoproteales | Thermoproteaceae | Pyrobaculum | aerophilum | No |
| SP00012 | 3.00 | Euryarchaeota | Methanomicrobia | Methanosarcinales | Methanosarcinaceae | Methanosarcina | acetivorans | Yes |
| SP00013 | 3.00 | Euryarchaeota | Methanomicrobia | Methanosarcinales | Methanosarcinaceae | Methanosarcina | mazei | Yes |
| SP00015 | 3.00 | Euryarchaeota | Halobacteria | Halobacteriales | Haloarculaceae | Haloarcula | marismortui | No |
| SP00017 | 3.00 | Euryarchaeota | Methanomicrobia | Methanosarcinales | Methanosarcinaceae | Methanosarcina | barkeri | Yes |
| SP00018 | 1.00 | Euryarchaeota | Halobacteria | Halobacteriales | Haloarculaceae | Natronomonas | pharaonis | No |
| SP00020 | 3.00 | Euryarchaeota | Methanomicrobia | Methanosarcinales | Methanosarcinaceae | Methanococcoides | burtonii | No |
| SP00022 | 3.00 | Euryarchaeota | Methanomicrobia | Methanosarcinales | Methanotrichaceae | Methanothrix | thermoacetophila | No |
| SP00024 | 1.00 | Crenarchaeota | Thermoprotei | Desulfurococcales | Pyrodictiaceae | Hyperthermus | butylicus | No |
| SP00026 | 1.00 | Crenarchaeota | Thermoprotei | Desulfurococcales | Desulfurococcaceae | Staphylothermus | marinus | Yes |
| SP00027 | 1.00 | Euryarchaeota | Methanomicrobia | Methanomicrobiales | Methanomicrobiaceae | Methanoculleus | marisnigri | No |
| SP00028 | 1.00 | Crenarchaeota | Thermoprotei | Thermoproteales | Thermoproteaceae | Pyrobaculum | arsenaticum | No |
| SP00030 | 2.00 | Euryarchaeota | Methanobacteria | Methanobacteriales | Methanobacteriaceae | Methanobrevibacter | smithii | No |
| SP00031 | 4.00 | Euryarchaeota | Methanococci | Methanococcales | Methanococcaceae | Methanococcus | vannielii | No |
| SP00032 | 2.00 | Euryarchaeota | Methanococci | Methanococcales | Methanococcaceae | Methanococcus | aeolicus | No |
| SP00033 | 1.00 | Euryarchaeota | Methanomicrobia | Methanomicrobiales | Methanoregulaceae | Methanoregula | boonei | No |
| SP00034 | 1.00 | Crenarchaeota | Thermoprotei | Desulfurococcales | Desulfurococcaceae | Ignicoccus | hospitalis | No |
| SP00036 | 1.00 | Euryarchaeota | Thermococci | Thermococcales | Thermococcaceae | Thermococcus | onnurineus | Yes |
| SP00037 | 1.00 | Crenarchaeota | Thermoprotei | Desulfurococcales | Desulfurococcaceae | Desulfurococcus | amylolyticus | No |
| SP00038 | 3.00 | Euryarchaeota | Methanomicrobia | Methanomicrobiales | Methanoregulaceae | Methanosphaerula | palustris | No |
| SP00041 | 1.00 | Euryarchaeota | Thermococci | Thermococcales | Thermococcaceae | Thermococcus | gammatolerans | Yes |
| SP00042 | 1.00 | Euryarchaeota | Thermococci | Thermococcales | Thermococcaceae | Thermococcus | sibiricus | Yes |
| SP00043 | 2.00 | Euryarchaeota | Methanococci | Methanococcales | Methanocaldococcaceae | Methanocaldococcus | fervens | No |
| SP00044 | 1.00 | Euryarchaeota | Halobacteria | Halobacteriales | Haloarculaceae | Halorhabdus | utahensis | No |
| SP00045 | 3.00 | Euryarchaeota | Halobacteria | Halobacteriales | Haloarculaceae | Halomicrobium | mukohataei | No |
| SP00046 | 2.00 | Euryarchaeota | Methanococci | Methanococcales | Methanocaldococcaceae | Methanocaldococcus | vulcanius | No |
| SP00048 | 1.00 | Euryarchaeota | Archaeoglobi | Archaeoglobales | Archaeoglobaceae | Archaeoglobus | profundus | No |
| SP00049 | 3.00 | Euryarchaeota | Halobacteria | Natrialbales | Natrialbaceae | Haloterrigena | turkmenica | No |
| SP00050 | 2.00 | Euryarchaeota | Methanobacteria | Methanobacteriales | Methanobacteriaceae | Methanobrevibacter | ruminantium | No |
| SP00051 | 1.00 | Euryarchaeota | Archaeoglobi | Archaeoglobales | Archaeoglobaceae | Ferroglobus | placidus | No |
| SP00052 | 2.00 | Euryarchaeota | Methanococci | Methanococcales | Methanocaldococcaceae | Methanocaldococcus | sp. FS406-22 | No |
| SP00053 | 3.00 | Euryarchaeota | Halobacteria | Natrialbales | Natrialbaceae | Natrialba | magadii | No |
| SP00055 | 3.00 | Euryarchaeota | Methanomicrobia | Methanosarcinales | Methanosarcinaceae | Methanohalophilus | mahii | No |
| SP00056 | 2.00 | Euryarchaeota | Methanococci | Methanococcales | Methanocaldococcaceae | Methanocaldococcus | infernus | No |
| SP00057 | 1.00 | Crenarchaeota | Thermoprotei | Desulfurococcales | Desulfurococcaceae | Staphylothermus | hellenicus | Yes |
| SP00058 | 2.00 | Euryarchaeota | Methanococci | Methanococcales | Methanococcaceae | Methanococcus | voltae | No |
| SP00059 | 2.00 | Euryarchaeota | Methanomicrobia | Methanosarcinales | Methanosarcinaceae | Methanohalobium | evestigatum | No |
| SP00061 | 1.00 | Crenarchaeota | Thermoprotei | Acidilobales | Acidilobaceae | Acidilobus | saccharovorans | No |
| SP00062 | 2.00 | Euryarchaeota | Methanobacteria | Methanobacteriales | Methanobacteriaceae | Methanothermobacter | marburgensis | No |
| SP00064 | 1.00 | Crenarchaeota | Thermoprotei | Thermoproteales | Thermoproteaceae | Vulcanisaeta | distributa | No |
| SP00067 | 1.00 | Euryarchaeota | Thermococci | Thermococcales | Thermococcaceae | Thermococcus | barophilus | Yes |
| SP00068 | 1.00 | Crenarchaeota | Thermoprotei | Desulfurococcales | Desulfurococcaceae | Desulfurococcus | mucosus | No |
| SP00069 | 2.00 | Euryarchaeota | Methanobacteria | Methanobacteriales | Methanobacteriaceae | Methanobacterium | lacus | No |
| SP00070 | 1.00 | Crenarchaeota | Thermoprotei | Thermoproteales | Thermoproteaceae | Thermoproteus | uzoniensis | No |
| SP00071 | 1.00 | Euryarchaeota | Archaeoglobi | Archaeoglobales | Archaeoglobaceae | Archaeoglobus | veneficus | No |
| SP00072 | 3.00 | Euryarchaeota | Methanomicrobia | Methanosarcinales | Methanotrichaceae | Methanothrix | soehngenii | No |
| SP00074 | 1.00 | Euryarchaeota | Thermococci | Thermococcales | Thermococcaceae | Pyrococcus | sp. NA2 | Yes |
| SP00075 | 2.00 | Euryarchaeota | Methanococci | Methanococcales | Methanocaldococcaceae | Methanotorris | igneus | No |
| SP00076 | 3.00 | Euryarchaeota | Methanobacteria | Methanobacteriales | Methanobacteriaceae | Methanobacterium | paludis | No |
| SP00077 | 2.00 | Euryarchaeota | Methanococci | Methanococcales | Methanococcaceae | Methanothermococcus | okinawensis | No |
| SP00078 | 3.00 | Euryarchaeota | Halobacteria | Natrialbales | Natrialbaceae | Halopiger | xanaduensis | No |
| SP00079 | 3.00 | Euryarchaeota | Methanomicrobia | Methanosarcinales | Methanosarcinaceae | Methanosalsum | zhilinae | No |
| SP00080 | 1.00 | Euryarchaeota | Thermococci | Thermococcales | Thermococcaceae | Pyrococcus | yayanosii | Yes |
| SP00081 | 1.00 | Euryarchaeota | Thermococci | Thermococcales | Thermococcaceae | Thermococcus | sp. 4557 | Yes |
| SP00082 | 1.00 | Crenarchaeota | Thermoprotei | Desulfurococcales | Pyrodictiaceae | Pyrolobus | fumarii | No |
| SP00083 | 3.00 | Euryarchaeota | Halobacteria | Halobacteriales | Haloarculaceae | Haloarcula | hispanica | No |
| SP00084 | 1.00 | Crenarchaeota | Thermoprotei | Thermoproteales | Thermoproteaceae | Thermoproteus | tenax | No |
| SP00086 | 1.00 | Crenarchaeota | Thermoprotei | Thermoproteales | Thermoproteaceae | Pyrobaculum | ferrireducens | No |
| SP00089 | 1.00 | Euryarchaeota | Thermococci | Thermococcales | Thermococcaceae | Pyrococcus | sp. ST04 | Yes |
| SP00090 | 1.00 | Crenarchaeota | Thermoprotei | Desulfurococcales | Desulfurococcaceae | Thermogladius | calderae | No |
| SP00091 | 1.00 | Euryarchaeota | Thermococci | Thermococcales | Thermococcaceae | Thermococcus | cleftensis | Yes |
| SP00092 | 3.00 | Euryarchaeota | Halobacteria | Natrialbales | Natrialbaceae | Natrinema | sp. J7-2 | No |
| SP00093 | 1.00 | Euryarchaeota | Methanomicrobia | Methanomicrobiales | Methanomicrobiaceae | Methanoculleus | bourgensis | No |
| SP00094 | 1.00 | Thaumarchaeota | Nitrososphaeria | Nitrososphaerales | Nitrososphaeraceae | Nitrososphaera | gargensis (C.) | No |
| SP00096 | 3.00 | Euryarchaeota | Halobacteria | Natrialbales | Natrialbaceae | Natronobacterium | gregoryi | No |
| SP00097 | 1.00 | Euryarchaeota | Methanomicrobia | Methanomicrobiales | Methanoregulaceae | Methanoregula | formicica | No |
| SP00098 | 3.00 | Euryarchaeota | Halobacteria | Natrialbales | Natrialbaceae | Natrinema | pellirubrum | No |
| SP00099 | 2.00 | Euryarchaeota | Halobacteria | Natrialbales | Natrialbaceae | Halovivax | ruber | No |
| SP00100 | 2.00 | Euryarchaeota | Methanomicrobia | Methanosarcinales | Methanosarcinaceae | Methanomethylovorans | hollandica | No |
| SP00101 | 3.00 | Euryarchaeota | Halobacteria | Natrialbales | Natrialbaceae | Natronococcus | occultus | No |
| SP00102 | 1.00 | Euryarchaeota | Halobacteria | Halobacteriales | Haloarculaceae | Natronomonas | moolapensis | No |
| SP00104 | 1.00 | Euryarchaeota | Archaeoglobi | Archaeoglobales | Archaeoglobaceae | Archaeoglobus | sulfaticallidus | No |
| SP00106 | 3.00 | Euryarchaeota | Methanobacteria | Methanobacteriales | Methanobacteriaceae | Methanobrevibacter | sp. AbM4 | No |
| SP00108 | 1.00 | Euryarchaeota | Halobacteria | Halobacteriales | Haloarculaceae | Halorhabdus | tiamatea | No |
| SP00109 | 1.00 | Euryarchaeota | Thermococci | Thermococcales | Thermococcaceae | Thermococcus | litoralis | Yes |
| SP00110 | 1.00 | Crenarchaeota | Thermoprotei | Desulfurococcales | Desulfurococcaceae | Aeropyrum | camini | No |
| SP00111 | 2.00 | Euryarchaeota | Thermococci | Thermococcales | Thermococcaceae | Palaeococcus | pacificus | Yes |
| SP00112 | 4.00 | Euryarchaeota | Methanobacteria | Methanobacteriales | Methanobacteriaceae | Methanobacterium | formicicum | No |
| SP00113 | 1.00 | Euryarchaeota | Thermococci | Thermococcales | Thermococcaceae | Thermococcus | paralvinellae | Yes |
| SP00114 | 2.00 | Euryarchaeota | Halobacteria | Natrialbales | Natrialbaceae | Halostagnicola | larsenii | No |
| SP00115 | 1.00 | Euryarchaeota | Halobacteria | Halobacteriales | Halobacteriaceae | Halobacterium | sp. DL1 | No |
| SP00116 | 1.00 | Thaumarchaeota | Nitrososphaeria | Nitrososphaerales | Nitrososphaeraceae | Nitrososphaera | evergladensis (C.) | No |
| SP00117 | 1.00 | Thaumarchaeota | Nitrososphaeria | Nitrososphaerales | Nitrososphaeraceae | Nitrososphaera | viennensis | No |
| SP00118 | 1.00 | Euryarchaeota | Thermococci | Thermococcales | Thermococcaceae | Thermococcus | eurythermalis | Yes |
| SP00119 | 2.00 | Euryarchaeota | Methanococci | Methanococcales | Methanocaldococcaceae | Methanocaldococcus | bathoardescens | No |
| SP00120 | 3.00 | Euryarchaeota | Methanomicrobia | Methanosarcinales | Methanosarcinaceae | Methanosarcina | thermophila | Yes |
| SP00121 | 3.00 | Euryarchaeota | Methanomicrobia | Methanosarcinales | Methanosarcinaceae | Methanosarcina | sp. WWM596 | Yes |
| SP00122 | 3.00 | Euryarchaeota | Methanomicrobia | Methanosarcinales | Methanosarcinaceae | Methanosarcina | sp. WH1 | Yes |
| SP00123 | 3.00 | Euryarchaeota | Methanomicrobia | Methanosarcinales | Methanosarcinaceae | Methanosarcina | sp. MTP4 | No |
| SP00124 | 3.00 | Euryarchaeota | Methanomicrobia | Methanosarcinales | Methanosarcinaceae | Methanosarcina | siciliae | Yes |
| SP00125 | 3.00 | Euryarchaeota | Methanomicrobia | Methanosarcinales | Methanosarcinaceae | Methanosarcina | lacustris | Yes |
| SP00126 | 3.00 | Euryarchaeota | Methanomicrobia | Methanosarcinales | Methanosarcinaceae | Methanosarcina | horonobensis | Yes |
| SP00127 | 3.00 | Euryarchaeota | Methanomicrobia | Methanosarcinales | Methanosarcinaceae | Methanococcoides | methylutens | No |
| SP00128 | 3.00 | Euryarchaeota | Methanomicrobia | Methanosarcinales | Methanosarcinaceae | Methanosarcina | vacuolata | Yes |
| SP00129 | 3.00 | Euryarchaeota | Methanomicrobia | Methanosarcinales | Methanosarcinaceae | Methanosarcina | sp. Kolksee | Yes |
| SP00131 | 3.00 | Euryarchaeota | Halobacteria | Halobacteriales | Haloarculaceae | Haloarcula | sp. CBA1115 | No |
| SP00132 | 2.00 | Euryarchaeota | Methanobacteria | Methanobacteriales | Methanobacteriaceae | Methanobrevibacter | millerae | No |
| SP00133 | 2.00 | Euryarchaeota | Halobacteria | Halobacteriales | Haloarculaceae | Halomicrobium | sp. ZPS1 | No |
| SP00134 | 4.00 | Euryarchaeota | Methanobacteria | Methanobacteriales | Methanobacteriaceae | Methanosphaera | stadtmanae | No |
| SP00135 | 3.00 | Euryarchaeota | Methanobacteria | Methanobacteriales | Methanobacteriaceae | Methanobacterium | congolense | No |

The Table details the archaeal species which had *in-silico* amplicon similarity values ≥97% with at least one different taxon using the archaeal-specific and the bacterial and archaeal primer pairs analyzed in the present study, signalling those with a maximum number of in-silico amplicon similarity values ≥97%/species ≥10. The mean number of intragenomic 16S rRNA genes of these species was calculated in a previous investigation of our group (Regueira-Iglesias et al. 2021.b). ASI97= *in silico* amplicon similarity values ≥97%; C.= Candidatus; ID= species identifier; Max.= maximum; No.= number; sp.= species.

Supplementary Table 3. Pairs of bacterial species with *in-silico* amplicon similarity values ≥97% using the analyzed primer pairs.

| **ID** | **Genus** | **Species** | **ID** | **Genus** | **Species** | **Frequency** |
| --- | --- | --- | --- | --- | --- | --- |
| SP00141 | Neisseria | meningitidis | SP00144 | Neisseria | Gonorrhoeae | 29 |
| SP00146 | Streptococcus | pneumoniae | SP00288 | Streptococcus | sp. oral taxon 431 | 29 |
| SP00146 | Streptococcus | pneumoniae | SP00315 | Streptococcus | Mitis | 29 |
| SP00146 | Streptococcus | pneumoniae | SP00319 | Streptococcus | Oralis | 29 |
| SP00154 | Staphylococcus | epidermidis | SP00248 | Staphylococcus | Warneri | 29 |
| SP00154 | Staphylococcus | epidermidis | SP00252 | Staphylococcus | Pasteuri | 29 |
| SP00154 | Staphylococcus | epidermidis | SP00258 | Staphylococcus | Capitis | 29 |
| SP00158 | Lactobacillus | johnsonii | SP00195 | Lactobacillus | Gasseri | 29 |
| SP00242 | Streptococcus | salivarius | SP00320 | Streptococcus | Thermophilus | 29 |
| SP00248 | Staphylococcus | warneri | SP00252 | Staphylococcus | Pasteuri | 29 |
| SP00248 | Staphylococcus | warneri | SP00258 | Staphylococcus | Capitis | 29 |
| SP00252 | Staphylococcus | pasteuri | SP00258 | Staphylococcus | Capitis | 29 |
| SP00279 | Staphylococcus | haemolyticus | SP00280 | Staphylococcus | Lugdunensis | 29 |
| SP00279 | Staphylococcus | haemolyticus | SP00297 | Staphylococcus | Cohnii | 29 |
| SP00286 | Actinomyces | oris | SP00314 | Actinomyces | sp. oral taxon 169 | 29 |
| SP00288 | Streptococcus | sp. oral taxon 431 | SP00315 | Streptococcus | Mitis | 29 |
| SP00288 | Streptococcus | sp. oral taxon 431 | SP00319 | Streptococcus | Oralis | 29 |
| SP00315 | Streptococcus | mitis | SP00319 | Streptococcus | Oralis | 29 |
| SP00149 | Fusobacterium | nucleatum | SP00278 | Fusobacterium | Hwasookii | 28 |
| SP00154 | Staphylococcus | epidermidis | SP00163 | Staphylococcus | Aureus | 28 |
| SP00154 | Staphylococcus | epidermidis | SP00280 | Staphylococcus | Lugdunensis | 28 |
| SP00163 | Staphylococcus | aureus | SP00258 | Staphylococcus | Capitis | 28 |
| SP00205 | Klebsiella | variicola | SP00172 | Klebsiella | Pneumoniae | 28 |
| SP00240 | Klebsiella | aerogenes | SP00172 | Klebsiella | Pneumoniae | 28 |
| SP00189 | Lactobacillus | acidophilus | SP00235 | Lactobacillus | Amylovorus | 28 |
| SP00196 | Lacticaseibacillus | paracasei | SP00243 | Lacticaseibacillus | Rhamnosus | 28 |
| SP00202 | Cronobacter | sakazakii | SP00255 | Cronobacter | Malonaticus | 28 |
| SP00215 | Capnocytophaga | ochracea | SP00273 | Capnocytophaga | sp. oral taxon 323 | 28 |
| SP00280 | Staphylococcus | lugdunensis | SP00297 | Staphylococcus | Cohnii | 28 |
| SP00138 | Mycobacterium | tuberculosis | SP00164 | Mycobacterium | Leprae | 27 |
| SP00154 | Staphylococcus | epidermidis | SP00279 | Staphylococcus | Haemolyticus | 27 |
| SP00154 | Staphylococcus | epidermidis | SP00302 | Staphylococcus | Pettenkoferi | 27 |
| SP00159 | Treponema | denticola | SP00321 | Treponema | Putidum | 27 |
| SP00163 | Staphylococcus | aureus | SP00248 | Staphylococcus | Warneri | 27 |
| SP00163 | Staphylococcus | aureus | SP00252 | Staphylococcus | Pasteuri | 27 |
| SP00163 | Staphylococcus | aureus | SP00279 | Staphylococcus | Haemolyticus | 27 |
| SP00163 | Staphylococcus | aureus | SP00280 | Staphylococcus | Lugdunensis | 27 |
| SP00163 | Staphylococcus | aureus | SP00297 | Staphylococcus | Cohnii | 27 |
| SP00163 | Staphylococcus | aureus | SP00302 | Staphylococcus | Pettenkoferi | 27 |
| SP00166 | Pseudomonas | fluorescens | SP00191 | Pseudomonas | Protegens | 27 |
| SP00186 | Staphylococcus | schleiferi | SP00280 | Staphylococcus | lugdunensis | 27 |
| SP00194 | Streptococcus | sanguinis | SP00288 | Streptococcus | sp. oral taxon 431 | 27 |
| SP00194 | Streptococcus | sanguinis | SP00315 | Streptococcus | mitis | 27 |
| SP00194 | Streptococcus | sanguinis | SP00319 | Streptococcus | oralis | 27 |
| SP00248 | Staphylococcus | warneri | SP00280 | Staphylococcus | lugdunensis | 27 |
| SP00248 | Staphylococcus | warneri | SP00297 | Staphylococcus | cohnii | 27 |
| SP00252 | Staphylococcus | pasteuri | SP00280 | Staphylococcus | lugdunensis | 27 |
| SP00252 | Staphylococcus | pasteuri | SP00297 | Staphylococcus | cohnii | 27 |
| SP00258 | Staphylococcus | capitis | SP00280 | Staphylococcus | lugdunensis | 27 |
| SP00258 | Staphylococcus | capitis | SP00302 | Staphylococcus | pettenkoferi | 27 |
| SP00266 | Selenomonas | sp. oral taxon 478 | SP00295 | Selenomonas | sp. oral taxon 920 | 27 |
| SP00280 | Staphylococcus | lugdunensis | SP00302 | Staphylococcus | pettenkoferi | 27 |
| SP00286 | Actinomyces | oris | SP00312 | Actinomyces | sp. oral taxon 171 | 27 |
| SP00312 | Actinomyces | sp. oral taxon 171 | SP00314 | Actinomyces | sp. oral taxon 169 | 27 |
| SP00194 | Streptococcus | sanguinis | SP00146 | Streptococcus | pneumoniae | 26 |
| SP00251 | Streptococcus | constellatus | SP00177 | Streptococcus | intermedius | 26 |
| SP00205 | Klebsiella | variicola | SP00240 | Klebsiella | aerogenes | 26 |
| SP00258 | Staphylococcus | capitis | SP00279 | Staphylococcus | haemolyticus | 26 |
| SP00279 | Staphylococcus | haemolyticus | SP00302 | Staphylococcus | pettenkoferi | 26 |
| SP00297 | Staphylococcus | cohnii | SP00302 | Staphylococcus | pettenkoferi | 26 |
| SP00154 | Staphylococcus | epidermidis | SP00186 | Staphylococcus | schleiferi | 25 |
| SP00154 | Staphylococcus | epidermidis | SP00297 | Staphylococcus | cohnii | 25 |
| SP00186 | Staphylococcus | schleiferi | SP00163 | Staphylococcus | aureus | 25 |
| SP00166 | Pseudomonas | fluorescens | SP00193 | Pseudomonas | stutzeri | 25 |
| SP00186 | Staphylococcus | schleiferi | SP00248 | Staphylococcus | warneri | 25 |
| SP00186 | Staphylococcus | schleiferi | SP00252 | Staphylococcus | pasteuri | 25 |
| SP00186 | Staphylococcus | schleiferi | SP00258 | Staphylococcus | capitis | 25 |
| SP00188 | Bordetella | pertussis | SP00233 | Achromobacter | xylosoxidans | 25 |
| SP00194 | Streptococcus | sanguinis | SP00199 | Streptococcus | gordonii | 25 |
| SP00197 | Acinetobacter | baumannii | SP00296 | Acinetobacter | junii | 25 |
| SP00229 | Olsenella | uli | SP00264 | Olsenella | sp. oral taxon 807 | 25 |
| SP00241 | Streptococcus | parasanguinis | SP00315 | Streptococcus | mitis | 25 |
| SP00241 | Streptococcus | parasanguinis | SP00319 | Streptococcus | oralis | 25 |
| SP00248 | Staphylococcus | warneri | SP00279 | Staphylococcus | haemolyticus | 25 |
| SP00248 | Staphylococcus | warneri | SP00302 | Staphylococcus | pettenkoferi | 25 |
| SP00252 | Staphylococcus | pasteuri | SP00279 | Staphylococcus | haemolyticus | 25 |
| SP00252 | Staphylococcus | pasteuri | SP00302 | Staphylococcus | pettenkoferi | 25 |
| SP00258 | Staphylococcus | capitis | SP00297 | Staphylococcus | cohnii | 25 |
| SP00194 | Streptococcus | sanguinis | SP00241 | Streptococcus | parasanguinis | 24 |
| SP00194 | Streptococcus | sanguinis | SP00251 | Streptococcus | constellatus | 24 |
| SP00241 | Streptococcus | parasanguinis | SP00288 | Streptococcus | sp. oral taxon 431 | 24 |
| SP00262 | Corynebacterium | singulare | SP00289 | Corynebacterium | simulans | 24 |
| SP00273 | Capnocytophaga | sp. oral taxon 323 | SP00305 | Capnocytophaga | sp. oral taxon 878 | 24 |
| SP00289 | Corynebacterium | simulans | SP00300 | Corynebacterium | striatum | 24 |
| SP00316 | Neisseria | lactamica | SP00141 | Neisseria | meningitidis | 23 |
| SP00172 | Klebsiella | pneumoniae | SP00275 | Serratia | marcescens | 23 |
| SP00184 | Streptococcus | anginosus | SP00177 | Streptococcus | intermedius | 23 |
| SP00186 | Staphylococcus | schleiferi | SP00279 | Staphylococcus | haemolyticus | 23 |
| SP00186 | Staphylococcus | schleiferi | SP00297 | Staphylococcus | cohnii | 23 |
| SP00186 | Staphylococcus | schleiferi | SP00302 | Staphylococcus | pettenkoferi | 23 |
| SP00199 | Streptococcus | gordonii | SP00288 | Streptococcus | sp. oral taxon 431 | 23 |
| SP00199 | Streptococcus | gordonii | SP00315 | Streptococcus | mitis | 23 |
| SP00199 | Streptococcus | gordonii | SP00319 | Streptococcus | oralis | 23 |
| SP00205 | Klebsiella | variicola | SP00275 | Serratia | marcescens | 23 |
| SP00206 | Ralstonia | pickettii | SP00261 | Cupriavidus | gilardii | 23 |
| SP00215 | Capnocytophaga | ochracea | SP00305 | Capnocytophaga | sp. oral taxon 878 | 23 |
| SP00142 | Pseudomonas | aeruginosa | SP00193 | Pseudomonas | stutzeri | 22 |
| SP00146 | Streptococcus | pneumoniae | SP00241 | Streptococcus | parasanguinis | 22 |
| SP00184 | Streptococcus | anginosus | SP00251 | Streptococcus | constellatus | 22 |
| SP00241 | Streptococcus | parasanguinis | SP00253 | Streptococcus | cristatus | 22 |
| SP00260 | Acinetobacter | johnsonii | SP00296 | Acinetobacter | junii | 22 |
| SP00267 | Schaalia | meyeri | SP00313 | Schaalia | odontolytica | 22 |
| SP00199 | Streptococcus | gordonii | SP00146 | Streptococcus | pneumoniae | 21 |
| SP00194 | Streptococcus | sanguinis | SP00177 | Streptococcus | intermedius | 21 |
| SP00199 | Streptococcus | gordonii | SP00241 | Streptococcus | parasanguinis | 21 |
| SP00240 | Klebsiella | aerogenes | SP00275 | Serratia | marcescens | 21 |
| SP00136 | Escherichia | coli | SP00275 | Serratia | marcescens | 20 |
| SP00199 | Streptococcus | gordonii | SP00177 | Streptococcus | intermedius | 20 |
| SP00199 | Streptococcus | gordonii | SP00253 | Streptococcus | cristatus | 20 |
| SP00202 | Cronobacter | sakazakii | SP00240 | Klebsiella | aerogenes | 20 |
| SP00262 | Corynebacterium | singulare | SP00300 | Corynebacterium | striatum | 20 |
| SP00308 | Haemophilus | sp. oral taxon 036 | SP00190 | Haemophilus | influenzae | 19 |
| SP00201 | Campylobacter | curvus | SP00203 | Campylobacter | concisus | 19 |
| SP00202 | Cronobacter | sakazakii | SP00136 | Escherichia | coli | 18 |
| SP00136 | Escherichia | coli | SP00255 | Cronobacter | malonaticus | 18 |
| SP00144 | Neisseria | gonorrhoeae | SP00316 | Neisseria | lactamica | 18 |
| SP00202 | Cronobacter | sakazakii | SP00172 | Klebsiella | pneumoniae | 18 |
| SP00172 | Klebsiella | pneumoniae | SP00255 | Cronobacter | malonaticus | 18 |
| SP00178 | Rothia | mucilaginosa | SP00232 | Rothia | dentocariosa | 18 |
| SP00197 | Acinetobacter | baumannii | SP00260 | Acinetobacter | johnsonii | 18 |
| SP00209 | Comamonas | thiooxydans | SP00211 | Acidovorax | ebreus | 18 |
| SP00212 | Aggregatibacter | aphrophilus | SP00308 | Haemophilus | sp. oral taxon 036 | 18 |
| SP00241 | Streptococcus | parasanguinis | SP00251 | Streptococcus | constellatus | 18 |
| SP00253 | Streptococcus | cristatus | SP00288 | Streptococcus | sp. oral taxon 431 | 18 |
| SP00272 | Kocuria | palustris | SP00301 | Kocuria | rhizophila | 18 |
| SP00152 | Bifidobacterium | longum | SP00192 | Bifidobacterium | breve | 17 |
| SP00161 | Cutibacterium | acnes | SP00294 | Propionibacterium | sp. oral taxon 193 | 17 |
| SP00191 | Pseudomonas | protegens | SP00193 | Pseudomonas | stutzeri | 17 |
| SP00253 | Streptococcus | cristatus | SP00315 | Streptococcus | mitis | 17 |
| SP00253 | Streptococcus | cristatus | SP00319 | Streptococcus | oralis | 17 |
| SP00172 | Klebsiella | pneumoniae | SP00136 | Escherichia | coli | 16 |
| SP00146 | Streptococcus | pneumoniae | SP00253 | Streptococcus | cristatus | 16 |
| SP00150 | Yersinia | pestis | SP00275 | Serratia | marcescens | 16 |
| SP00240 | Klebsiella | aerogenes | SP00255 | Cronobacter | malonaticus | 16 |
| SP00148 | Streptococcus | agalactiae | SP00143 | Streptococcus | pyogenes | 15 |
| SP00241 | Streptococcus | parasanguinis | SP00177 | Streptococcus | intermedius | 15 |
| SP00194 | Streptococcus | sanguinis | SP00253 | Streptococcus | cristatus | 15 |
| SP00205 | Klebsiella | variicola | SP00136 | Escherichia | coli | 14 |
| SP00177 | Streptococcus | intermedius | SP00253 | Streptococcus | cristatus | 14 |
| SP00181 | Levilactobacillus | brevis | SP00237 | Lentilactobacillus | buchneri | 14 |
| SP00184 | Streptococcus | anginosus | SP00194 | Streptococcus | sanguinis | 14 |
| SP00199 | Streptococcus | gordonii | SP00251 | Streptococcus | constellatus | 14 |
| SP00202 | Cronobacter | sakazakii | SP00205 | Klebsiella | variicola | 14 |
| SP00184 | Streptococcus | anginosus | SP00199 | Streptococcus | gordonii | 13 |
| SP00184 | Streptococcus | anginosus | SP00253 | Streptococcus | cristatus | 13 |
| SP00205 | Klebsiella | variicola | SP00255 | Cronobacter | malonaticus | 13 |
| SP00210 | Rhodobacter | capsulatus | SP00298 | Paracoccus | yeei | 13 |
| SP00219 | Kytococcus | sedentarius | SP00277 | Janibacter | indicus | 13 |
| SP00138 | Mycobacterium | tuberculosis | SP00256 | Mycolicibacterium | neoaurum | 12 |
| SP00177 | Streptococcus | intermedius | SP00143 | Streptococcus | pyogenes | 11 |
| SP00199 | Streptococcus | gordonii | SP00143 | Streptococcus | pyogenes | 11 |
| SP00242 | Streptococcus | salivarius | SP00177 | Streptococcus | intermedius | 11 |
| SP00182 | Mycoplasma | pneumoniae | SP00249 | Mycoplasma | genitalium | 11 |
| SP00199 | Streptococcus | gordonii | SP00320 | Streptococcus | thermophilus | 11 |
| SP00202 | Cronobacter | sakazakii | SP00275 | Serratia | marcescens | 11 |
| SP00251 | Streptococcus | constellatus | SP00315 | Streptococcus | mitis | 11 |
| SP00251 | Streptococcus | constellatus | SP00319 | Streptococcus | oralis | 11 |
| SP00255 | Cronobacter | malonaticus | SP00275 | Serratia | marcescens | 11 |
| SP00282 | Actinomyces | radicidentis | SP00312 | Actinomyces | sp. oral taxon 171 | 11 |
| SP00282 | Actinomyces | radicidentis | SP00314 | Actinomyces | sp. oral taxon 169 | 11 |
| SP00152 | Bifidobacterium | longum | SP00223 | Bifidobacterium | dentium | 10 |
| SP00242 | Streptococcus | salivarius | SP00184 | Streptococcus | anginosus | 10 |
| SP00184 | Streptococcus | anginosus | SP00320 | Streptococcus | thermophilus | 10 |
| SP00230 | Prevotella | melaninogenica | SP00269 | Prevotella | fusca | 10 |
| SP00242 | Streptococcus | salivarius | SP00253 | Streptococcus | cristatus | 10 |
| SP00251 | Streptococcus | constellatus | SP00253 | Streptococcus | cristatus | 10 |
| SP00251 | Streptococcus | constellatus | SP00288 | Streptococcus | sp. oral taxon 431 | 10 |
| SP00136 | Escherichia | coli | SP00240 | Klebsiella | aerogenes | 9 |
| SP00251 | Streptococcus | constellatus | SP00146 | Streptococcus | pneumoniae | 9 |
| SP00148 | Streptococcus | agalactiae | SP00194 | Streptococcus | sanguinis | 9 |
| SP00150 | Yersinia | pestis | SP00240 | Klebsiella | aerogenes | 9 |
| SP00164 | Mycobacterium | leprae | SP00256 | Mycolicibacterium | neoaurum | 9 |
| SP00177 | Streptococcus | intermedius | SP00320 | Streptococcus | thermophilus | 9 |
| SP00192 | Bifidobacterium | breve | SP00223 | Bifidobacterium | dentium | 9 |
| SP00194 | Streptococcus | sanguinis | SP00242 | Streptococcus | salivarius | 9 |
| SP00194 | Streptococcus | sanguinis | SP00320 | Streptococcus | thermophilus | 9 |
| SP00199 | Streptococcus | gordonii | SP00242 | Streptococcus | salivarius | 9 |
| SP00253 | Streptococcus | cristatus | SP00320 | Streptococcus | thermophilus | 9 |
| SP00282 | Actinomyces | radicidentis | SP00286 | Actinomyces | oris | 9 |
| SP00194 | Streptococcus | sanguinis | SP00143 | Streptococcus | pyogenes | 8 |
| SP00184 | Streptococcus | anginosus | SP00241 | Streptococcus | parasanguinis | 8 |
| SP00187 | Corynebacterium | diphtheriae | SP00262 | Corynebacterium | singulare | 8 |
| SP00187 | Corynebacterium | diphtheriae | SP00289 | Corynebacterium | simulans | 8 |
| SP00212 | Aggregatibacter | aphrophilus | SP00190 | Haemophilus | influenzae | 8 |
| SP00211 | Acidovorax | ebreus | SP00216 | Variovorax | paradoxus | 8 |
| SP00241 | Streptococcus | parasanguinis | SP00242 | Streptococcus | salivarius | 8 |
| SP00241 | Streptococcus | parasanguinis | SP00320 | Streptococcus | thermophilus | 8 |
| SP00244 | Tannerella | forsythia | SP00293 | Tannerella | sp. oral taxon HOT-286 | 8 |
| SP00136 | Escherichia | coli | SP00150 | Yersinia | pestis | 7 |
| SP00146 | Streptococcus | pneumoniae | SP00177 | Streptococcus | intermedius | 7 |
| SP00150 | Yersinia | pestis | SP00172 | Klebsiella | pneumoniae | 7 |
| SP00150 | Yersinia | pestis | SP00202 | Cronobacter | sakazakii | 7 |
| SP00150 | Yersinia | pestis | SP00205 | Klebsiella | variicola | 7 |
| SP00150 | Yersinia | pestis | SP00255 | Cronobacter | malonaticus | 7 |
| SP00161 | Cutibacterium | acnes | SP00254 | Cutibacterium | avidum | 7 |
| SP00165 | Lactiplantibacillus | plantarum | SP00181 | Levilactobacillus | brevis | 7 |
| SP00168 | Corynebacterium | urealyticum | SP00262 | Corynebacterium | singulare | 7 |
| SP00173 | Limosilactobacillus | reuteri | SP00174 | Limosilactobacillus | fermentum | 7 |
| SP00173 | Limosilactobacillus | reuteri | SP00237 | Lentilactobacillus | buchneri | 7 |
| SP00209 | Comamonas | thiooxydans | SP00216 | Variovorax | paradoxus | 7 |
| SP00254 | Cutibacterium | avidum | SP00294 | Propionibacterium | sp. oral taxon 193 | 7 |
| SP00152 | Bifidobacterium | longum | SP00208 | Bifidobacterium | animalis | 6 |
| SP00165 | Lactiplantibacillus | plantarum | SP00196 | Lacticaseibacillus | paracasei | 6 |
| SP00165 | Lactiplantibacillus | plantarum | SP00237 | Lentilactobacillus | buchneri | 6 |
| SP00165 | Lactiplantibacillus | plantarum | SP00243 | Lacticaseibacillus | rhamnosus | 6 |
| SP00181 | Levilactobacillus | brevis | SP00243 | Lacticaseibacillus | rhamnosus | 6 |
| SP00318 | Haemophilus | parainfluenzae | SP00190 | Haemophilus | influenzae | 6 |
| SP00204 | Delftia | acidovorans | SP00216 | Variovorax | paradoxus | 6 |
| SP00223 | Bifidobacterium | dentium | SP00208 | Bifidobacterium | animalis | 6 |
| SP00237 | Lentilactobacillus | buchneri | SP00243 | Lacticaseibacillus | rhamnosus | 6 |
| SP00242 | Streptococcus | salivarius | SP00251 | Streptococcus | constellatus | 6 |
| SP00251 | Streptococcus | constellatus | SP00320 | Streptococcus | thermophilus | 6 |
| SP00306 | Bacteroides | zoogleoformans | SP00307 | Bacteroides | heparinolyticus | 6 |
| SP00143 | Streptococcus | pyogenes | SP00288 | Streptococcus | sp. oral taxon 431 | 5 |
| SP00143 | Streptococcus | pyogenes | SP00315 | Streptococcus | mitis | 5 |
| SP00143 | Streptococcus | pyogenes | SP00319 | Streptococcus | oralis | 5 |
| SP00148 | Streptococcus | agalactiae | SP00199 | Streptococcus | gordonii | 5 |
| SP00148 | Streptococcus | agalactiae | SP00315 | Streptococcus | mitis | 5 |
| SP00148 | Streptococcus | agalactiae | SP00319 | Streptococcus | oralis | 5 |
| SP00151 | Streptococcus | mutans | SP00184 | Streptococcus | anginosus | 5 |
| SP00177 | Streptococcus | intermedius | SP00315 | Streptococcus | mitis | 5 |
| SP00177 | Streptococcus | intermedius | SP00319 | Streptococcus | oralis | 5 |
| SP00181 | Levilactobacillus | brevis | SP00196 | Lacticaseibacillus | paracasei | 5 |
| SP00185 | Aggregatibacter | actinomycetemcomitans | SP00212 | Aggregatibacter | aphrophilus | 5 |
| SP00187 | Corynebacterium | diphtheriae | SP00300 | Corynebacterium | striatum | 5 |
| SP00192 | Bifidobacterium | breve | SP00208 | Bifidobacterium | animalis | 5 |
| SP00196 | Lacticaseibacillus | paracasei | SP00237 | Lentilactobacillus | buchneri | 5 |
| SP00308 | Haemophilus | sp. oral taxon 036 | SP00318 | Haemophilus | parainfluenzae | 5 |
| SP00143 | Streptococcus | pyogenes | SP00146 | Streptococcus | pneumoniae | 4 |
| SP00143 | Streptococcus | pyogenes | SP00151 | Streptococcus | mutans | 4 |
| SP00143 | Streptococcus | pyogenes | SP00184 | Streptococcus | anginosus | 4 |
| SP00143 | Streptococcus | pyogenes | SP00251 | Streptococcus | constellatus | 4 |
| SP00147 | Agrobacterium | fabrum | SP00198 | Agrobacterium | radiobacter | 4 |
| SP00147 | Agrobacterium | fabrum | SP00200 | Brucella | anthropi | 4 |
| SP00148 | Streptococcus | agalactiae | SP00177 | Streptococcus | intermedius | 4 |
| SP00148 | Streptococcus | agalactiae | SP00251 | Streptococcus | constellatus | 4 |
| SP00148 | Streptococcus | agalactiae | SP00288 | Streptococcus | sp. oral taxon 431 | 4 |
| SP00151 | Streptococcus | mutans | SP00199 | Streptococcus | gordonii | 4 |
| SP00155 | Enterococcus | faecalis | SP00160 | Listeria | monocytogenes | 4 |
| SP00157 | Haemophilus | ducreyi | SP00318 | Haemophilus | parainfluenzae | 4 |
| SP00187 | Corynebacterium | diphtheriae | SP00168 | Corynebacterium | urealyticum | 4 |
| SP00198 | Agrobacterium | radiobacter | SP00200 | Brucella | anthropi | 4 |
| SP00214 | Micrococcus | luteus | SP00272 | Kocuria | palustris | 4 |
| SP00218 | Leptotrichia | buccalis | SP00291 | Leptotrichia | sp. oral taxon 498 | 4 |
| SP00224 | Sanguibacter | keddieii | SP00277 | Janibacter | indicus | 4 |
| SP00146 | Streptococcus | pneumoniae | SP00148 | Streptococcus | agalactiae | 3 |
| SP00151 | Streptococcus | mutans | SP00148 | Streptococcus | agalactiae | 3 |
| SP00148 | Streptococcus | agalactiae | SP00184 | Streptococcus | anginosus | 3 |
| SP00148 | Streptococcus | agalactiae | SP00241 | Streptococcus | parasanguinis | 3 |
| SP00151 | Streptococcus | mutans | SP00242 | Streptococcus | salivarius | 3 |
| SP00151 | Streptococcus | mutans | SP00320 | Streptococcus | thermophilus | 3 |
| SP00156 | Bacillus | anthracis | SP00179 | Bacillus | subtilis | 3 |
| SP00156 | Bacillus | anthracis | SP00297 | Staphylococcus | cohnii | 3 |
| SP00157 | Haemophilus | ducreyi | SP00190 | Haemophilus | influenzae | 3 |
| SP00157 | Haemophilus | ducreyi | SP00308 | Haemophilus | sp. oral taxon 036 | 3 |
| SP00158 | Lactobacillus | johnsonii | SP00189 | Lactobacillus | acidophilus | 3 |
| SP00158 | Lactobacillus | johnsonii | SP00235 | Lactobacillus | amylovorus | 3 |
| SP00160 | Listeria | monocytogenes | SP00163 | Staphylococcus | aureus | 3 |
| SP00160 | Listeria | monocytogenes | SP00280 | Staphylococcus | lugdunensis | 3 |
| SP00160 | Listeria | monocytogenes | SP00297 | Staphylococcus | cohnii | 3 |
| SP00168 | Corynebacterium | urealyticum | SP00289 | Corynebacterium | simulans | 3 |
| SP00168 | Corynebacterium | urealyticum | SP00300 | Corynebacterium | striatum | 3 |
| SP00170 | Mesorhizobium | japonicum | SP00198 | Agrobacterium | radiobacter | 3 |
| SP00170 | Mesorhizobium | japonicum | SP00200 | Brucella | anthropi | 3 |
| SP00181 | Levilactobacillus | brevis | SP00173 | Limosilactobacillus | reuteri | 3 |
| SP00173 | Limosilactobacillus | reuteri | SP00243 | Lacticaseibacillus | rhamnosus | 3 |
| SP00177 | Streptococcus | intermedius | SP00288 | Streptococcus | sp. oral taxon 431 | 3 |
| SP00178 | Rothia | mucilaginosa | SP00272 | Kocuria | palustris | 3 |
| SP00187 | Corynebacterium | diphtheriae | SP00259 | Corynebacterium | sp. ATCC 6931 | 3 |
| SP00189 | Lactobacillus | acidophilus | SP00195 | Lactobacillus | gasseri | 3 |
| SP00195 | Lactobacillus | gasseri | SP00235 | Lactobacillus | amylovorus | 3 |
| SP00204 | Delftia | acidovorans | SP00209 | Comamonas | thiooxydans | 3 |
| SP00213 | Corynebacterium | kroppenstedtii | SP00259 | Corynebacterium | sp. ATCC 6931 | 3 |
| SP00214 | Micrococcus | luteus | SP00224 | Sanguibacter | keddieii | 3 |
| SP00224 | Sanguibacter | keddieii | SP00265 | Arsenicicoccus | sp. oral taxon 190 | 3 |
| SP00259 | Corynebacterium | sp. ATCC 6931 | SP00262 | Corynebacterium | singulare | 3 |
| SP00259 | Corynebacterium | sp. ATCC 6931 | SP00289 | Corynebacterium | simulans | 3 |
| SP00259 | Corynebacterium | sp. ATCC 6931 | SP00300 | Corynebacterium | striatum | 3 |
| SP00265 | Arsenicicoccus | sp. oral taxon 190 | SP00277 | Janibacter | indicus | 3 |
| SP00267 | Schaalia | meyeri | SP00282 | Actinomyces | radicidentis | 3 |
| SP00267 | Schaalia | meyeri | SP00286 | Actinomyces | oris | 3 |
| SP00267 | Schaalia | meyeri | SP00312 | Actinomyces | sp. oral taxon 171 | 3 |
| SP00267 | Schaalia | meyeri | SP00314 | Actinomyces | sp. oral taxon 169 | 3 |
| SP00286 | Actinomyces | oris | SP00313 | Schaalia | odontolytica | 3 |
| SP00312 | Actinomyces | sp. oral taxon 171 | SP00313 | Schaalia | odontolytica | 3 |
| SP00313 | Schaalia | odontolytica | SP00314 | Actinomyces | sp. oral taxon 169 | 3 |
| SP00136 | Escherichia | coli | SP00318 | Haemophilus | parainfluenzae | 2 |
| SP00143 | Streptococcus | pyogenes | SP00241 | Streptococcus | parasanguinis | 2 |
| SP00143 | Streptococcus | pyogenes | SP00242 | Streptococcus | salivarius | 2 |
| SP00143 | Streptococcus | pyogenes | SP00253 | Streptococcus | cristatus | 2 |
| SP00143 | Streptococcus | pyogenes | SP00320 | Streptococcus | thermophilus | 2 |
| SP00151 | Streptococcus | mutans | SP00177 | Streptococcus | intermedius | 2 |
| SP00151 | Streptococcus | mutans | SP00194 | Streptococcus | sanguinis | 2 |
| SP00151 | Streptococcus | mutans | SP00251 | Streptococcus | constellatus | 2 |
| SP00154 | Staphylococcus | epidermidis | SP00160 | Listeria | monocytogenes | 2 |
| SP00154 | Staphylococcus | epidermidis | SP00179 | Bacillus | subtilis | 2 |
| SP00181 | Levilactobacillus | brevis | SP00155 | Enterococcus | faecalis | 2 |
| SP00155 | Enterococcus | faecalis | SP00237 | Lentilactobacillus | buchneri | 2 |
| SP00155 | Enterococcus | faecalis | SP00297 | Staphylococcus | cohnii | 2 |
| SP00160 | Listeria | monocytogenes | SP00179 | Bacillus | subtilis | 2 |
| SP00160 | Listeria | monocytogenes | SP00186 | Staphylococcus | schleiferi | 2 |
| SP00160 | Listeria | monocytogenes | SP00248 | Staphylococcus | warneri | 2 |
| SP00160 | Listeria | monocytogenes | SP00252 | Staphylococcus | pasteuri | 2 |
| SP00160 | Listeria | monocytogenes | SP00258 | Staphylococcus | capitis | 2 |
| SP00160 | Listeria | monocytogenes | SP00279 | Staphylococcus | haemolyticus | 2 |
| SP00160 | Listeria | monocytogenes | SP00302 | Staphylococcus | pettenkoferi | 2 |
| SP00196 | Lacticaseibacillus | paracasei | SP00162 | Ligilactobacillus | salivarius | 2 |
| SP00162 | Ligilactobacillus | salivarius | SP00243 | Lacticaseibacillus | rhamnosus | 2 |
| SP00163 | Staphylococcus | aureus | SP00179 | Bacillus | subtilis | 2 |
| SP00172 | Klebsiella | pneumoniae | SP00318 | Haemophilus | parainfluenzae | 2 |
| SP00173 | Limosilactobacillus | reuteri | SP00196 | Lacticaseibacillus | paracasei | 2 |
| SP00174 | Limosilactobacillus | fermentum | SP00181 | Levilactobacillus | brevis | 2 |
| SP00174 | Limosilactobacillus | fermentum | SP00196 | Lacticaseibacillus | paracasei | 2 |
| SP00174 | Limosilactobacillus | fermentum | SP00237 | Lentilactobacillus | buchneri | 2 |
| SP00174 | Limosilactobacillus | fermentum | SP00243 | Lacticaseibacillus | rhamnosus | 2 |
| SP00186 | Staphylococcus | schleiferi | SP00179 | Bacillus | subtilis | 2 |
| SP00179 | Bacillus | subtilis | SP00248 | Staphylococcus | warneri | 2 |
| SP00179 | Bacillus | subtilis | SP00252 | Staphylococcus | pasteuri | 2 |
| SP00179 | Bacillus | subtilis | SP00258 | Staphylococcus | capitis | 2 |
| SP00179 | Bacillus | subtilis | SP00279 | Staphylococcus | haemolyticus | 2 |
| SP00179 | Bacillus | subtilis | SP00280 | Staphylococcus | lugdunensis | 2 |
| SP00179 | Bacillus | subtilis | SP00302 | Staphylococcus | pettenkoferi | 2 |
| SP00184 | Streptococcus | anginosus | SP00288 | Streptococcus | sp. oral taxon 431 | 2 |
| SP00184 | Streptococcus | anginosus | SP00315 | Streptococcus | mitis | 2 |
| SP00184 | Streptococcus | anginosus | SP00319 | Streptococcus | oralis | 2 |
| SP00190 | Haemophilus | influenzae | SP00185 | Aggregatibacter | actinomycetemcomitans | 2 |
| SP00204 | Delftia | acidovorans | SP00211 | Acidovorax | ebreus | 2 |
| SP00204 | Delftia | acidovorans | SP00268 | Ottowia | sp. oral taxon 894 | 2 |
| SP00205 | Klebsiella | variicola | SP00318 | Haemophilus | parainfluenzae | 2 |
| SP00250 | Burkholderia | cepacia | SP00206 | Ralstonia | pickettii | 2 |
| SP00214 | Micrococcus | luteus | SP00219 | Kytococcus | sedentarius | 2 |
| SP00214 | Micrococcus | luteus | SP00270 | Lawsonella | clevelandensis | 2 |
| SP00214 | Micrococcus | luteus | SP00277 | Janibacter | indicus | 2 |
| SP00214 | Micrococcus | luteus | SP00301 | Kocuria | rhizophila | 2 |
| SP00218 | Leptotrichia | buccalis | SP00271 | Leptotrichia | sp. oral taxon 212 | 2 |
| SP00218 | Leptotrichia | buccalis | SP00285 | Leptotrichia | sp. oral taxon 847 | 2 |
| SP00219 | Kytococcus | sedentarius | SP00265 | Arsenicicoccus | sp. oral taxon 190 | 2 |
| SP00224 | Sanguibacter | keddieii | SP00270 | Lawsonella | clevelandensis | 2 |
| SP00224 | Sanguibacter | keddieii | SP00272 | Kocuria | palustris | 2 |
| SP00227 | Moraxella | catarrhalis | SP00287 | Moraxella | osloensis | 2 |
| SP00232 | Rothia | dentocariosa | SP00272 | Kocuria | palustris | 2 |
| SP00236 | Prevotella | denticola | SP00269 | Prevotella | fusca | 2 |
| SP00240 | Klebsiella | aerogenes | SP00318 | Haemophilus | parainfluenzae | 2 |
| SP00242 | Streptococcus | salivarius | SP00315 | Streptococcus | mitis | 2 |
| SP00242 | Streptococcus | salivarius | SP00319 | Streptococcus | oralis | 2 |
| SP00250 | Burkholderia | cepacia | SP00261 | Cupriavidus | gilardii | 2 |
| SP00259 | Corynebacterium | sp. ATCC 6931 | SP00310 | Dietzia | sp. oral taxon 368 | 2 |
| SP00270 | Lawsonella | clevelandensis | SP00277 | Janibacter | indicus | 2 |
| SP00271 | Leptotrichia | sp. oral taxon 212 | SP00291 | Leptotrichia | sp. oral taxon 498 | 2 |
| SP00272 | Kocuria | palustris | SP00277 | Janibacter | indicus | 2 |
| SP00275 | Serratia | marcescens | SP00318 | Haemophilus | parainfluenzae | 2 |
| SP00277 | Janibacter | indicus | SP00301 | Kocuria | rhizophila | 2 |
| SP00285 | Leptotrichia | sp. oral taxon 847 | SP00291 | Leptotrichia | sp. oral taxon 498 | 2 |
| SP00286 | Actinomyces | oris | SP00309 | Actinomyces | sp. oral taxon 897 | 2 |
| SP00309 | Actinomyces | sp. oral taxon 897 | SP00312 | Actinomyces | sp. oral taxon 171 | 2 |
| SP00309 | Actinomyces | sp. oral taxon 897 | SP00314 | Actinomyces | sp. oral taxon 169 | 2 |
| SP00315 | Streptococcus | mitis | SP00320 | Streptococcus | thermophilus | 2 |
| SP00319 | Streptococcus | oralis | SP00320 | Streptococcus | thermophilus | 2 |
| SP00142 | Pseudomonas | aeruginosa | SP00166 | Pseudomonas | fluorescens | 1 |
| SP00142 | Pseudomonas | aeruginosa | SP00191 | Pseudomonas | protegens | 1 |
| SP00184 | Streptococcus | anginosus | SP00146 | Streptococcus | pneumoniae | 1 |
| SP00146 | Streptococcus | pneumoniae | SP00242 | Streptococcus | salivarius | 1 |
| SP00146 | Streptococcus | pneumoniae | SP00320 | Streptococcus | thermophilus | 1 |
| SP00147 | Agrobacterium | fabrum | SP00170 | Mesorhizobium | japonicum | 1 |
| SP00148 | Streptococcus | agalactiae | SP00242 | Streptococcus | salivarius | 1 |
| SP00148 | Streptococcus | agalactiae | SP00253 | Streptococcus | cristatus | 1 |
| SP00148 | Streptococcus | agalactiae | SP00320 | Streptococcus | thermophilus | 1 |
| SP00169 | Proteus | mirabilis | SP00150 | Yersinia | pestis | 1 |
| SP00151 | Streptococcus | mutans | SP00288 | Streptococcus | sp. oral taxon 431 | 1 |
| SP00151 | Streptococcus | mutans | SP00315 | Streptococcus | mitis | 1 |
| SP00151 | Streptococcus | mutans | SP00319 | Streptococcus | oralis | 1 |
| SP00152 | Bifidobacterium | longum | SP00226 | Gardnerella | vaginalis | 1 |
| SP00155 | Enterococcus | faecalis | SP00162 | Ligilactobacillus | salivarius | 1 |
| SP00155 | Enterococcus | faecalis | SP00163 | Staphylococcus | aureus | 1 |
| SP00155 | Enterococcus | faecalis | SP00251 | Streptococcus | constellatus | 1 |
| SP00155 | Enterococcus | faecalis | SP00280 | Staphylococcus | lugdunensis | 1 |
| SP00158 | Lactobacillus | johnsonii | SP00181 | Levilactobacillus | brevis | 1 |
| SP00158 | Lactobacillus | johnsonii | SP00237 | Lentilactobacillus | buchneri | 1 |
| SP00178 | Rothia | mucilaginosa | SP00214 | Micrococcus | luteus | 1 |
| SP00178 | Rothia | mucilaginosa | SP00219 | Kytococcus | sedentarius | 1 |
| SP00178 | Rothia | mucilaginosa | SP00224 | Sanguibacter | keddieii | 1 |
| SP00178 | Rothia | mucilaginosa | SP00301 | Kocuria | rhizophila | 1 |
| SP00179 | Bacillus | subtilis | SP00297 | Staphylococcus | cohnii | 1 |
| SP00181 | Levilactobacillus | brevis | SP00189 | Lactobacillus | acidophilus | 1 |
| SP00181 | Levilactobacillus | brevis | SP00195 | Lactobacillus | gasseri | 1 |
| SP00181 | Levilactobacillus | brevis | SP00235 | Lactobacillus | amylovorus | 1 |
| SP00181 | Levilactobacillus | brevis | SP00297 | Staphylococcus | cohnii | 1 |
| SP00189 | Lactobacillus | acidophilus | SP00237 | Lentilactobacillus | buchneri | 1 |
| SP00192 | Bifidobacterium | breve | SP00226 | Gardnerella | vaginalis | 1 |
| SP00195 | Lactobacillus | gasseri | SP00237 | Lentilactobacillus | buchneri | 1 |
| SP00211 | Acidovorax | ebreus | SP00268 | Ottowia | sp. oral taxon 894 | 1 |
| SP00213 | Corynebacterium | kroppenstedtii | SP00310 | Dietzia | sp. oral taxon 368 | 1 |
| SP00214 | Micrococcus | luteus | SP00232 | Rothia | dentocariosa | 1 |
| SP00216 | Variovorax | paradoxus | SP00268 | Ottowia | sp. oral taxon 894 | 1 |
| SP00219 | Kytococcus | sedentarius | SP00224 | Sanguibacter | keddieii | 1 |
| SP00219 | Kytococcus | sedentarius | SP00232 | Rothia | dentocariosa | 1 |
| SP00223 | Bifidobacterium | dentium | SP00226 | Gardnerella | vaginalis | 1 |
| SP00224 | Sanguibacter | keddieii | SP00232 | Rothia | dentocariosa | 1 |
| SP00230 | Prevotella | melaninogenica | SP00276 | Prevotella | enoeca | 1 |
| SP00232 | Rothia | dentocariosa | SP00265 | Arsenicicoccus | sp. oral taxon 190 | 1 |
| SP00232 | Rothia | dentocariosa | SP00301 | Kocuria | rhizophila | 1 |
| SP00235 | Lactobacillus | amylovorus | SP00237 | Lentilactobacillus | buchneri | 1 |
| SP00237 | Lentilactobacillus | buchneri | SP00297 | Staphylococcus | cohnii | 1 |
| SP00239 | Pseudopropionibacterium | propionicum | SP00254 | Cutibacterium | avidum | 1 |
| SP00242 | Streptococcus | salivarius | SP00288 | Streptococcus | sp. oral taxon 431 | 1 |
| SP00260 | Acinetobacter | johnsonii | SP00287 | Moraxella | osloensis | 1 |
| SP00270 | Lawsonella | clevelandensis | SP00310 | Dietzia | sp. oral taxon 368 | 1 |
| SP00288 | Streptococcus | sp. oral taxon 431 | SP00320 | Streptococcus | thermophilus | 1 |
| ***TOTAL*** | | | | | | 4450 |

The Table details all the pairs of bacterial species which had *in-silico* amplicon similarity values ≥97% using the bacterial-specific and the bacterial and archaeal primer pairs analyzed in the present study. Frequency= frequency, number times that a pair of species had *in-silico* amplicon similarity values ≥97% in the different primer pairs; ID= species identifier.

Supplementary Table 4. Pairs of different bacterial genera, families, and orders with *in-silico* amplicon similarity values ≥97%.

| **Pair of different genera** | **Frequency** |
| --- | --- |
| Cronobacter\|Klebsiella | 99 |
| Klebsiella\|Serratia | 67 |
| Escherichia\|Klebsiella | 39 |
| Cronobacter\|Escherichia | 36 |
| Aggregatibacter\|Haemophilus | 28 |
| Achromobacter\|Bordetella | 25 |
| Cutibacterium\|Propionibacterium | 24 |
| Cupriavidus\|Ralstonia | 23 |
| Klebsiella\|Yersinia | 23 |
| Listeria\|Staphylococcus | 23 |
| Bacillus\|Staphylococcus | 22 |
| Cronobacter\|Serratia | 22 |
| Actinomyces\|Schaalia | 21 |
| Mycobacterium\|Mycolicibacterium | 21 |
| Escherichia\|Serratia | 20 |
| Acidovorax\|Comamonas | 18 |
| Serratia\|Yersinia | 16 |
| Cronobacter\|Yersinia | 14 |
| Lentilactobacillus\|Levilactobacillus | 14 |
| Janibacter\|Kytococcus | 13 |
| Paracoccus\|Rhodobacter | 13 |
| Lacticaseibacillus\|Lactiplantibacillus | 12 |
| Lacticaseibacillus\|Lentilactobacillus | 11 |
| Lacticaseibacillus\|Levilactobacillus | 11 |
| Lacticaseibacillus\|Limosilactobacillus | 9 |
| Lentilactobacillus\|Limosilactobacillus | 9 |
| Acidovorax\|Variovorax | 8 |
| Agrobacterium\|Brucella | 8 |
| Comamonas\|Variovorax | 7 |
| Escherichia\|Yersinia | 7 |
| Kocuria\|Rothia | 7 |
| Lactiplantibacillus\|Levilactobacillus | 7 |
| Delftia\|Variovorax | 6 |
| Haemophilus\|Klebsiella | 6 |
| Kocuria\|Micrococcus | 6 |
| Lactiplantibacillus\|Lentilactobacillus | 6 |
| Levilactobacillus\|Limosilactobacillus | 5 |
| Agrobacterium\|Mesorhizobium | 4 |
| Enterococcus\|Listeria | 4 |
| Enterococcus\|Staphylococcus | 4 |
| Janibacter\|Kocuria | 4 |
| Janibacter\|Sanguibacter | 4 |
| Lacticaseibacillus\|Ligilactobacillus | 4 |
| Lactobacillus\|Lentilactobacillus | 4 |
| Lactobacillus\|Levilactobacillus | 4 |
| Arsenicicoccus\|Janibacter | 3 |
| Arsenicicoccus\|Sanguibacter | 3 |
| Bifidobacterium\|Gardnerella | 3 |
| Brucella\|Mesorhizobium | 3 |
| Comamonas\|Delftia | 3 |
| Corynebacterium\|Dietzia | 3 |
| Micrococcus\|Sanguibacter | 3 |
| Acidovorax\|Delftia | 2 |
| Arsenicicoccus\|Kytococcus | 2 |
| Bacillus\|Listeria | 2 |
| Burkholderia\|Cupriavidus | 2 |
| Burkholderia\|Ralstonia | 2 |
| Delftia\|Ottowia | 2 |
| Enterococcus\|Lentilactobacillus | 2 |
| Enterococcus\|Levilactobacillus | 2 |
| Escherichia\|Haemophilus | 2 |
| Haemophilus\|Serratia | 2 |
| Janibacter\|Lawsonella | 2 |
| Janibacter\|Micrococcus | 2 |
| Kocuria\|Sanguibacter | 2 |
| Kytococcus\|Micrococcus | 2 |
| Kytococcus\|Rothia | 2 |
| Lawsonella\|Micrococcus | 2 |
| Lawsonella\|Sanguibacter | 2 |
| Micrococcus\|Rothia | 2 |
| Rothia\|Sanguibacter | 2 |
| Acidovorax\|Ottowia | 1 |
| Acinetobacter\|Moraxella | 1 |
| Arsenicicoccus\|Rothia | 1 |
| Cutibacterium\|Pseudopropionibacterium | 1 |
| Dietzia\|Lawsonella | 1 |
| Enterococcus\|Ligilactobacillus | 1 |
| Enterococcus\|Streptococcus | 1 |
| Kytococcus\|Sanguibacter | 1 |
| Lentilactobacillus\|Staphylococcus | 1 |
| Levilactobacillus\|Staphylococcus | 1 |
| Ottowia\|Variovorax | 1 |
| Proteus\|Yersinia | 1 |
| ***TOTAL*** | 809 |
| **Pair of different families** | **Frequency** |
| Enterobacteriaceae\|Yersiniaceae | 153 |
| Listeriaceae\|Staphylococcaceae | 23 |
| Bacillaceae\|Staphylococcaceae | 22 |
| Intrasporangiaceae\|Kytococcaceae | 15 |
| Brucellaceae\|Rhizobiaceae | 8 |
| Enterobacteriaceae\|Pasteurellaceae | 8 |
| Intrasporangiaceae\|Micrococcaceae | 7 |
| Intrasporangiaceae\|Sanguibacteraceae | 7 |
| Micrococcaceae\|Sanguibacteraceae | 7 |
| Enterococcaceae\|Lactobacillaceae | 5 |
| Enterococcaceae\|Listeriaceae | 4 |
| Enterococcaceae\|Staphylococcaceae | 4 |
| Kytococcaceae\|Micrococcaceae | 4 |
| Phyllobacteriaceae\|Rhizobiaceae | 4 |
| Brucellaceae\|Phyllobacteriaceae | 3 |
| Corynebacteriaceae\|Dietziaceae | 3 |
| Bacillaceae\|Listeriaceae | 2 |
| Intrasporangiaceae\|Lawsonellaceae | 2 |
| Lactobacillaceae\|Staphylococcaceae | 2 |
| Lawsonellaceae\|Micrococcaceae | 2 |
| Lawsonellaceae\|Sanguibacteraceae | 2 |
| Pasteurellaceae\|Yersiniaceae | 2 |
| Dietziaceae\|Lawsonellaceae | 1 |
| Enterococcaceae\|Streptococcaceae | 1 |
| Kytococcaceae\|Sanguibacteraceae | 1 |
| Morganellaceae\|Yersiniaceae | 1 |
| ***TOTAL*** | 293 |
| **Pair of different orders** | **Frequency** |
| Bacillales\|Lactobacillales | 10 |
| Enterobacterales\|Pasteurellales | 10 |
| Corynebacteriales\|Micrococcales | 6 |
| ***TOTAL*** | 26 |

Frequency= frequency, number times that a pair of different genera, families, and orders had *in-silico* amplicon similarity values ≥97% in the different primer pairs.

Supplementary Table 5. Pairs of archaeal species with *in-silico* amplicon similarity values ≥97% using the analyzed primer pairs.

| **ID** | **Genus** | **Species** | **ID** | **Genus** | **Species** | **Frequency** |
| --- | --- | --- | --- | --- | --- | --- |
| SP00005 | Methanocaldococcus | jannaschii | SP00043 | Methanocaldococcus | Fervens | 20 |
| SP00005 | Methanocaldococcus | jannaschii | SP00052 | Methanocaldococcus | sp. FS406-22 | 20 |
| SP00005 | Methanocaldococcus | jannaschii | SP00119 | Methanocaldococcus | Bathoardescens | 20 |
| SP00030 | Methanobrevibacter | smithii | SP00132 | Methanobrevibacter | Millerae | 20 |
| SP00043 | Methanocaldococcus | fervens | SP00052 | Methanocaldococcus | sp. FS406-22 | 20 |
| SP00043 | Methanocaldococcus | fervens | SP00119 | Methanocaldococcus | Bathoardescens | 20 |
| SP00052 | Methanocaldococcus | sp. FS406-22 | SP00119 | Methanocaldococcus | Bathoardescens | 20 |
| SP00006 | Pyrococcus | horikoshii | SP00074 | Pyrococcus | sp. NA2 | 19 |
| SP00006 | Pyrococcus | horikoshii | SP00080 | Pyrococcus | Yayanosii | 19 |
| SP00006 | Pyrococcus | horikoshii | SP00089 | Pyrococcus | sp. ST04 | 19 |
| SP00012 | Methanosarcina | acetivorans | SP00013 | Methanosarcina | Mazei | 19 |
| SP00012 | Methanosarcina | acetivorans | SP00017 | Methanosarcina | Barkeri | 19 |
| SP00012 | Methanosarcina | acetivorans | SP00120 | Methanosarcina | Thermophila | 19 |
| SP00012 | Methanosarcina | acetivorans | SP00121 | Methanosarcina | sp. WWM596 | 19 |
| SP00012 | Methanosarcina | acetivorans | SP00122 | Methanosarcina | sp. WH1 | 19 |
| SP00012 | Methanosarcina | acetivorans | SP00124 | Methanosarcina | Siciliae | 19 |
| SP00012 | Methanosarcina | acetivorans | SP00126 | Methanosarcina | Horonobensis | 19 |
| SP00012 | Methanosarcina | acetivorans | SP00128 | Methanosarcina | Vacuolata | 19 |
| SP00012 | Methanosarcina | acetivorans | SP00129 | Methanosarcina | sp. Kolksee | 19 |
| SP00013 | Methanosarcina | mazei | SP00120 | Methanosarcina | Thermophila | 19 |
| SP00013 | Methanosarcina | mazei | SP00124 | Methanosarcina | Siciliae | 19 |
| SP00013 | Methanosarcina | mazei | SP00126 | Methanosarcina | Horonobensis | 19 |
| SP00015 | Haloarcula | marismortui | SP00083 | Haloarcula | Hispanica | 19 |
| SP00015 | Haloarcula | marismortui | SP00131 | Haloarcula | sp. CBA1115 | 19 |
| SP00017 | Methanosarcina | barkeri | SP00120 | Methanosarcina | Thermophila | 19 |
| SP00121 | Methanosarcina | sp. WWM596 | SP00017 | Methanosarcina | Barkeri | 19 |
| SP00122 | Methanosarcina | sp. WH1 | SP00017 | Methanosarcina | Barkeri | 19 |
| SP00124 | Methanosarcina | siciliae | SP00017 | Methanosarcina | Barkeri | 19 |
| SP00126 | Methanosarcina | horonobensis | SP00017 | Methanosarcina | Barkeri | 19 |
| SP00017 | Methanosarcina | barkeri | SP00128 | Methanosarcina | Vacuolata | 19 |
| SP00017 | Methanosarcina | barkeri | SP00129 | Methanosarcina | sp. Kolksee | 19 |
| SP00020 | Methanococcoides | burtonii | SP00127 | Methanococcoides | Methylutens | 19 |
| SP00036 | Thermococcus | onnurineus | SP00041 | Thermococcus | Gammatolerans | 19 |
| SP00036 | Thermococcus | onnurineus | SP00081 | Thermococcus | sp. 4557 | 19 |
| SP00036 | Thermococcus | onnurineus | SP00091 | Thermococcus | Cleftensis | 19 |
| SP00036 | Thermococcus | onnurineus | SP00113 | Thermococcus | Paralvinellae | 19 |
| SP00041 | Thermococcus | gammatolerans | SP00081 | Thermococcus | sp. 4557 | 19 |
| SP00041 | Thermococcus | gammatolerans | SP00091 | Thermococcus | Cleftensis | 19 |
| SP00041 | Thermococcus | gammatolerans | SP00113 | Thermococcus | Paralvinellae | 19 |
| SP00042 | Thermococcus | sibiricus | SP00109 | Thermococcus | Litoralis | 19 |
| SP00044 | Halorhabdus | utahensis | SP00108 | Halorhabdus | Tiamatea | 19 |
| SP00045 | Halomicrobium | mukohataei | SP00133 | Halomicrobium | sp. ZPS1 | 19 |
| SP00067 | Thermococcus | barophilus | SP00113 | Thermococcus | Paralvinellae | 19 |
| SP00074 | Pyrococcus | sp. NA2 | SP00080 | Pyrococcus | Yayanosii | 19 |
| SP00074 | Pyrococcus | sp. NA2 | SP00089 | Pyrococcus | sp. ST04 | 19 |
| SP00080 | Pyrococcus | yayanosii | SP00089 | Pyrococcus | sp. ST04 | 19 |
| SP00081 | Thermococcus | sp. 4557 | SP00091 | Thermococcus | Cleftensis | 19 |
| SP00081 | Thermococcus | sp. 4557 | SP00113 | Thermococcus | Paralvinellae | 19 |
| SP00083 | Haloarcula | hispanica | SP00131 | Haloarcula | sp. CBA1115 | 19 |
| SP00091 | Thermococcus | cleftensis | SP00113 | Thermococcus | Paralvinellae | 19 |
| SP00120 | Methanosarcina | thermophila | SP00124 | Methanosarcina | Siciliae | 19 |
| SP00120 | Methanosarcina | thermophila | SP00126 | Methanosarcina | Horonobensis | 19 |
| SP00120 | Methanosarcina | thermophila | SP00128 | Methanosarcina | Vacuolata | 19 |
| SP00120 | Methanosarcina | thermophila | SP00129 | Methanosarcina | sp. Kolksee | 19 |
| SP00121 | Methanosarcina | sp. WWM596 | SP00122 | Methanosarcina | sp. WH1 | 19 |
| SP00121 | Methanosarcina | sp. WWM596 | SP00124 | Methanosarcina | Siciliae | 19 |
| SP00121 | Methanosarcina | sp. WWM596 | SP00125 | Methanosarcina | Lacustris | 19 |
| SP00121 | Methanosarcina | sp. WWM596 | SP00126 | Methanosarcina | Horonobensis | 19 |
| SP00121 | Methanosarcina | sp. WWM596 | SP00128 | Methanosarcina | Vacuolata | 19 |
| SP00121 | Methanosarcina | sp. WWM596 | SP00129 | Methanosarcina | sp. Kolksee | 19 |
| SP00122 | Methanosarcina | sp. WH1 | SP00124 | Methanosarcina | Siciliae | 19 |
| SP00122 | Methanosarcina | sp. WH1 | SP00125 | Methanosarcina | Lacustris | 19 |
| SP00122 | Methanosarcina | sp. WH1 | SP00126 | Methanosarcina | Horonobensis | 19 |
| SP00122 | Methanosarcina | sp. WH1 | SP00128 | Methanosarcina | Vacuolata | 19 |
| SP00122 | Methanosarcina | sp. WH1 | SP00129 | Methanosarcina | sp. Kolksee | 19 |
| SP00124 | Methanosarcina | siciliae | SP00126 | Methanosarcina | Horonobensis | 19 |
| SP00124 | Methanosarcina | siciliae | SP00128 | Methanosarcina | Vacuolata | 19 |
| SP00124 | Methanosarcina | siciliae | SP00129 | Methanosarcina | sp. Kolksee | 19 |
| SP00126 | Methanosarcina | horonobensis | SP00128 | Methanosarcina | Vacuolata | 19 |
| SP00126 | Methanosarcina | horonobensis | SP00129 | Methanosarcina | sp. Kolksee | 19 |
| SP00128 | Methanosarcina | vacuolata | SP00129 | Methanosarcina | sp. Kolksee | 19 |
| SP00006 | Pyrococcus | horikoshii | SP00036 | Thermococcus | Onnurineus | 18 |
| SP00017 | Methanosarcina | barkeri | SP00125 | Methanosarcina | Lacustris | 18 |
| SP00036 | Thermococcus | onnurineus | SP00067 | Thermococcus | Barophilus | 18 |
| SP00036 | Thermococcus | onnurineus | SP00080 | Pyrococcus | Yayanosii | 18 |
| SP00036 | Thermococcus | onnurineus | SP00089 | Pyrococcus | sp. ST04 | 18 |
| SP00036 | Thermococcus | onnurineus | SP00109 | Thermococcus | Litoralis | 18 |
| SP00037 | Desulfurococcus | amylolyticus | SP00068 | Desulfurococcus | Mucosus | 18 |
| SP00041 | Thermococcus | gammatolerans | SP00067 | Thermococcus | Barophilus | 18 |
| SP00042 | Thermococcus | sibiricus | SP00067 | Thermococcus | Barophilus | 18 |
| SP00042 | Thermococcus | sibiricus | SP00113 | Thermococcus | Paralvinellae | 18 |
| SP00067 | Thermococcus | barophilus | SP00081 | Thermococcus | sp. 4557 | 18 |
| SP00067 | Thermococcus | barophilus | SP00091 | Thermococcus | Cleftensis | 18 |
| SP00067 | Thermococcus | barophilus | SP00109 | Thermococcus | Litoralis | 18 |
| SP00081 | Thermococcus | sp. 4557 | SP00109 | Thermococcus | Litoralis | 18 |
| SP00091 | Thermococcus | cleftensis | SP00109 | Thermococcus | Litoralis | 18 |
| SP00109 | Thermococcus | litoralis | SP00113 | Thermococcus | Paralvinellae | 18 |
| SP00116 | Nitrososphaera | evergladensis (c.) | SP00117 | Nitrososphaera | Viennensis | 18 |
| SP00012 | Methanosarcina | acetivorans | SP00125 | Methanosarcina | Lacustris | 17 |
| SP00017 | Methanosarcina | barkeri | SP00013 | Methanosarcina | Mazei | 17 |
| SP00121 | Methanosarcina | sp. WWM596 | SP00013 | Methanosarcina | Mazei | 17 |
| SP00122 | Methanosarcina | sp. WH1 | SP00013 | Methanosarcina | Mazei | 17 |
| SP00013 | Methanosarcina | mazei | SP00128 | Methanosarcina | Vacuolata | 17 |
| SP00013 | Methanosarcina | mazei | SP00129 | Methanosarcina | sp. Kolksee | 17 |
| SP00018 | Natronomonas | pharaonis | SP00102 | Natronomonas | Moolapensis | 17 |
| SP00036 | Thermococcus | onnurineus | SP00042 | Thermococcus | Sibiricus | 17 |
| SP00036 | Thermococcus | onnurineus | SP00074 | Pyrococcus | sp. NA2 | 17 |
| SP00041 | Thermococcus | gammatolerans | SP00042 | Thermococcus | Sibiricus | 17 |
| SP00041 | Thermococcus | gammatolerans | SP00109 | Thermococcus | Litoralis | 17 |
| SP00042 | Thermococcus | sibiricus | SP00081 | Thermococcus | sp. 4557 | 17 |
| SP00042 | Thermococcus | sibiricus | SP00091 | Thermococcus | Cleftensis | 17 |
| SP00042 | Thermococcus | sibiricus | SP00111 | Palaeococcus | Pacificus | 17 |
| SP00049 | Haloterrigena | turkmenica | SP00092 | Natrinema | sp. J7-2 | 17 |
| SP00092 | Natrinema | sp. J7-2 | SP00098 | Natrinema | Pellirubrum | 17 |
| SP00124 | Methanosarcina | siciliae | SP00125 | Methanosarcina | Lacustris | 17 |
| SP00125 | Methanosarcina | lacustris | SP00126 | Methanosarcina | Horonobensis | 17 |
| SP00005 | Methanocaldococcus | jannaschii | SP00046 | Methanocaldococcus | Vulcanius | 16 |
| SP00046 | Methanocaldococcus | vulcanius | SP00052 | Methanocaldococcus | sp. FS406-22 | 16 |
| SP00048 | Archaeoglobus | profundus | SP00051 | Ferroglobus | Placidus | 16 |
| SP00070 | Thermoproteus | uzoniensis | SP00084 | Thermoproteus | Tenax | 16 |
| SP00094 | Nitrososphaera | gargensis (c.) | SP00116 | Nitrososphaera | evergladensis (c.) | 16 |
| SP00094 | Nitrososphaera | gargensis (c.) | SP00117 | Nitrososphaera | Viennensis | 16 |
| SP00120 | Methanosarcina | thermophila | SP00125 | Methanosarcina | Lacustris | 16 |
| SP00125 | Methanosarcina | lacustris | SP00128 | Methanosarcina | Vacuolata | 16 |
| SP00125 | Methanosarcina | lacustris | SP00129 | Methanosarcina | sp. Kolksee | 16 |
| SP00013 | Methanosarcina | mazei | SP00125 | Methanosarcina | Lacustris | 15 |
| SP00036 | Thermococcus | onnurineus | SP00111 | Palaeococcus | Pacificus | 15 |
| SP00041 | Thermococcus | gammatolerans | SP00080 | Pyrococcus | Yayanosii | 15 |
| SP00041 | Thermococcus | gammatolerans | SP00089 | Pyrococcus | sp. ST04 | 15 |
| SP00041 | Thermococcus | gammatolerans | SP00111 | Palaeococcus | Pacificus | 15 |
| SP00049 | Haloterrigena | turkmenica | SP00098 | Natrinema | Pellirubrum | 15 |
| SP00069 | Methanobacterium | lacus | SP00076 | Methanobacterium | Paludis | 15 |
| SP00080 | Pyrococcus | yayanosii | SP00091 | Thermococcus | Cleftensis | 15 |
| SP00080 | Pyrococcus | yayanosii | SP00113 | Thermococcus | Paralvinellae | 15 |
| SP00089 | Pyrococcus | sp. ST04 | SP00113 | Thermococcus | Paralvinellae | 15 |
| SP00091 | Thermococcus | cleftensis | SP00111 | Palaeococcus | Pacificus | 15 |
| SP00120 | Methanosarcina | thermophila | SP00121 | Methanosarcina | sp. WWM596 | 15 |
| SP00120 | Methanosarcina | thermophila | SP00122 | Methanosarcina | sp. WH1 | 15 |
| SP00006 | Pyrococcus | horikoshii | SP00113 | Thermococcus | Paralvinellae | 14 |
| SP00027 | Methanoculleus | marisnigri | SP00093 | Methanoculleus | Bourgensis | 14 |
| SP00043 | Methanocaldococcus | fervens | SP00046 | Methanocaldococcus | Vulcanius | 14 |
| SP00046 | Methanocaldococcus | vulcanius | SP00119 | Methanocaldococcus | Bathoardescens | 14 |
| SP00056 | Methanocaldococcus | infernus | SP00119 | Methanocaldococcus | Bathoardescens | 14 |
| SP00109 | Thermococcus | litoralis | SP00111 | Palaeococcus | Pacificus | 14 |
| SP00006 | Pyrococcus | horikoshii | SP00041 | Thermococcus | Gammatolerans | 13 |
| SP00006 | Pyrococcus | horikoshii | SP00067 | Thermococcus | Barophilus | 13 |
| SP00006 | Pyrococcus | horikoshii | SP00091 | Thermococcus | Cleftensis | 13 |
| SP00036 | Thermococcus | onnurineus | SP00118 | Thermococcus | Eurythermalis | 13 |
| SP00041 | Thermococcus | gammatolerans | SP00074 | Pyrococcus | sp. NA2 | 13 |
| SP00041 | Thermococcus | gammatolerans | SP00118 | Thermococcus | Eurythermalis | 13 |
| SP00052 | Methanocaldococcus | sp. FS406-22 | SP00056 | Methanocaldococcus | Infernus | 13 |
| SP00067 | Thermococcus | barophilus | SP00074 | Pyrococcus | sp. NA2 | 13 |
| SP00067 | Thermococcus | barophilus | SP00080 | Pyrococcus | Yayanosii | 13 |
| SP00067 | Thermococcus | barophilus | SP00089 | Pyrococcus | sp. ST04 | 13 |
| SP00074 | Pyrococcus | sp. NA2 | SP00091 | Thermococcus | Cleftensis | 13 |
| SP00074 | Pyrococcus | sp. NA2 | SP00113 | Thermococcus | Paralvinellae | 13 |
| SP00076 | Methanobacterium | paludis | SP00135 | Methanobacterium | Congolense | 13 |
| SP00080 | Pyrococcus | yayanosii | SP00118 | Thermococcus | Eurythermalis | 13 |
| SP00089 | Pyrococcus | sp. ST04 | SP00091 | Thermococcus | Cleftensis | 13 |
| SP00091 | Thermococcus | cleftensis | SP00118 | Thermococcus | Eurythermalis | 13 |
| SP00113 | Thermococcus | paralvinellae | SP00118 | Thermococcus | Eurythermalis | 13 |
| SP00006 | Pyrococcus | horikoshii | SP00118 | Thermococcus | Eurythermalis | 12 |
| SP00069 | Methanobacterium | lacus | SP00135 | Methanobacterium | Congolense | 12 |
| SP00074 | Pyrococcus | sp. NA2 | SP00118 | Thermococcus | Eurythermalis | 12 |
| SP00089 | Pyrococcus | sp. ST04 | SP00118 | Thermococcus | Eurythermalis | 12 |
| SP00067 | Thermococcus | barophilus | SP00118 | Thermococcus | Eurythermalis | 11 |
| SP00109 | Thermococcus | litoralis | SP00118 | Thermococcus | Eurythermalis | 11 |
| SP00006 | Pyrococcus | horikoshii | SP00109 | Thermococcus | Litoralis | 10 |
| SP00024 | Hyperthermus | butylicus | SP00082 | Pyrolobus | Fumarii | 10 |
| SP00067 | Thermococcus | barophilus | SP00111 | Palaeococcus | Pacificus | 10 |
| SP00074 | Pyrococcus | sp. NA2 | SP00109 | Thermococcus | Litoralis | 10 |
| SP00080 | Pyrococcus | yayanosii | SP00109 | Thermococcus | Litoralis | 10 |
| SP00089 | Pyrococcus | sp. ST04 | SP00109 | Thermococcus | Litoralis | 10 |
| SP00111 | Palaeococcus | pacificus | SP00113 | Thermococcus | Paralvinellae | 10 |
| SP00004 | Aeropyrum | pernix | SP00110 | Aeropyrum | Camini | 9 |
| SP00033 | Methanoregula | boonei | SP00097 | Methanoregula | Formicica | 9 |
| SP00049 | Haloterrigena | turkmenica | SP00053 | Natrialba | Magadii | 9 |
| SP00081 | Thermococcus | sp. 4557 | SP00118 | Thermococcus | Eurythermalis | 9 |
| SP00042 | Thermococcus | sibiricus | SP00080 | Pyrococcus | Yayanosii | 8 |
| SP00043 | Methanocaldococcus | fervens | SP00056 | Methanocaldococcus | Infernus | 8 |
| SP00006 | Pyrococcus | horikoshii | SP00042 | Thermococcus | Sibiricus | 7 |
| SP00008 | Halobacterium | salinarum | SP00115 | Halobacterium | sp. DL1 | 7 |
| SP00010 | Pyrobaculum | aerophilum | SP00028 | Pyrobaculum | Arsenaticum | 7 |
| SP00010 | Pyrobaculum | aerophilum | SP00084 | Thermoproteus | Tenax | 7 |
| SP00042 | Thermococcus | sibiricus | SP00118 | Thermococcus | Eurythermalis | 7 |
| SP00080 | Pyrococcus | yayanosii | SP00081 | Thermococcus | sp. 4557 | 7 |
| SP00111 | Palaeococcus | pacificus | SP00118 | Thermococcus | Eurythermalis | 7 |
| SP00006 | Pyrococcus | horikoshii | SP00081 | Thermococcus | sp. 4557 | 6 |
| SP00006 | Pyrococcus | horikoshii | SP00111 | Palaeococcus | Pacificus | 6 |
| SP00007 | Methanococcus | maripaludis | SP00031 | Methanococcus | Vannielii | 6 |
| SP00010 | Pyrobaculum | aerophilum | SP00070 | Thermoproteus | Uzoniensis | 6 |
| SP00028 | Pyrobaculum | arsenaticum | SP00070 | Thermoproteus | Uzoniensis | 6 |
| SP00028 | Pyrobaculum | arsenaticum | SP00084 | Thermoproteus | Tenax | 6 |
| SP00042 | Thermococcus | sibiricus | SP00089 | Pyrococcus | sp. ST04 | 6 |
| SP00071 | Archaeoglobus | veneficus | SP00104 | Archaeoglobus | Sulfaticallidus | 6 |
| SP00074 | Pyrococcus | sp. NA2 | SP00111 | Palaeococcus | Pacificus | 6 |
| SP00080 | Pyrococcus | yayanosii | SP00111 | Palaeococcus | Pacificus | 6 |
| SP00081 | Thermococcus | sp. 4557 | SP00111 | Palaeococcus | Pacificus | 6 |
| SP00089 | Pyrococcus | sp. ST04 | SP00111 | Palaeococcus | Pacificus | 6 |
| SP00003 | Pyrobaculum | oguniense | SP00010 | Pyrobaculum | Aerophilum | 5 |
| SP00003 | Pyrobaculum | oguniense | SP00028 | Pyrobaculum | Arsenaticum | 5 |
| SP00003 | Pyrobaculum | oguniense | SP00070 | Thermoproteus | Uzoniensis | 5 |
| SP00003 | Pyrobaculum | oguniense | SP00084 | Thermoproteus | Tenax | 5 |
| SP00005 | Methanocaldococcus | jannaschii | SP00056 | Methanocaldococcus | Infernus | 5 |
| SP00026 | Staphylothermus | marinus | SP00057 | Staphylothermus | Hellenicus | 5 |
| SP00042 | Thermococcus | sibiricus | SP00074 | Pyrococcus | sp. NA2 | 5 |
| SP00074 | Pyrococcus | sp. NA2 | SP00081 | Thermococcus | sp. 4557 | 5 |
| SP00076 | Methanobacterium | paludis | SP00112 | Methanobacterium | Formicicum | 5 |
| SP00081 | Thermococcus | sp. 4557 | SP00089 | Pyrococcus | sp. ST04 | 5 |
| SP00123 | Methanosarcina | sp. MTP4 | SP00013 | Methanosarcina | Mazei | 4 |
| SP00026 | Staphylothermus | marinus | SP00090 | Thermogladius | Calderae | 4 |
| SP00045 | Halomicrobium | mukohataei | SP00083 | Haloarcula | Hispanica | 4 |
| SP00045 | Halomicrobium | mukohataei | SP00131 | Haloarcula | sp. CBA1115 | 4 |
| SP00057 | Staphylothermus | hellenicus | SP00068 | Desulfurococcus | Mucosus | 4 |
| SP00057 | Staphylothermus | hellenicus | SP00082 | Pyrolobus | Fumarii | 4 |
| SP00057 | Staphylothermus | hellenicus | SP00090 | Thermogladius | Calderae | 4 |
| SP00078 | Halopiger | xanaduensis | SP00092 | Natrinema | sp. J7-2 | 4 |
| SP00078 | Halopiger | xanaduensis | SP00098 | Natrinema | Pellirubrum | 4 |
| SP00083 | Haloarcula | hispanica | SP00133 | Halomicrobium | sp. ZPS1 | 4 |
| SP00112 | Methanobacterium | formicicum | SP00135 | Methanobacterium | Congolense | 4 |
| SP00123 | Methanosarcina | sp. MTP4 | SP00128 | Methanosarcina | Vacuolata | 4 |
| SP00123 | Methanosarcina | sp. MTP4 | SP00129 | Methanosarcina | sp. Kolksee | 4 |
| SP00131 | Haloarcula | sp. CBA1115 | SP00133 | Halomicrobium | sp. ZPS1 | 4 |
| SP00012 | Methanosarcina | acetivorans | SP00123 | Methanosarcina | sp. MTP4 | 3 |
| SP00015 | Haloarcula | marismortui | SP00045 | Halomicrobium | Mukohataei | 3 |
| SP00015 | Haloarcula | marismortui | SP00133 | Halomicrobium | sp. ZPS1 | 3 |
| SP00044 | Halorhabdus | utahensis | SP00045 | Halomicrobium | Mukohataei | 3 |
| SP00044 | Halorhabdus | utahensis | SP00133 | Halomicrobium | sp. ZPS1 | 3 |
| SP00045 | Halomicrobium | mukohataei | SP00108 | Halorhabdus | Tiamatea | 3 |
| SP00051 | Ferroglobus | placidus | SP00071 | Archaeoglobus | Veneficus | 3 |
| SP00053 | Natrialba | magadii | SP00092 | Natrinema | sp. J7-2 | 3 |
| SP00053 | Natrialba | magadii | SP00098 | Natrinema | Pellirubrum | 3 |
| SP00053 | Natrialba | magadii | SP00114 | Halostagnicola | Larsenii | 3 |
| SP00055 | Methanohalophilus | mahii | SP00127 | Methanococcoides | Methylutens | 3 |
| SP00069 | Methanobacterium | lacus | SP00112 | Methanobacterium | Formicicum | 3 |
| SP00108 | Halorhabdus | tiamatea | SP00133 | Halomicrobium | sp. ZPS1 | 3 |
| SP00001 | Ignisphaera | aggregans | SP00024 | Hyperthermus | Butylicus | 2 |
| SP00001 | Ignisphaera | aggregans | SP00026 | Staphylothermus | Marinus | 2 |
| SP00001 | Ignisphaera | aggregans | SP00057 | Staphylothermus | Hellenicus | 2 |
| SP00001 | Ignisphaera | aggregans | SP00082 | Pyrolobus | Fumarii | 2 |
| SP00003 | Pyrobaculum | oguniense | SP00086 | Pyrobaculum | Ferrireducens | 2 |
| SP00005 | Methanocaldococcus | jannaschii | SP00075 | Methanotorris | Igneus | 2 |
| SP00007 | Methanococcus | maripaludis | SP00058 | Methanococcus | Voltae | 2 |
| SP00010 | Pyrobaculum | aerophilum | SP00086 | Pyrobaculum | Ferrireducens | 2 |
| SP00015 | Haloarcula | marismortui | SP00044 | Halorhabdus | Utahensis | 2 |
| SP00015 | Haloarcula | marismortui | SP00108 | Halorhabdus | Tiamatea | 2 |
| SP00017 | Methanosarcina | barkeri | SP00123 | Methanosarcina | sp. MTP4 | 2 |
| SP00018 | Natronomonas | pharaonis | SP00045 | Halomicrobium | Mukohataei | 2 |
| SP00018 | Natronomonas | pharaonis | SP00133 | Halomicrobium | sp. ZPS1 | 2 |
| SP00020 | Methanococcoides | burtonii | SP00055 | Methanohalophilus | Mahii | 2 |
| SP00020 | Methanococcoides | burtonii | SP00079 | Methanosalsum | Zhilinae | 2 |
| SP00024 | Hyperthermus | butylicus | SP00026 | Staphylothermus | Marinus | 2 |
| SP00024 | Hyperthermus | butylicus | SP00034 | Ignicoccus | Hospitalis | 2 |
| SP00024 | Hyperthermus | butylicus | SP00057 | Staphylothermus | Hellenicus | 2 |
| SP00024 | Hyperthermus | butylicus | SP00090 | Thermogladius | Calderae | 2 |
| SP00026 | Staphylothermus | marinus | SP00082 | Pyrolobus | Fumarii | 2 |
| SP00028 | Pyrobaculum | arsenaticum | SP00086 | Pyrobaculum | Ferrireducens | 2 |
| SP00034 | Ignicoccus | hospitalis | SP00082 | Pyrolobus | Fumarii | 2 |
| SP00043 | Methanocaldococcus | fervens | SP00075 | Methanotorris | Igneus | 2 |
| SP00044 | Halorhabdus | utahensis | SP00083 | Haloarcula | Hispanica | 2 |
| SP00044 | Halorhabdus | utahensis | SP00131 | Haloarcula | sp. CBA1115 | 2 |
| SP00045 | Halomicrobium | mukohataei | SP00102 | Natronomonas | Moolapensis | 2 |
| SP00049 | Haloterrigena | turkmenica | SP00078 | Halopiger | Xanaduensis | 2 |
| SP00049 | Haloterrigena | turkmenica | SP00114 | Halostagnicola | Larsenii | 2 |
| SP00050 | Methanobrevibacter | ruminantium | SP00106 | Methanobrevibacter | sp. AbM4 | 2 |
| SP00052 | Methanocaldococcus | sp. FS406-22 | SP00075 | Methanotorris | Igneus | 2 |
| SP00055 | Methanohalophilus | mahii | SP00079 | Methanosalsum | Zhilinae | 2 |
| SP00062 | Methanothermobacter | marburgensis | SP00112 | Methanobacterium | Formicicum | 2 |
| SP00062 | Methanothermobacter | marburgensis | SP00135 | Methanobacterium | Congolense | 2 |
| SP00068 | Desulfurococcus | mucosus | SP00090 | Thermogladius | Calderae | 2 |
| SP00075 | Methanotorris | igneus | SP00119 | Methanocaldococcus | Bathoardescens | 2 |
| SP00079 | Methanosalsum | zhilinae | SP00127 | Methanococcoides | Methylutens | 2 |
| SP00082 | Pyrolobus | fumarii | SP00090 | Thermogladius | Calderae | 2 |
| SP00083 | Haloarcula | hispanica | SP00108 | Halorhabdus | Tiamatea | 2 |
| SP00084 | Thermoproteus | tenax | SP00086 | Pyrobaculum | Ferrireducens | 2 |
| SP00092 | Natrinema | sp. J7-2 | SP00114 | Halostagnicola | Larsenii | 2 |
| SP00096 | Natronobacterium | gregoryi | SP00114 | Halostagnicola | Larsenii | 2 |
| SP00102 | Natronomonas | moolapensis | SP00133 | Halomicrobium | sp. ZPS1 | 2 |
| SP00108 | Halorhabdus | tiamatea | SP00131 | Haloarcula | sp. CBA1115 | 2 |
| SP00121 | Methanosarcina | sp. WWM596 | SP00123 | Methanosarcina | sp. MTP4 | 2 |
| SP00122 | Methanosarcina | sp. WH1 | SP00123 | Methanosarcina | sp. MTP4 | 2 |
| SP00123 | Methanosarcina | sp. MTP4 | SP00124 | Methanosarcina | Siciliae | 2 |
| SP00123 | Methanosarcina | sp. MTP4 | SP00125 | Methanosarcina | Lacustris | 2 |
| SP00123 | Methanosarcina | sp. MTP4 | SP00126 | Methanosarcina | Horonobensis | 2 |
| SP00001 | Ignisphaera | aggregans | SP00034 | Ignicoccus | Hospitalis | 1 |
| SP00002 | Methanolobus | psychrophilus | SP00055 | Methanohalophilus | Mahii | 1 |
| SP00004 | Aeropyrum | pernix | SP00024 | Hyperthermus | Butylicus | 1 |
| SP00004 | Aeropyrum | pernix | SP00026 | Staphylothermus | Marinus | 1 |
| SP00004 | Aeropyrum | pernix | SP00034 | Ignicoccus | Hospitalis | 1 |
| SP00004 | Aeropyrum | pernix | SP00057 | Staphylothermus | Hellenicus | 1 |
| SP00004 | Aeropyrum | pernix | SP00082 | Pyrolobus | Fumarii | 1 |
| SP00010 | Pyrobaculum | aerophilum | SP00064 | Vulcanisaeta | Distributa | 1 |
| SP00018 | Natronomonas | pharaonis | SP00044 | Halorhabdus | Utahensis | 1 |
| SP00018 | Natronomonas | pharaonis | SP00108 | Halorhabdus | Tiamatea | 1 |
| SP00018 | Natronomonas | pharaonis | SP00115 | Halobacterium | sp. DL1 | 1 |
| SP00020 | Methanococcoides | burtonii | SP00100 | Methanomethylovorans | Hollandica | 1 |
| SP00022 | Methanothrix | thermoacetophila | SP00072 | Methanothrix | Soehngenii | 1 |
| SP00024 | Hyperthermus | butylicus | SP00068 | Desulfurococcus | Mucosus | 1 |
| SP00024 | Hyperthermus | butylicus | SP00110 | Aeropyrum | Camini | 1 |
| SP00026 | Staphylothermus | marinus | SP00034 | Ignicoccus | Hospitalis | 1 |
| SP00026 | Staphylothermus | marinus | SP00037 | Desulfurococcus | Amylolyticus | 1 |
| SP00026 | Staphylothermus | marinus | SP00061 | Acidilobus | Saccharovorans | 1 |
| SP00026 | Staphylothermus | marinus | SP00068 | Desulfurococcus | Mucosus | 1 |
| SP00028 | Pyrobaculum | arsenaticum | SP00064 | Vulcanisaeta | Distributa | 1 |
| SP00030 | Methanobrevibacter | smithii | SP00050 | Methanobrevibacter | Ruminantium | 1 |
| SP00031 | Methanococcus | vannielii | SP00077 | Methanothermococcus | Okinawensis | 1 |
| SP00032 | Methanococcus | aeolicus | SP00077 | Methanothermococcus | Okinawensis | 1 |
| SP00034 | Ignicoccus | hospitalis | SP00057 | Staphylothermus | Hellenicus | 1 |
| SP00034 | Ignicoccus | hospitalis | SP00068 | Desulfurococcus | Mucosus | 1 |
| SP00034 | Ignicoccus | hospitalis | SP00090 | Thermogladius | Calderae | 1 |
| SP00034 | Ignicoccus | hospitalis | SP00110 | Aeropyrum | Camini | 1 |
| SP00037 | Desulfurococcus | amylolyticus | SP00057 | Staphylothermus | Hellenicus | 1 |
| SP00037 | Desulfurococcus | amylolyticus | SP00090 | Thermogladius | Calderae | 1 |
| SP00038 | Methanosphaerula | palustris | SP00093 | Methanoculleus | Bourgensis | 1 |
| SP00044 | Halorhabdus | utahensis | SP00102 | Natronomonas | Moolapensis | 1 |
| SP00045 | Halomicrobium | mukohataei | SP00115 | Halobacterium | sp. DL1 | 1 |
| SP00046 | Methanocaldococcus | vulcanius | SP00056 | Methanocaldococcus | Infernus | 1 |
| SP00049 | Haloterrigena | turkmenica | SP00096 | Natronobacterium | Gregoryi | 1 |
| SP00049 | Haloterrigena | turkmenica | SP00099 | Halovivax | Ruber | 1 |
| SP00049 | Haloterrigena | turkmenica | SP00101 | Natronococcus | Occultus | 1 |
| SP00051 | Ferroglobus | placidus | SP00118 | Thermococcus | Eurythermalis | 1 |
| SP00053 | Natrialba | magadii | SP00078 | Halopiger | Xanaduensis | 1 |
| SP00053 | Natrialba | magadii | SP00096 | Natronobacterium | Gregoryi | 1 |
| SP00053 | Natrialba | magadii | SP00099 | Halovivax | Ruber | 1 |
| SP00053 | Natrialba | magadii | SP00101 | Natronococcus | Occultus | 1 |
| SP00057 | Staphylothermus | hellenicus | SP00061 | Acidilobus | Saccharovorans | 1 |
| SP00059 | Methanohalobium | evestigatum | SP00079 | Methanosalsum | Zhilinae | 1 |
| SP00062 | Methanothermobacter | marburgensis | SP00069 | Methanobacterium | Lacus | 1 |
| SP00064 | Vulcanisaeta | distributa | SP00070 | Thermoproteus | Uzoniensis | 1 |
| SP00064 | Vulcanisaeta | distributa | SP00084 | Thermoproteus | Tenax | 1 |
| SP00068 | Desulfurococcus | mucosus | SP00082 | Pyrolobus | Fumarii | 1 |
| SP00069 | Methanobacterium | lacus | SP00134 | Methanosphaera | Stadtmanae | 1 |
| SP00070 | Thermoproteus | uzoniensis | SP00086 | Pyrobaculum | Ferrireducens | 1 |
| SP00078 | Halopiger | xanaduensis | SP00096 | Natronobacterium | Gregoryi | 1 |
| SP00078 | Halopiger | xanaduensis | SP00099 | Halovivax | Ruber | 1 |
| SP00078 | Halopiger | xanaduensis | SP00101 | Natronococcus | Occultus | 1 |
| SP00078 | Halopiger | xanaduensis | SP00114 | Halostagnicola | Larsenii | 1 |
| SP00092 | Natrinema | sp. J7-2 | SP00096 | Natronobacterium | Gregoryi | 1 |
| SP00092 | Natrinema | sp. J7-2 | SP00099 | Halovivax | Ruber | 1 |
| SP00092 | Natrinema | sp. J7-2 | SP00101 | Natronococcus | Occultus | 1 |
| SP00096 | Natronobacterium | gregoryi | SP00098 | Natrinema | Pellirubrum | 1 |
| SP00096 | Natronobacterium | gregoryi | SP00101 | Natronococcus | Occultus | 1 |
| SP00098 | Natrinema | pellirubrum | SP00099 | Halovivax | Ruber | 1 |
| SP00098 | Natrinema | pellirubrum | SP00101 | Natronococcus | Occultus | 1 |
| SP00098 | Natrinema | pellirubrum | SP00114 | Halostagnicola | Larsenii | 1 |
| SP00099 | Halovivax | ruber | SP00114 | Halostagnicola | Larsenii | 1 |
| SP00101 | Natronococcus | occultus | SP00114 | Halostagnicola | Larsenii | 1 |
| SP00102 | Natronomonas | moolapensis | SP00108 | Halorhabdus | Tiamatea | 1 |
| SP00102 | Natronomonas | moolapensis | SP00115 | Halobacterium | sp. DL1 | 1 |
| SP00115 | Halobacterium | sp. DL1 | SP00133 | Halomicrobium | sp. ZPS1 | 1 |
| ***TOTAL*** | | | | | | 3232 |

The Table details all the pairs of archaeal species which had *in-silico* amplicon similarity values ≥97% using the archaeal-specific and the bacterial and archaeal primer pairs analyzed in the present study. C.= candidatus; Frequency= frequency, number times that a pair of species had *in-silico* amplicon similarity values ≥97% in the different primer pairs; ID= species identifier.

Supplementary Table 6. Pairs of different archaeal genera, families, orders, and classes with *in-silico* amplicon similarity values ≥97%.

| **Pair of different genera** | **Frequency** |
| --- | --- |
| Pyrococcus\|Thermococcus | 428 |
| Palaeococcus\|Thermococcus | 109 |
| Pyrobaculum\|Thermoproteus | 38 |
| Haloterrigena\|Natrinema | 32 |
| Palaeococcus\|Pyrococcus | 24 |
| Haloarcula\|Halomicrobium | 22 |
| Archaeoglobus\|Ferroglobus | 19 |
| Haloarcula\|Halorhabdus | 12 |
| Halomicrobium\|Halorhabdus | 12 |
| Hyperthermus\|Pyrolobus | 10 |
| Haloterrigena\|Natrialba | 9 |
| Halomicrobium\|Natronomonas | 8 |
| Halopiger\|Natrinema | 8 |
| Methanocaldococcus\|Methanotorris | 8 |
| Staphylothermus\|Thermogladius | 8 |
| Desulfurococcus\|Staphylothermus | 7 |
| Natrialba\|Natrinema | 6 |
| Pyrolobus\|Staphylothermus | 6 |
| Methanobacterium\|Methanothermobacter | 5 |
| Methanococcoides\|Methanohalophilus | 5 |
| Halorhabdus\|Natronomonas | 4 |
| Hyperthermus\|Staphylothermus | 4 |
| Ignisphaera\|Staphylothermus | 4 |
| Methanococcoides\|Methanosalsum | 4 |
| Desulfurococcus\|Thermogladius | 3 |
| Halostagnicola\|Natrialba | 3 |
| Halostagnicola\|Natrinema | 3 |
| Acidilobus\|Staphylothermus | 2 |
| Aeropyrum\|Hyperthermus | 2 |
| Aeropyrum\|Ignicoccus | 2 |
| Aeropyrum\|Staphylothermus | 2 |
| Halobacterium\|Halomicrobium | 2 |
| Halobacterium\|Natronomonas | 2 |
| Halopiger\|Haloterrigena | 2 |
| Halostagnicola\|Haloterrigena | 2 |
| Halostagnicola\|Natronobacterium | 2 |
| Halovivax\|Natrinema | 2 |
| Hyperthermus\|Ignicoccus | 2 |
| Hyperthermus\|Ignisphaera | 2 |
| Hyperthermus\|Thermogladius | 2 |
| Ignicoccus\|Pyrolobus | 2 |
| Ignicoccus\|Staphylothermus | 2 |
| Ignisphaera\|Pyrolobus | 2 |
| Methanococcus\|Methanothermococcus | 2 |
| Methanohalophilus\|Methanosalsum | 2 |
| Natrinema\|Natronobacterium | 2 |
| Natrinema\|Natronococcus | 2 |
| Pyrobaculum\|Vulcanisaeta | 2 |
| Pyrolobus\|Thermogladius | 2 |
| Thermoproteus\|Vulcanisaeta | 2 |
| Aeropyrum\|Pyrolobus | 1 |
| Desulfurococcus\|Hyperthermus | 1 |
| Desulfurococcus\|Ignicoccus | 1 |
| Desulfurococcus\|Pyrolobus | 1 |
| Ferroglobus\|Thermococcus | 1 |
| Halopiger\|Halostagnicola | 1 |
| Halopiger\|Halovivax | 1 |
| Halopiger\|Natrialba | 1 |
| Halopiger\|Natronobacterium | 1 |
| Halopiger\|Natronococcus | 1 |
| Halostagnicola\|Halovivax | 1 |
| Halostagnicola\|Natronococcus | 1 |
| Haloterrigena\|Halovivax | 1 |
| Haloterrigena\|Natronobacterium | 1 |
| Haloterrigena\|Natronococcus | 1 |
| Halovivax\|Natrialba | 1 |
| Ignicoccus\|Ignisphaera | 1 |
| Ignicoccus\|Thermogladius | 1 |
| Methanobacterium\|Methanosphaera | 1 |
| Methanococcoides\|Methanomethylovorans | 1 |
| Methanoculleus\|Methanosphaerula | 1 |
| Methanohalobium\|Methanosalsum | 1 |
| Methanohalophilus\|Methanolobus | 1 |
| Natrialba\|Natronobacterium | 1 |
| Natrialba\|Natronococcus | 1 |
| Natronobacterium\|Natronococcus | 1 |
| ***TOTAL*** | 873 |
| **Pair of different families** | **Frequency** |
| Desulfurococcaceae\|Pyrodictiaceae | 27 |
| Haloarculaceae\|Halobacteriaceae | 4 |
| Acidilobaceae\|Desulfurococcaceae | 2 |
| Archaeoglobaceae\|Thermococcaceae | 1 |
| Methanomicrobiaceae\|Methanoregulaceae | 1 |
| ***TOTAL*** | 35 |
| **Pair of different orders** | **Frequency** |
| Acidilobales\|Desulfurococcales | 2 |
| Archaeoglobales\|Thermococcales | 1 |
| ***TOTAL*** | 3 |
| **Pair of different classes** | **Frequency** |
| Archaeoglobi\|Thermococci | 1 |
| ***TOTAL*** | 1 |

Frequency= frequency, number times that a pair of genera, families, orders, and classes had *in-silico* amplicon similarity values ≥97% in the different primer pairs.
